# Supplementary material for: Misleading Advertising of Health-Related Products in Ecuador during the COVID-19 Pandemic
Source: Diseases. 2022 Oct 18;10(4):91. doi: 10.3390/diseases10040091 (PMC9590028; doi:10.3390/diseases10040091)
Supplement: Supplementary file 1 [file diseases-10-00091-s001.zip › diseases-1732199-SI.pdf]

ACUERDO No. 00000179 (SE EXPIDE EL REGLAMENTO PARA LA PUBLICIDAD Y PROMOCIÓN DE MEDICAMENTOS EN GENERAL, PRODUCTOS NATURALES PROCESADOS DE USO MEDICINAL, MEDICAMENTOS HOMEOPÁTICOS Y DISPOSITIVOS MÉDICOS)

EL MINISTRO DE SALUD PÚBLICA

Considerando:

Que la Constitución de la República del Ecuador ordena: Art. 361.- "El Estado ejercerá la rectoría del sistema a través de la autoridad sanitaria nacional, será responsable de formular la política nacional de salud, y normará, regulará y controlará todas las actividades relacionadas con la salud, así como el funcionamiento de las entidades del sector.".

Que la Ley Orgánica de Salud en su Art. 6 numeral 21 manda: Es responsabilidad del Ministerio de Salud Pública: "Regular y controlar toda forma de publicidad y promoción que atente contra la salud e induzcan comportamientos que la afecten negativamente.".

Que el artículo 143 de la ley ibídem dispone que: "La publicidad y promoción de los productos sujetos a registro sanitario deberá ajustarse a su verdadera naturaleza, composición, calidad u origen, de modo tal que se evite toda concepción errónea de sus cualidades o beneficios, lo cual será controlado por la autoridad sanitaria nacional.

Se prohíbe la publicidad por cualquier medio de medicamentos sujetos a venta bajo prescripción.";

Que el artículo 16 del Reglamento a la Ley Orgánica de Salud, expedido mediante Decreto Ejecutivo 1395 de 16 de octubre del 2008, publicado en el Registro Oficial No. 457 de 30 de octubre del 2008 dispone que: "La promoción de los medicamentos debe sustentarse tanto en la información terapéutica aprobada en el Registro Sanitario del producto, como en las evidencias científicas. La empresa responsable de la comercialización debe estar en condiciones de facilitar dichas evidencias científicas a petición de los profesionales de la salud.";

Que el artículo 17 del mismo reglamento dispone "La promoción de otros productos de uso y consumo humano, sujetos a registro sanitario, comprende la organización o patrocinio de toda actividad relacionada con la entrega de esos productos por parte de las empresas para promover el uso y consumo de los mismos; y,

En ejercicio de las atribuciones legales concedidas por los artículos 151 y 154, numeral 1 de la Constitución de la República del Ecuador y el artículo 17 del Estatuto de Régimen Jurídico y Administrativo de la Función Ejecutiva,

Acuerda:

Expedir el REGLAMENTO PARA LA PUBLICIDAD Y PROMOCIÓN DE MEDICAMENTOS EN GENERAL, PRODUCTOS NATURALES PROCESADOS DE USO MEDICINAL, MEDICAMENTOS HOMEOPÁTICOS Y DISPOSITIVOS MÉDICOS.

Capítulo I ÁMBITO DE APLICACIÓN Y OBJETIVO

Art. 1.- Las disposiciones contenidas en el presente reglamento se aplicarán a los medicamentos en general, productos naturales procesados de uso medicinal, medicamentos homeopáticos y dispositivos médicos, que cuenten con registro sanitario de nuestro país.
Art. 2.- La publicidad de: medicamentos en general, productos naturales procesados de uso medicinal, medicamentos homeopáticos y dispositivos médicos dirigida al público en general con el propósito de informar, debe promover el uso racional y manejo adecuado de los productos mencionados.

Únicamente serán sujetos de autorización de publicidad los: medicamentos en general, productos naturales procesados de uso medicinal, medicamentos homeopáticos, y dispositivos médicos clasificados según el certificado de registro sanitario como de venta libre, en concordancia con lo dispuesto en el artículo 143 de la Ley Orgánica de Salud.
Art. 3.- No se autorizará por ningún medio la publicidad de medicamentos en general, productos naturales procesados de uso medicinal, medicamentos homeopáticos, dispositivos médicos, en los siguientes casos:

a) Aquellos que han sido clasificados por el Instituto Nacional de Higiene y Medicina Tropical Dr. Leopoldo Izquieta Pérez (INH) como de venta bajo prescripción médica, de conformidad con lo establecido en el certificado de registro sanitario ecuatoriano;

b) Aquellos medicamentos que contengan sustancias psicotrópicas o estupefacientes; y,

c) Aquellos que por razones de salud pública, epidemias o emergencias sanitarias sean determinados su uso bajo prescripción médica o se suspenda su uso.

Capítulo II
DE LA SOLICITUD Y REQUISITOS PARA LA AUTORIZACIÓN DE LA PUBLICIDAD

Art. 4.- La Dirección General de Salud a través de la Dirección de Control y Mejoramiento en Vigilancia Sanitaria autorizará la publicidad de medicamentos, productos naturales procesados de uso medicinal, medicamentos homeopáticos, y dispositivos médicos clasificados en el certificado de registro sanitario como de venta libre.

Art. 5.- Para efectos de lo señalado en el artículo anterior, el titular del registro sanitario deberá presentar una solicitud dirigida a la Directora/Director de Control y Mejoramiento en Vigilancia Sanitaria conteniendo los siguientes datos:

a) Nombre del producto;

b) Forma farmacéutica, cuando corresponda;

c) Concentración del principio activo, cuando corresponda:

d) Nombre o razón social de la empresa titular del registro sanitario; y,

e) Nombre del químico farmacéutico o bioquímico farmacéutico técnico responsable del producto objeto de la publicidad.

La solicitud deberá ser suscrita por el titular del registro sanitario y por el responsable técnico del establecimiento farmacéutico.

A la solicitud deberá adjuntar lo siguiente:

1. Copia del registro sanitario ecuatoriano vigente del producto.

2. Copia del permiso de funcionamiento vigente del establecimiento.

3. Copia del informe farmacológico del producto, aprobado por el INH.

4. Copia de la etiqueta interna y cuando aplique la externa, aprobadas por el INH.

5. Story board de la publicidad, impreso. Una vez aprobada la solicitud de publicidad deberá ser remitido en medio magnético por la empresa como parte del trámite de aprobación. El contenido del story board debe cumplir con los requisitos establecidos en el presente reglamento y deberá contener la siguiente información básica:

• Nombre del producto, que debe corresponder al que consta en el certificado de registro sanitario.

• Forma farmacéutica, cuando corresponda.

• Medio publicitario que se utilizará para la difusión.

• Posología, cuando corresponda.

• Precauciones de uso.

• Contraindicaciones establecidas en el informe farmacológico, o en normas farmacológicas vigentes.

• Advertencia: "Si los síntomas persisten consulte a su médico", en el caso que corresponda.

Art. 6.- En el caso de que una empresa solicite la publicidad en un mismo story board de dos o más medicamentos, productos naturales procesados de uso medicinal, medicamentos homeopáticos pero que contengan el mismo principio activo; y dispositivos médicos, del mismo titular del registro sanitario, el contenido del story board deberá estar acorde a las características propias de cada producto sin exagerar sus bondades, y debe cumplir con los requisitos establecidos en el presente reglamento.

Art. 7.- El contenido de la publicidad o promoción de: medicamentos, productos naturales procesados de uso medicinal, medicamentos homeopáticos, y dispositivos médicos, de venta libre deberá cumplir con los siguientes requisitos:

a) La publicidad debe promover el uso racional de los medicamentos;

b) Debe señalar las indicaciones terapéuticas o usos del medicamento, los cuales deben estar escritos en idioma castellano utilizando un lenguaje claro que no genere confusión a los consumidores;

c) En el caso de la publicidad de dispositivos médicos debe constar las indicaciones de uso, en idioma castellano, utilizando un lenguaje claro que no genere confusión;

d) La información divulgada debe ser confiable, precisa, verdadera, actualizada y que esté acorde con las indicaciones terapéuticas;

e) Ajustarse al contenido de lo dispuesto en el certificado de registro sanitario así como en el informe farmacológico emitido por el INH, durante la obtención de dicho registro sanitario;

f) La publicidad no debe inducir al uso indiscriminado, innecesario, incorrecto o inadecuado de los medicamentos, productos naturales procesados de uso medicinal, medicamentos homeopáticos, y dispositivos médicos;

g) El uso de frases e imágenes deberán estar acorde a la afección o uso del producto de conformidad con el informe farmacológico aprobado por el INH durante la obtención del registro sanitario, para favorecer la comprensión del público en general;

h) La información contenida en el material publicitario no puede inducir a interpretaciones equívocas capaces de causar una interpretación falsa, errónea y/o confusa en relación al medicamento, productos naturales procesados de uso medicinal, medicamentos homeopáticos y dispositivos médicos;

i) La publicidad no podrá utilizar expresiones que causen miedo o angustia, o sugerir que la salud puede ser afectada por no usar el medicamento;

j) No debe ser engañosa, subliminal o desleal con empresas de la competencia;

k) Cuando se trate de publicidad en medios audiovisuales e impresos como, folletos, volantes, dípticos, trípticos, el contenido de la publicidad deberá ser de fácil lectura y con color que contraste con el fondo del anuncio;

l) Difundir los mensajes en forma clara y pausada cuando se trate de medios radiales;

m) La publicidad en los letreros ubicados en la vía pública, publicidad estática y otros medios similares, el tamaño de la letra utilizada en la información que corresponde a posología, precauciones de uso, contraindicaciones, y advertencia deberá permitir su fácil lectura; y,

n) En los medios cinematográficos, televisión, audiovisuales y otros similares deberá incluirse en forma visible y mantenerse durante el tiempo que permita la lectura completa de los requisitos señalados en el artículo 5 literal d) del presente reglamento. El contraste de la tipografía deberá ser de tal forma que permita su lectura, cualquiera sea el color de fondo.

Capítulo III
DEL PROCEDIMIENTO DE AUTORIZACIÓN DE LA PUBLICIDAD

Art. 8.- La Dirección de Control y Mejoramiento en Vigilancia Sanitaria procederá a la revisión y análisis de la publicidad, verificará el cumplimiento de los requisitos establecidos en el presente reglamento, y comunicará al interesado la decisión de aprobar o no la publicidad de los medicamentos, productos naturales procesados de uso medicinal, medicamentos homeopáticos, y dispositivos médicos de venta libre.

Si la información suministrada no es suficiente o si luego del análisis del contenido de la publicidad fuere necesario realizar modificaciones, la Dirección de Control y Mejoramiento en Vigilancia Sanitaria notificará a la empresa lo que sea pertinente la misma que en el plazo máximo de 30 días deberá presentar el alcance a la información o modificación de la publicidad y se procederá a realizar el análisis correspondiente para aprobar o no la publicidad para los productos antes mencionados, en base al cumplimiento de los requisitos señalados en el presente reglamento.

En caso que la empresa no cumpla con la presentación de la documentación solicitada en el plazo establecido en el inciso precedente, se dará por terminado el trámite y se notificará por escrito al interesado.
Art. 9.- La publicidad de los siguientes productos clasificados como de venta libre: medicamentos, productos naturales procesados de uso medicinal, medicamentos homeopáticos, y dispositivos médicos, podrá realizarse a través de la prensa, radiodifusión, televisión y en general en cualquier otro medio de comunicación masiva, previa autorización de la Autoridad Sanitaria Nacional.
Art. 10.- La publicidad autorizada por la Dirección General de Salud a través de la Dirección de Control y Mejoramiento en Vigilancia Sanitaria tendrá una vigencia de seis meses; durante este período cualquier modificación al material publicitario deberá ser sometida a una nueva autorización, cumpliendo con todos los requisitos establecidos en el presente reglamento.
Art. 11.- Antes de concluir la vigencia de la autorización otorgada para la publicidad, el titular del registro sanitario podrá solicitar una nueva autorización cumpliendo con todos los requisitos establecidos en el presente reglamento.
Art. 12.- Se podrá solicitar criterio técnico a otras Dependencias Técnicas que formen parte del Ministerio de Salud Pública, en el ámbito de su competencia, sobre el contenido de la publicidad de los siguientes productos clasificados como de venta libre: medicamentos, productos naturales procesados de uso medicinal, medicamentos homeopáticos y dispositivos médicos; previo al otorgamiento de la respectiva autorización.
Art. 13.- La Dirección General de Salud a través de la Dirección de Control y Mejoramiento en Vigilancia Sanitaria, revocará la autorización de publicidad de medicamentos, productos naturales procesados de uso medicinal, medicamentos homeopáticos y dispositivos médicos, cuya modalidad sea de venta libre, si el INH modifica la condición de comercialización del producto de venta libre a modalidad de venta bajo prescripción médica; si se detectare algún cambio de los términos con los cuales fue autorizada la publicidad; por salud pública; epidemias o emergencias sanitarias.

Capítulo IV
DE LA PROMOCIÓN Y VISITA MÉDICA

Art. 14.- La promoción de los medicamentos, productos naturales procesados de uso medicinal, medicamentos homeopáticos y dispositivos médicos, debe ser realizada a los profesionales de la salud autorizados para prescribir, a través de los visitadores a médicos.
Art. 15.- La promoción de los medicamentos a través de los visitadores a médicos, debe sustentarse tanto en la información terapéutica aprobada durante la obtención del registro sanitario del producto, como en las evidencias científicas. El titular del registro sanitario, responsable de la comercialización del producto, debe estar en condiciones de facilitar dichas evidencias científicas a petición de los profesionales de la salud autorizados para prescribir.
Art. 16.- Los Visitadores a Médicos deberán tener formación profesional en carreras afines a las ciencias de la salud y farmacéuticas conforme se dispone en el Reglamento a la Ley Orgánica de Salud expedido mediante Decreto Ejecutivo No. 1395 Registro Oficial 457 de 30 de octubre del 2008.

Los visitadores a médicos deberán estar registrados en el Ministerio de Salud Pública y portar el carné correspondiente durante la visita médica.
Art. 17.- En los servicios de salud públicos se debe cumplir con lo establecido en el Art. 15 del Reglamento a la Ley Orgánica de Salud.

Capítulo V
DE LAS PROHIBICIONES

Art. 18.- Queda prohibida la publicidad en los siguientes casos:

a) Publicidad de medicamentos, productos naturales procesados de uso medicinal, medicamentos homeopáticos y dispositivos médicos cuya modalidad de venta sea bajo prescripción médica;

b) Campañas dirigidas al público en general que induzcan el uso de medicamentos de prescripción médica;

c) Publicidad realizada a través de envases, etiquetas, rótulos, empaques, insertos o prospectos de otros productos que acompañen a los medicamentos, productos naturales procesados de uso medicinal, medicamentos homeopáticos, dispositivos médicos de venta libre;

d) Comparación ofensiva para otras marcas, productos, servicios, empresas u organismos;

e) Que se induzca al uso indiscriminado del producto, o respuestas no demostradas científicamente. Que sugiera que la toma del producto debe ser permanente;

f) Que el producto posee propiedades curativas en enfermedades crónicas;

g) Que sugiera que el producto previene la enfermedad y recomiende su uso en personas sanas para mejorar su estado;

h) Que induzca a interpretar que el producto utilizado es la única alternativa expresando frases y/o slogans que no están amparadas en el correspondiente registro sanitario como: "el producto de mayor elección", "el único", "el más frecuentemente recomendado", "el mejor", "totalmente confiable"; "el más efectivo", "famoso", "totalmente seguro", "es bueno", "nuevo" entre otras;

i) Que el contenido publicitario incluya a menores de edad, a excepción de aquellos medicamentos que están dirigidos a ellos y que exista una autorización por escrito de sus padres, de acuerdo a lo establecido en el Código de la Niñez y Adolescencia artículo 52 numeral 1;

j) Se incluya frases como: "demostrado en ensayos clínicos", "clínicamente comprobado", "recomendado por los expertos y/o instituciones"; en caso de la utilización de dichas frases, se debe anexar a la solicitud la información técnica científica que justifiquen su utilización y que estén debidamente aprobadas por el INH en el proceso de obtención del registro sanitario;

k) Cuando incluya mensajes como: "autorizados por la Autoridad Sanitaria Nacional", "Ministerio de Salud Pública";

l) Que la publicidad induzca al uso y consumo de medicamentos, productos naturales procesados de uso medicinal, medicamentos homeopáticos, y dispositivos médicos en base a ofrecimientos de ofertas y premios, incluyendo asociaciones con otros productos;

m) El uso de imágenes censuradas (desnudos o remides-nudos) que promuevan la adquisición de los productos;

n) Si se utilizan imágenes y nombres de profesionales de la salud que recomienden el uso del medicamento;

o) La publicidad realizada directamente en centros comerciales, eventos deportivos, espectáculos públicos, y otros similares;

p) Cuando afecta la imagen de otros productos, o atenta contra el buen nombre de los productos o prestigio de terceros;

q) Cuando intenta crear una situación de rechazo hacia los productos de la competencia o sus usuarios;

r) Cuando menciona principios activos no contenidos en el producto publicitado;

s) Cuando menciona posibles efectos adversos o colaterales de principios activos no contenidos en el producto publicitado; y,

t) Publicidad de medicamentos, productos naturales procesados de uso medicinal, medicamentos homeopáticos y dispositivos médicos, realizada por establecimientos farmacéuticos y de comercialización de estos productos, sin disponer de la autorización otorgada por la Dirección General de Salud a través de la Dirección de Control y Mejoramiento en Vigilancia Sanitaria.

Capítulo VI
DE LA VIGILANCIA, EL CONTROL Y LAS SANCIONES

Art. 19.- La Autoridad Sanitaria Nacional a través de la Dirección de Control y Mejoramiento en Vigilancia Sanitaria y las direcciones provinciales de salud, monitorearán el cumplimiento de los términos en los que se concede la autorización de publicidad que se difunda en cualquier medio de comunicación social, sobre la base de lo dispuesto en la Ley Orgánica de Salud, este reglamento y demás normativa aplicable.

Art. 20.- En caso de existir una publicidad autorizada por la Autoridad Sanitaria Nacional que incumpla con una o más disposiciones del presente reglamento, la Dirección General de Salud concederá por escrito al responsable o representante legal un plazo máximo de 5 días laborables, a partir de la notificación, para que realicen las correcciones correspondientes; en caso de incumplimiento, se ordenará la suspensión y el retiro inmediato de la misma.

Art. 21.- El incumplimiento a las disposiciones establecidas en el presente reglamento, será sancionado de conformidad con lo dispuesto en la Ley Orgánica de Salud.

Capítulo VII
DE LAS DEFINICIONES

Art. 22.- Para efectos de este reglamento, se entiende por:

Forma Farmacéutica.- Forma física que caracteriza al producto farmacéutico terminado como: comprimidos, cápsulas, jarabes, cremas, entre otros.

Medicamento de venta libre.- Medicamento que por su composición y por la acción farmacológica de sus principios activos, es autorizado para ser expendido o dispensado sin prescripción facultativa.

Medicamento de prescripción médica.- Medicamentos prescritos por un profesional de la salud autorizado, y que se necesita de dicha receta o prescripción médica para el acto de la dispensación o expendio; la misma debe ser suscrita por el profesional de la salud facultado para el efecto, incluyendo el sello con los datos del registro del profesional de la salud en el Ministerio de Salud Pública.

Promoción.- Se refiere a todas las actividades informativas y de persuasión desplegadas por los fabricantes y distribuidores de medicamentos, productos naturales procesados de uso medicinal, medicamentos homeopáticos, y dispositivos médicos, dirigida a los prescriptores con el objeto de inducir a la prescripción, al suministro, o a la adquisición de estos productos, a través de la visita médica.

Publicidad.- Toda forma de oferta informática, escrita, visual y otros, dirigida al público en general, destinada a promover la prescripción, dispensación, venta, y uso o consumo de: medicamentos, productos naturales procesados de uso medicinal, medicamentos homeopáticos, y dispositivos médicos, de venta libre.

Registro Sanitario.- Es la certificación otorgada por la autoridad sanitaria nacional, para la importación, exportación y comercialización de los productos de uso y consumo humano señalados en el artículo 137 de la Ley Orgánica de Salud. Dicha certificación es otorgada cuando se cumpla con los requisitos de calidad, seguridad, eficacia y aptitud para consumir y usar dichos productos cumpliendo los trámites establecidos en dicha ley y sus reglamentos.

Story Board.- Conjunto de ilustraciones y narraciones en secuencia con el objeto de servir de guía para contextualizar la publicidad, pre-visualizar una animación o seguir la estructura de una publicidad antes de realizarse o filmarse.

Titular del Registro Sanitario.- Persona natural o jurídica a cuyo nombre es emitido el certificado de registro sanitario y es el responsable jurídico y de la calidad del producto en el país.

Uso racional de los medicamentos.- Es la prescripción del medicamento apropiado, disponible a un precio asequible, correctamente dispensado y administrado a la dosis y durante el tiempo adecuado.

DISPOSICIONES GENERALES

Primera.- La Dirección General de Salud a través de la Dirección de Control y Mejoramiento en Vigilancia Sanitaria y las direcciones provinciales de salud del país supervisarán los contenidos de la publicidad de medicamentos, productos naturales procesados de uso medicinal, medicamentos homeopáticos, cuya modalidad de venta es libre en los diferentes medios de comunicación y si se encontrara alteración o modificación de las mismas en relación a los términos con los cuales fue autorizada, ordenará la suspensión inmediata y se procederá a aplicar la sanción que corresponda de conformidad con lo establecido en la Ley Orgánica de la Salud.

Segunda.- Se prohíbe la publicidad a través de la entrega directa, al público o usuarios, de muestras de: medicamentos, productos naturales procesados de uso medicinal, medicamentos homeopáticos.

Art. Final.- De la ejecución del presente acuerdo ministerial, que entrará en vigencia a partir de su suscripción, sin perjuicio de su publicación en el Registro Oficial, encárguese a la Dirección General de Salud, a la Dirección de Control y Mejoramiento en Vigilancia Sanitaria y a las direcciones provinciales de salud del país.

Dado en el Distrito Metropolitano de Quito, a 10 de marzo del 2011.

FUENTES DE LA PRESENTE EDICIÓN DEL ACUERDO QUE EXPIDE EL REGLAMENTO PARA LA PUBLICIDAD Y PROMOCIÓN DE MEDICAMENTOS EN GENERAL, PRODUCTOS NATURALES PROCESADOS DE USO MEDICINAL, MEDICAMENTOS HOMEOPÁTICOS Y DISPOSITIVOS MÉDICOS

1.- Acuerdo 00000179 (Registro Oficial 416, 30-III-2011).

LEY ORGANICA DE SALUD

Ley 67
Registro Oficial Suplemento 423 de 22-dic.-2006
Ultima modificación: 18-dic.-2015
Estado: Reformado

EL CONGRESO NACIONAL

Considerando:

Que el numeral 20 del artículo 23 de la Constitución Política de la República, consagra la salud como un derecho humano fundamental y el Estado reconoce y garantiza a las personas el derecho a una calidad de vida que asegure la salud, alimentación y nutrición, agua potable, saneamiento ambiental,...;

Que el artículo 42 de la Constitución Política de la República, dispone que "El Estado garantizará el derecho a la salud, su promoción y protección, por medio del desarrollo de la seguridad alimentaria, la provisión de agua potable y saneamiento básico, el fomento de ambientes saludables en lo familiar, laboral y comunitario, y la posibilidad de acceso permanente e ininterrumpido a servicios de salud, conforme a los principios de equidad, universalidad, solidaridad, calidad y eficiencia.";

Que el Código de la Salud aprobado en 1971, contiene disposiciones desactualizadas en relación a los avances en salud pública, en derechos humanos, en ciencia y tecnología, a la situación de salud y enfermedad de la población, entre otros;

Que el actual Código de la Salud ha experimentado múltiples reformas parciales que lo han convertido en un cuerpo legal disperso y desintegrado;

Que ante los actuales procesos de reforma del Estado, del sector salud y de globalización, en los que se encuentra inmerso nuestro país, la legislación debe priorizar los intereses de la salud de la población por sobre los comerciales y económicos;

Que el Ecuador ha ratificado convenios y tratados internacionales que determinan compromisos importantes del país en diferentes materias como derechos humanos, derechos sexuales y reproductivos, derechos de niños, niñas y adolescentes, entre otros;

Que se hace necesario actualizar conceptos normativos en salud, mediante la promulgación de una ley orgánica que garantice la supremacía sobre otras leyes en esta materia; y,

En ejercicio de sus facultades constitucionales y legales expide la siguiente.

LEY ORGANICA DE SALUD

TITULO PRELIMINAR

CAPITULO I
Del derecho a la salud y su protección

Art. 1.- La presente Ley tiene como finalidad regular las acciones que permitan efectivizar el derecho universal a la salud consagrado en la Constitución Política de la República y la ley. Se rige por los principios de equidad, integralidad, solidaridad, universalidad, irrenunciabilidad, indivisibilidad, participación, pluralidad, calidad y eficiencia; con enfoque de derechos, intercultural, de género, generacional y bioético.

Art. 2.- Todos los integrantes del Sistema Nacional de Salud para la ejecución de las actividades relacionadas con la salud, se sujetarán a las disposiciones de esta Ley, sus reglamentos y las normas establecidas por la autoridad sanitaria nacional.

Art. 3.- La salud es el completo estado de bienestar físico, mental y social y no solamente la ausencia de afecciones o enfermedades. Es un derecho humano inalienable, indivisible, irrenunciable e intransigible, cuya protección y garantía es responsabilidad primordial del Estado; y, el resultado de un proceso colectivo de interacción donde Estado, sociedad, familia e individuos convergen para la construcción de ambientes, entornos y estilos de vida saludables.

CAPITULO II
De la autoridad sanitaria nacional, sus competencias y
Responsabilidades

Art. 4.- La autoridad sanitaria nacional es el Ministerio de Salud Pública, entidad a la que corresponde el ejercicio de las funciones de rectoría en salud; así como la responsabilidad de la aplicación, control y vigilancia del cumplimiento de esta Ley; y, las normas que dicte para su plena vigencia serán obligatorias.

Art. 5.- La autoridad sanitaria nacional creará los mecanismos regulatorios necesarios para que los recursos destinados a salud provenientes del sector público, organismos no gubernamentales y de organismos internacionales, cuyo beneficiario sea el Estado o las instituciones del sector público, se orienten a la implementación, seguimiento y evaluación de políticas, planes, programas y proyectos, de conformidad con los requerimientos y las condiciones de salud de la población.

Art. 6.- Es responsabilidad del Ministerio de Salud Pública:

1. Definir y promulgar la política nacional de salud con base en los principios y enfoques establecidos en el artículo 1 de esta Ley, así como aplicar, controlar y vigilar su cumplimiento;
2. Ejercer la rectoría del Sistema Nacional de Salud;
3. Diseñar e implementar programas de atención integral y de calidad a las personas durante todas las etapas de la vida y de acuerdo con sus condiciones particulares;
4. Declarar la obligatoriedad de las inmunizaciones contra determinadas enfermedades, en los términos y condiciones que la realidad epidemiológica nacional y local requiera; definir las normas y el esquema básico nacional de inmunizaciones; y, proveer sin costo a la población los elementos necesarios para cumplirlo;
5. Regular y vigilar la aplicación de las normas técnicas para la detección, prevención, atención integral y rehabilitación, de enfermedades transmisibles, no transmisibles, crónico-degenerativas, discapacidades y problemas de salud pública declarados prioritarios, y determinar las enfermedades transmisibles de notificación obligatoria, garantizando la confidencialidad de la información;

5-A.- Dictar, regular y controlar la correcta aplicación de la normativa para la atención de patologías consideradas como enfermedades catastróficas, así como, dirigir la efectiva aplicación de los programas de atención de las mismas.

6. Formular e implementar políticas, programas y acciones de promoción, prevención y atención integral de salud sexual y salud reproductiva de acuerdo al ciclo de vida que permitan la vigencia, respeto y goce de los derechos, tanto sexuales como reproductivos, y declarar la obligatoriedad de su atención en los términos y condiciones que la realidad epidemiológica nacional y local requiera;
7. Establecer programas de prevención y atención integral en salud contra la violencia en todas sus formas, con énfasis en los grupos vulnerables;
8. Regular, controlar y vigilar la donación, obtención, procesamiento, almacenamiento, distribución, transfusión, uso y calidad de la sangre humana, sus componentes y derivados, en instituciones y organismos públicos y privados, con y sin fines de lucro, autorizados para ello;
9. Regular y controlar el funcionamiento de bancos de células, tejidos y sangre; plantas industriales de hemoderivados y establecimientos de aféresis, públicos y privados; y, promover la creación de

éstos en sus servicios de salud;
10. Emitir políticas y normas para regular y evitar el consumo del tabaco, bebidas alcohólicas y otras sustancias que afectan la salud;
11. Determinar zonas de alerta sanitaria, identificar grupos poblacionales en grave riesgo y solicitar la declaratoria del estado de emergencia sanitaria, como consecuencia de epidemias, desastres u otros que pongan en grave riesgo la salud colectiva;
12. Elaborar el plan de salud en gestión de riesgos en desastres y en sus consecuencias, en coordinación con la Dirección Nacional de Defensa Civil y demás organismos competentes;
13. Regular, vigilar y tomar las medidas destinadas a proteger la salud humana ante los riesgos y daños que pueden provocar las condiciones del ambiente;
14. Regular, vigilar y controlar la aplicación de las normas de bioseguridad, en coordinación con otros organismos competentes;
15. Regular, planificar, ejecutar, vigilar e informar a la población sobre actividades de salud concernientes a la calidad del agua, aire y suelo; y, promocionar espacios y ambientes saludables, en coordinación con los organismos seccionales y otros competentes;
16. Regular y vigilar, en coordinación con otros organismos competentes, las normas de seguridad y condiciones ambientales en las que desarrollan sus actividades los trabajadores, para la prevención y control de las enfermedades ocupacionales y reducir al mínimo los riesgos y accidentes del trabajo;
17. Regular y vigilar las acciones destinadas a eliminar y controlar la proliferación de fauna nociva para la salud humana;
18. Regular y realizar el control sanitario de la producción, importación, distribución, almacenamiento, transporte, comercialización, dispensación y expendio de alimentos procesados, medicamentos y otros productos para uso y consumo humano; así como los sistemas y procedimientos que garanticen su inocuidad, seguridad y calidad, a través del Instituto Nacional de Higiene y Medicina Tropical Dr. Leopoldo Izquieta Pérez y otras dependencias del Ministerio de Salud Pública;
19. Dictar en coordinación con otros organismos competentes, las políticas y normas para garantizar la seguridad alimentaria y nutricional, incluyendo la prevención de trastornos causados por deficiencia de micro nutrientes o alteraciones provocadas por desórdenes alimentarios, con enfoque de ciclo de vida y vigilar el cumplimiento de las mismas;
20. Formular políticas y desarrollar estrategias y programas para garantizar el acceso y la disponibilidad de medicamentos de calidad, al menor costo para la población, con énfasis en programas de medicamentos genéricos;
21. Regular y controlar toda forma de publicidad y promoción que atente contra la salud e induzcan comportamientos que la afecten negativamente;
22. Regular, controlar o prohibir en casos necesarios, en coordinación con otros organismos competentes, la producción, importación, comercialización, publicidad y uso de sustancias tóxicas o peligrosas que constituyan riesgo para la salud de las personas;
23. Regular, vigilar y controlar en coordinación con otros organismos competentes, la producción y comercialización de los productos de uso y consumo animal y agrícola que afecten a la salud humana;
24. Regular, vigilar, controlar y autorizar el funcionamiento de los establecimientos y servicios de salud, públicos y privados, con y sin fines de lucro, y de los demás sujetos a control sanitario;
25. Regular y ejecutar los procesos de licenciamiento y certificación; y, establecer las normas para la acreditación de los servicios de salud;
26. Establecer políticas para desarrollar, promover y potenciar la práctica de la medicina tradicional, ancestral y alternativa; así como la investigación, para su buena práctica;
27. Determinar las profesiones, niveles técnicos superiores y auxiliares de salud que deben registrarse para su ejercicio;
28. Diseñar en coordinación con el Ministerio de Educación y Cultura y otras organizaciones competentes, programas de promoción y educación para la salud, a ser aplicados en los establecimientos educativos estatales, privados, municipales y fiscomisionales;
29. Desarrollar y promover estrategias, planes y programas de información, educación y comunicación social en salud, en coordinación con instituciones y organizaciones competentes;
30. Dictar, en su ámbito de competencia, las normas sanitarias para el funcionamiento de los locales y establecimientos públicos y privados de atención a la población;

31. Regular, controlar y vigilar los procesos de donación y trasplante de órganos, tejidos y componentes anatómicos humanos y establecer mecanismos que promuevan la donación voluntaria; así como regular, controlar y vigilar el uso de órtesis, prótesis y otros implantes sintéticos en el cuerpo humano;
32. Participar, en coordinación con el organismo nacional competente, en la investigación y el desarrollo de la ciencia y tecnología en salud, salvaguardando la vigencia de los derechos humanos, bajo principios bioéticos;
33. Emitir las normas y regulaciones sanitarias para la instalación y funcionamiento de cementerios, criptas, crematorios, funerarias, salas de velación y tanatorios;
34. Cumplir y hacer cumplir esta Ley, los reglamentos y otras disposiciones legales y técnicas relacionadas con la salud, así como los instrumentos internacionales de los cuales el Ecuador es signatario.

Estas acciones las ejecutará el Ministerio de Salud Pública, aplicando principios y procesos de desconcentración y descentralización; y,

35. Las demás previstas en la Constitución Política de la República y otras leyes.

Nota: Numeral 5-A agregado por Ley No. 0, publicada en Registro Oficial 625 de 24 de Enero del 2012 .

CAPITULO III
Derechos y deberes de las personas y del Estado en
relación con la salud

Art. 7.- Toda persona, sin discriminación por motivo alguno, tiene en relación a la salud, los siguientes derechos:

a) Acceso universal, equitativo, permanente, oportuno y de calidad a todas las acciones y servicios de salud;
b) Acceso gratuito a los programas y acciones de salud pública, dando atención preferente en los servicios de salud públicos y privados, a los grupos vulnerables determinados en la Constitución Política de la República;
c) Vivir en un ambiente sano, ecológicamente equilibrado y libre de contaminación;
d) Respeto a su dignidad, autonomía, privacidad e intimidad; a su cultura, sus prácticas y usos culturales; así como a sus derechos sexuales y reproductivos;
e) Ser oportunamente informada sobre las alternativas de tratamiento, productos y servicios en los procesos relacionados con su salud, así como en usos, efectos, costos y calidad; a recibir consejería y asesoría de personal capacitado antes y después de los procedimientos establecidos en los protocolos médicos. Los integrantes de los pueblos indígenas, de ser el caso, serán informados en su lengua materna;
f) Tener una historia clínica única redactada en términos precisos, comprensibles y completos; así como la confidencialidad respecto de la información en ella contenida y a que se le entregue su epicrisis;
g) Recibir, por parte del profesional de la salud responsable de su atención y facultado para prescribir, una receta que contenga obligatoriamente, en primer lugar, el nombre genérico del medicamento prescrito;
h) Ejercer la autonomía de su voluntad a través del consentimiento por escrito y tomar decisiones respecto a su estado de salud y procedimientos de diagnóstico y tratamiento, salvo en los casos de urgencia, emergencia o riesgo para la vida de las personas y para la salud pública;
i) Utilizar con oportunidad y eficacia, en las instancias competentes, las acciones para tramitar quejas y reclamos administrativos o judiciales que garanticen el cumplimiento de sus derechos; así como la reparación e indemnización oportuna por los daños y perjuicios causados, en aquellos casos que lo ameriten;
j) Ser atendida inmediatamente con servicios profesionales de emergencia, suministro de medicamentos e insumos necesarios en los casos de riesgo inminente para la vida, en cualquier

establecimiento de salud público o privado, sin requerir compromiso económico ni trámite administrativo previos;
k) Participar de manera individual o colectiva en las actividades de salud y vigilar el cumplimiento de las acciones en salud y la calidad de los servicios, mediante la conformación de veedurías ciudadanas u otros mecanismos de participación social; y, ser informado sobre las medidas de prevención y mitigación de las amenazas y situaciones de vulnerabilidad que pongan en riesgo su vida; y,
l) No ser objeto de pruebas, ensayos clínicos, de laboratorio o investigaciones, sin su conocimiento y consentimiento previo por escrito; ni ser sometida a pruebas o exámenes diagnósticos, excepto cuando la ley expresamente lo determine o en caso de emergencia o urgencia en que peligre su vida.

Art. 8.- Son deberes individuales y colectivos en relación con la salud:

a) Cumplir con las medidas de prevención y control establecidas por las autoridades de salud;
b) Proporcionar información oportuna y veraz a las autoridades de salud, cuando se trate de enfermedades declaradas por la autoridad sanitaria nacional como de notificación obligatoria y responsabilizarse por acciones u omisiones que pongan en riesgo la salud individual y colectiva;
c) Cumplir con el tratamiento y recomendaciones realizadas por el personal de salud para su recuperación o para evitar riesgos a su entorno familiar o comunitario;
d) Participar de manera individual y colectiva en todas las actividades de salud y vigilar la calidad de los servicios mediante la conformación de veedurías ciudadanas y contribuir al desarrollo de entornos saludables a nivel laboral, familiar y comunitario; y,
e) Cumplir las disposiciones de esta Ley y sus reglamentos.

Art. 9.- Corresponde al Estado garantizar el derecho a la salud de las personas, para lo cual tiene, entre otras, las siguientes responsabilidades:

a) Establecer, cumplir y hacer cumplir las políticas de Estado, de protección social y de aseguramiento en salud a favor de todos los habitantes del territorio nacional;
b) Establecer programas y acciones de salud pública sin costo para la población;
c) Priorizar la salud pública sobre los intereses comerciales y económicos;
d) Adoptar las medidas necesarias para garantizar en caso de emergencia sanitaria, el acceso y disponibilidad de insumos y medicamentos necesarios para afrontarla, haciendo uso de los mecanismos previstos en los convenios y tratados internacionales y la legislación vigente;
e) Establecer a través de la autoridad sanitaria nacional, los mecanismos que permitan a la persona como sujeto de derechos, el acceso permanente e ininterrumpido, sin obstáculos de ninguna clase a acciones y servicios de salud de calidad;
f) Garantizar a la población el acceso y disponibilidad de medicamentos de calidad a bajo costo, con énfasis en medicamentos genéricos en las presentaciones adecuadas, según la edad y la dotación oportuna, sin costo para el tratamiento del VIH-SIDA y enfermedades como hepatitis, dengue, tuberculosis, malaria y otras transmisibles que pongan en riesgo la salud colectiva;
g) Impulsar la participación de la sociedad en el cuidado de la salud individual y colectiva; y, establecer mecanismos de veeduría y rendición de cuentas en las instituciones públicas y privadas involucradas;
h) Garantizar la asignación fiscal para salud, en los términos señalados por la Constitución Política de la República, la entrega oportuna de los recursos y su distribución bajo el principio de equidad; así como los recursos humanos necesarios para brindar atención integral de calidad a la salud individual y colectiva; e,
i) Garantizar la inversión en infraestructura y equipamiento de los servicios de salud que permita el acceso permanente de la población a atención integral, eficiente, de calidad y oportuna para responder adecuadamente a las necesidades epidemiológicas y comunitarias.

LIBRO I
De las acciones de salud

TITULO I

CAPITULO I
Disposiciones comunes

Art. 10.- Quienes forman parte del Sistema Nacional de Salud aplicarán las políticas, programas y normas de atención integral y de calidad, que incluyen acciones de promoción, prevención, recuperación, rehabilitación y cuidados paliativos de la salud individual y colectiva, con sujeción a los principios y enfoques establecidos en el artículo 1 de esta Ley.

Art. 11.- Los programas de estudio de establecimientos de educación pública, privada, municipales y fiscomicionales, en todos sus niveles y modalidades, incluirán contenidos que fomenten el conocimiento de los deberes y derechos en salud, hábitos y estilos de vida saludables, promuevan el auto cuidado, la igualdad de género, la corresponsabilidad personal, familiar y comunitaria para proteger la salud y el ambiente, y desestimulen y prevengan conductas nocivas.

La autoridad sanitaria nacional, en coordinación con el Ministerio de Educación y Cultura, vigilará que los establecimientos educativos públicos, privados, municipales y fiscomisionales, así como su personal, garanticen el cuidado, protección, salud mental y física de sus educandos.

Art. 12.- La comunicación social en salud estará orientada a desarrollar en la población hábitos y estilos de vida saludables, desestimular conductas nocivas, fomentar la igualdad entre los géneros, desarrollar conciencia sobre la importancia del autocuidado y la participación ciudadana en salud.

Los medios de comunicación social, en cumplimiento de lo previsto en la ley, asignarán espacios permanentes, sin costo para el Estado, para la difusión de programas y mensajes educativos e informativos en salud dirigidos a la población, de acuerdo a las producciones que obligatoriamente, para este efecto, elaborará y entregará trimestralmente la autoridad sanitaria nacional.

La autoridad sanitaria nacional regulará y controlará la difusión de programas o mensajes, para evitar que sus contenidos resulten nocivos para la salud física y psicológica de las personas, en especial de niños, niñas y adolescentes.

Concordancias:

CODIGO DE LA NIÑEZ Y ADOLESCENCIA, Arts. 27, 46

Art. 13.- Los planes y programas de salud para los grupos vulnerables señalados en la Constitución Política de la República, incorporarán el desarrollo de la autoestima, promoverán el cumplimiento de sus derechos y se basarán en el reconocimiento de sus necesidades particulares por parte de los integrantes del Sistema Nacional de Salud y la sociedad en general.

Art. 14.- Quienes forman parte del Sistema Nacional de Salud, implementarán planes y programas de salud mental, con base en la atención integral, privilegiando los grupos vulnerables, con enfoque familiar y comunitario, promoviendo la reinserción social de las personas con enfermedad mental.

Art. 15.- La autoridad sanitaria nacional en coordinación con otras instituciones competentes y organizaciones sociales, implementará programas para la prevención oportuna, diagnóstico, tratamiento y recuperación de las alteraciones del crecimiento y desarrollo.

CAPITULO II
De la alimentación y nutrición

Art. 16.- El Estado establecerá una política intersectorial de seguridad alimentaria y nutricional, que propenda a eliminar los malos hábitos alimenticios, respete y fomente los conocimientos y prácticas alimentarias tradicionales, así como el uso y consumo de productos y alimentos propios de cada

región y garantizará a las personas, el acceso permanente a alimentos sanos, variados, nutritivos, inocuos y suficientes.

Esta política estará especialmente orientada a prevenir trastornos ocasionados por deficiencias de micro nutrientes o alteraciones provocadas por desórdenes alimentarios.

Art. 17.- La autoridad sanitaria nacional conjuntamente con los integrantes del Sistema Nacional de Salud, fomentarán y promoverán la lactancia materna durante los primeros seis meses de vida del niño o la niña, procurando su prolongación hasta los dos años de edad.

Garantizará el acceso a leche materna segura o a sustitutivos de ésta para los hijos de madres portadoras de VIH-SIDA.

Concordancias:

CODIGO DE LA NIÑEZ Y ADOLESCENCIA, Arts. 30

Art. 18.- La autoridad sanitaria nacional, en coordinación con los gobiernos seccionales, las cámaras de la producción y centros universitarios desarrollará actividades de información, educación, comunicación y participación comunitaria dirigidas al conocimiento del valor nutricional de los alimentos, su calidad, suficiencia e inocuidad, de conformidad con las normas técnicas que dicte para el efecto el organismo competente y de la presente Ley.

Art. 19.- La autoridad sanitaria nacional velará por la protección de la salud en el control de las enfermedades por deficiencia de yodo, mediante el control y monitoreo de la yodización de la sal para consumo humano.

CAPITULO III
De la salud sexual y la salud reproductiva

Art. 20.- Las políticas y programas de salud sexual y salud reproductiva garantizarán el acceso de hombres y mujeres, incluidos adolescentes, a acciones y servicios de salud que aseguren la equidad de género, con enfoque pluricultural, y contribuirán a erradicar conductas de riesgo, violencia, estigmatización y explotación de la sexualidad.

Art. 21.- El Estado reconoce a la mortalidad materna, al embarazo en adolescentes y al aborto en condiciones de riesgo como problemas de salud pública; y, garantiza el acceso a los servicios públicos de salud sin costo para las usuarias de conformidad con lo que dispone la Ley de Maternidad Gratuita y Atención a la Infancia.

Los problemas de salud pública requieren de una atención integral, que incluya la prevención de las situaciones de riesgo y abarque soluciones de orden educativo, sanitario, social, psicológico, ético y moral, privilegiando el derecho a la vida garantizado por la Constitución.

Concordancias:

CODIGO CIVIL (LIBRO I), Arts. 61

LEY DE MATERNIDAD GRATUITA Y ATENCION A LA INFANCIA, Arts. 1

Art. 22.- Los servicios de salud, públicos y privados, tienen la obligación de atender de manera prioritaria las emergencias obstétricas y proveer de sangre segura cuando las pacientes lo requieran, sin exigencia de compromiso económico ni trámite administrativo previo.

Concordancias:

CODIGO DE LA NIÑEZ Y ADOLESCENCIA, Arts. 30

Art. 23.- Los programas y servicios de planificación familiar, garantizarán el derecho de hombres y mujeres para decidir de manera libre, voluntaria, responsable, autónoma, sin coerción, violencia ni discriminación sobre el número de hijos que puedan procrear, mantener y educar, en igualdad de condiciones, sin necesidad de consentimiento de terceras personas; así como a acceder a la información necesaria para ello.

Concordancias:

CODIGO DE LA NIÑEZ Y ADOLESCENCIA, Arts. 9

Art. 24.- Los anticonceptivos importados por la autoridad sanitaria nacional, requerirán del registro sanitario nacional además del registro sanitario del país de origen, así como el control de calidad y seguridad del producto, previo a su distribución.

Art. 25.- Los integrantes del Sistema Nacional de Salud promoverán y respetarán el conocimiento y prácticas tradicionales de los pueblos indígenas y afroecuatorianos, de las medicinas alternativas, con relación al embarazo, parto, puerperio, siempre y cuando no comprometan la vida e integridad física y mental de la persona.

Art. 26.- Los integrantes del Sistema Nacional de Salud, implementarán acciones de prevención y atención en salud integral, sexual y reproductiva, dirigida a mujeres y hombres, con énfasis en los adolescentes, sin costo para los usuarios en las instituciones públicas.

Art. 27.- El Ministerio de Educación y Cultura, en coordinación con la autoridad sanitaria nacional, con el organismo estatal especializado en género y otros competentes, elaborará políticas y programas educativos de implementación obligatoria en los establecimientos de educación a nivel nacional, para la difusión y orientación en materia de salud sexual y reproductiva, a fin de prevenir el embarazo en adolescentes, VIH-SIDA y otras afecciones de transmisión sexual, el fomento de la paternidad y maternidad responsables y la erradicación de la explotación sexual; y, asignará los recursos suficientes para ello.

Los medios de comunicación deberán cumplir las directrices emanadas de la autoridad sanitaria nacional a fin de que los contenidos que difunden no promuevan la violencia sexual, el irrespeto a la sexualidad y la discriminación de género, por orientación sexual o cualquier otra.

Concordancias:

CODIGO DE LA NIÑEZ Y ADOLESCENCIA, Arts. 46, 47

Art. 28.- Los gobiernos seccionales, en coordinación con la autoridad sanitaria nacional, desarrollarán actividades de promoción, prevención, educación y participación comunitaria en salud sexual y reproductiva, de conformidad con las normas que ella dicte, considerando su realidad local.

Art. 29.- Esta Ley, faculta a los servicios de salud públicos y privados, a interrumpir un embarazo, única y exclusivamente en los casos previstos en el artículo 447 del Código Penal. Estos no podrán negarse a atender a mujeres con aborto en curso o inevitables, debidamente diagnosticados por el profesional responsable de la atención.

Art. 30.- La autoridad sanitaria nacional, con los integrantes del Sistema Nacional de Salud, fomentará y promoverá la planificación familiar, con responsabilidad mutua y en igualdad de condiciones.

CAPITULO IV
De la violencia

Art. 31.- El Estado reconoce a la violencia como problema de salud pública.

Es responsabilidad de la autoridad sanitaria nacional, de los servicios de salud, organismos seccionales, otros organismos competentes y de la sociedad en su conjunto, contribuir a la disminución de todos los tipos de violencia, incluidos los de género, intrafamiliar, sexual y su impacto sobre la salud.

Art. 32.- En todos los casos de violencia intrafamiliar y sexual, y de sus consecuencias, se brindará atención de salud integral a las personas afectadas.

El personal de los servicios de salud tiene la obligación de atender los casos de violencia intrafamiliar y sexual.

Deberán suministrar, entre otros, anticoncepción de emergencia, realizar los procedimientos y aplicar los esquemas profilácticos y terapéuticos necesarios, para detectar y prevenir el riesgo de contraer infecciones de transmisión sexual, especialmente el VIH y hepatitis B, previa consejería y asesoría a la persona afectada, con su consentimiento informado expresado por escrito.

Concordancias:

LEY CONTRA LA VIOLENCIA A LA MUJER Y LA FAMILIA, Arts. 2

Art. 33.- La autoridad sanitaria nacional en coordinación con el Ministerio Fiscal y otros organismos competentes implementará acciones para armonizar las normas de atención e instrumentos de registro de los distintos tipos de violencia y delitos sexuales, unificándolos en un manual de procedimientos de aplicación obligatoria en los distintos niveles de salud y en el Sistema Nacional de Salud.

CAPITULO V
De los accidentes

Art. 34.- La autoridad sanitaria nacional, en coordinación con el Consejo Nacional de Tránsito y Transporte Terrestres, el Ministerio del Trabajo y Empleo, otros organismos competentes, públicos y privados, y los gobiernos seccionales, impulsarán y desarrollarán políticas, programas y acciones para prevenir y disminuir los accidentes de tránsito, laborales, domésticos, industriales y otros; así como para la atención, recuperación, rehabilitación y reinserción social de las personas afectadas.

El Estado reconoce a los accidentes de tránsito como problema de salud pública, en cuanto sus consecuencias afecten la integridad física y mental de las personas.

CAPITULO VI
De los desastres

Art. 35.- La autoridad sanitaria nacional colaborará con los gobiernos seccionales y con los organismos competentes para integrar en el respectivo plan vigente el componente de salud en gestión de riesgos en emergencias y desastres, para prevenir, reducir y controlar los efectos de los desastres y fenómenos naturales y antrópicos.

Art. 36.- Los integrantes del Sistema Nacional de Salud implementarán, en colaboración con los organismos competentes, un sistema permanente y actualizado de información, capacitación y educación en gestión de riesgos en emergencias y desastres, con la participación de la sociedad en su conjunto.

Art. 37.- Todas las instituciones y establecimientos públicos y privados de cualquier naturaleza, deberán contar con un plan de emergencias, mitigación y atención en casos de desastres, en concordancia con el plan formulado para el efecto.

CAPITULO VII

Del tabaco, bebidas alcohólicas, psicotrópicos, estupefacientes y otras substancias que generan dependencia

Art. 38.- Declárase como problema de salud pública al consumo de tabaco y al consumo excesivo de bebidas alcohólicas, así como al consumo de sustancias estupefacientes y psicotrópicas, fuera del ámbito terapéutico.

Es responsabilidad de la autoridad sanitaria nacional, en coordinación con otros organismos competentes, adoptar medidas para evitar el consumo del tabaco y de bebidas alcohólicas, en todas sus formas, así como dotar a la población de un ambiente saludable, para promover y apoyar el abandono de estos hábitos perjudiciales para la salud humana, individual y colectiva.

Los servicios de salud ejecutarán acciones de atención integral dirigidas a las personas afectadas por el consumo y exposición al humo del tabaco, el alcoholismo, o por el consumo nocivo de psicotrópicos, estupefacientes y otras substancias que generan dependencia, orientadas a su recuperación, rehabilitación y reinserción social.

Concordancias:

CODIGO DE LA NIÑEZ Y ADOLESCENCIA, Arts. 27, 78

SECCION I
DEL CONTROL DEL CONSUMO DE PRODUCTOS DEL TABACO

Art. 39.- La autoridad sanitaria nacional en coordinación con el Ministerio de Educación y Cultura, las universidades, los gobiernos seccionales y la sociedad civil, diseñará y ejecutará planes y programas de educación y prevención del consumo del tabaco y sus productos.

Art. 40.-Nota: Artículo derogado por Ley No. 00, publicada en Registro Oficial 497 de 22 de Julio del 2011 .

Art. 41.-Nota: Artículo derogado por Ley No. 00, publicada en Registro Oficial 497 de 22 de Julio del 2011 .

Art. 42.-Nota: Artículo derogado por Ley No. 00, publicada en Registro Oficial 497 de 22 de Julio del 2011 .

Art. 43.-Nota: Artículo derogado por Ley No. 00, publicada en Registro Oficial 497 de 22 de Julio del 2011 .

Art. 44.- Los derechos y obligaciones establecidos en la presente Ley, no excluyen ni se oponen a aquellos contenidos en la legislación destinada a regular la protección del ser humano de la exposición al humo del tabaco, desde antes de su nacimiento, del medio ambiente y el desarrollo sustentable y otras leyes relacionadas con el control del consumo del tabaco.

Concordancias:

CODIGO CIVIL (LIBRO I), Arts. 61

Art. 45.- Las empresas tabacaleras extranjeras que comercialicen sus productos en el Ecuador, deberán contar en el país con un representante legal con plenos poderes para ejercitar derechos y cumplir obligaciones derivadas de la aplicación de la presente Ley.

Concordancias:

LEY DE COMPAÑIAS, Arts. 6, 415

SECCION II
DE LA PREVENCION DEL CONSUMO DE BEBIDAS ALCOHOLICAS

Art. 46.- La autoridad sanitaria nacional en coordinación con el Ministerio de Educación y Cultura, las universidades, los gobiernos seccionales y la sociedad civil, diseñará y ejecutará planes y programas de educación y prevención del consumo de bebidas alcohólicas.

Art. 47.- Se prohíbe la distribución o entrega de bebidas alcohólicas, sea a título gratuito u oneroso, a personas menores de 18 años; así como su venta y consumo en establecimientos educativos, de salud y de expendio de medicamentos.

Concordancias:

CODIGO DE LA NIÑEZ Y ADOLESCENCIA, Arts. 27

Art. 48.- La publicidad de bebidas alcohólicas por ningún motivo se vinculará a la salud, al éxito deportivo o a la imagen de la mujer como símbolo sexual. La autoridad sanitaria nacional vigilará y controlará el cumplimiento de esta disposición.

Art. 49.- Los envases de bebidas alcohólicas, deben incluir de forma clara, visible y comprensible, la advertencia de su carácter nocivo para la salud; y, para la impresión de la advertencia, se seguirán las especificaciones previstas en el reglamento correspondiente.

Art. 50.- Salvo en los actos autorizados por la autoridad competente, se prohíbe consumir bebidas alcohólicas y de moderación, en instituciones públicas, establecimientos educativos, sean públicos o privados, servicios de salud, lugares de trabajo, medios de transporte colectivo, salas de cine y teatro, y otros espacios que se definan en los reglamentos correspondientes emitidos por la autoridad sanitaria nacional. En estos establecimientos se colocarán advertencias visibles que indiquen la prohibición del consumo de bebidas alcohólicas.

Concordancias:

CODIGO DEL TRABAJO, Arts. 46

SECCION III
DEL USO Y CONSUMO DE PSICOTROPICOS, ESTUPEFACIENTES Y OTRAS SUBSTANCIAS QUE GENERAN DEPENDENCIA

Art. 51.- Está prohibido la producción, comercialización, distribución y consumo de estupefacientes y psicotrópicos y otras substancias adictivas, salvo el uso terapéutico y bajo prescripción médica, que serán controlados por la autoridad sanitaria nacional, de acuerdo con lo establecido en la legislación pertinente.

TITULO II
Prevención y control de enfermedades

CAPITULO I
De las inmunizaciones

Art. 52.- La autoridad sanitaria nacional proveerá a los establecimientos de salud los biológicos e insumos para las enfermedades inmunoprevenibles contempladas en el esquema básico nacional de

vacunación, en forma oportuna y permanente, asegurando su calidad y conservación, sin costo al usuario final.

Art. 53.- Es obligación de los servicios de salud y otras instituciones y establecimientos públicos y privados, inmunizar a los trabajadores que se encuentren expuestos a riesgos prevenibles por vacunación, de conformidad con la normativa emitida por la autoridad sanitaria nacional.

Concordancias:

CODIGO DEL TRABAJO, Arts. 410

Art. 54.- El Estado garantizará y transferirá oportunamente, a través del organismo competente, los recursos económicos suficientes para el cumplimiento de las acciones del Programa Ampliado de Inmunizaciones, de conformidad con lo señalado en la ley.

Art. 55.- Los biológicos importados por el Estado a través del Fondo Rotatorio o de los convenios de gobierno a gobierno, no requieren de registro sanitario nacional, siendo obligatorio el del país de origen y la comprobación de la calidad y seguridad del producto antes de su distribución y utilización.

Art. 56.- Los biológicos adquiridos a cualquier título por instituciones públicas o privadas, producidos en el país o importados, serán sometidos a los procesos establecidos por la autoridad sanitaria nacional para liberación de lotes con el fin de preservar su calidad e inocuidad.

Art. 57.- Los biológicos importados por el Ministerio de Salud Pública a su arribo al país, deben pasar al Banco Nacional de Vacunas, en un plazo no mayor de cuarenta y ocho horas, debiéndose garantizar el mantenimiento de la cadena de frío y la calidad de los productos, siendo esto responsabilidad de la autoridad aduanera y de la autoridad sanitaria nacional.

Art. 58.- Las instituciones públicas y privadas de salud administrarán, sin costo a la población, de acuerdo a lo que establezca el reglamento aplicable, los biológicos contemplados en el esquema básico nacional de vacunación, cuando éstos hayan sido suministrados por la autoridad sanitaria nacional.

Las instituciones públicas y privadas reportarán obligatoriamente a la autoridad sanitaria nacional sobre las personas inmunizadas.

Art. 59.- Los padres y madres de familia, tutores o representantes legales de los niños, niñas y adolescentes, entidades educativas, instituciones públicas y privadas con población cautiva en riesgo, tienen la obligación y la responsabilidad de vigilar que se aplique y cumpla el esquema básico nacional de vacunación establecido por la autoridad sanitaria nacional.

Concordancias:

CODIGO CIVIL (LIBRO I), Arts. 268

Art. 60.- Las instituciones públicas y privadas para la administración y expendio de biológicos deben contar con la autorización de la autoridad sanitaria nacional y cumplir con los requisitos establecidos para garantizar una vacuna segura.

CAPITULO II
De las enfermedades transmisibles

Art. 61.- Las instituciones públicas y privadas, los profesionales de salud y la población en general, reportarán en forma oportuna la existencia de casos sospechosos, probables, compatibles y confirmados de enfermedades declaradas por la autoridad sanitaria nacional como de notificación obligatoria y aquellas de reporte internacional. Las instituciones y profesionales de salud,

garantizarán la confidencialidad de la información entregada y recibida.

Art. 62.- La autoridad sanitaria nacional elaborará las normas, protocolos y procedimientos que deben ser obligatoriamente cumplidos y utilizados para la vigilancia epidemiológica y el control de las enfermedades transmisibles, emergentes y reemergentes de notificación obligatoria, incluyendo las de transmisión sexual.

Garantizará en sus servicios de salud, atención, acceso y disponibilidad de medicamentos, con énfasis en genéricos, exámenes de detección y seguimiento, para las enfermedades señaladas en el inciso precedente, lo cual también debe garantizar el sistema nacional de seguridad social.

Art. 63.- La autoridad sanitaria nacional en coordinación con otros organismos competentes ejecutará campañas de información y educación dirigidas al personal de salud y a la población en general, para erradicar actitudes discriminatorias contra las personas afectadas por enfermedades transmisibles.

Art. 64.- En casos de sospecha o diagnóstico de la existencia de enfermedades transmisibles, el personal de salud está obligado a tomar las medidas de bioseguridad y otras necesarias para evitar la transmisión y propagación de conformidad con las disposiciones establecidas por la autoridad sanitaria nacional.

Art. 65.- Los gobiernos seccionales deben cumplir con las disposiciones emanadas por la autoridad sanitaria nacional para evitar la proliferación de vectores, la propagación de enfermedades transmisibles y asegurar el control de las mismas.

Art. 66.- Las personas naturales y jurídicas, nacionales y extranjeras, que se encuentren en territorio ecuatoriano deben cumplir las disposiciones reglamentarias que el gobierno dicte y las medidas que la autoridad sanitaria nacional disponga de conformidad con el Reglamento Sanitario Internacional, los convenios internacionales suscritos y ratificados por el país, a fin de prevenir y evitar la propagación internacional de enfermedades transmisibles.

Concordancias:

CODIGO CIVIL (TITULO PRELIMINAR), Arts. 13

Art. 67.- El Estado reconoce al contagio y la transmisión del VIH-SIDA, como problema de salud pública.

La autoridad sanitaria nacional garantizará en sus servicios de salud a las personas viviendo con VIH-SIDA atención especializada, acceso y disponibilidad de medicamentos antiretrovirales y para enfermedades oportunistas con énfasis en medicamentos genéricos, así como los reactivos para exámenes de detección y seguimiento.

Las responsabilidades señaladas en este artículo corresponden también al sistema nacional de seguridad social.

Art. 68.- Se suministrará la anticoncepción que corresponda, previo consentimiento informado, a mujeres portadoras de VIH y a aquellas viviendo con SIDA. Esto incluye anticoncepción de emergencia cuando el caso lo requiera, a juicio del profesional responsable de la atención.

CAPITULO III
De las enfermedades no transmisibles

Art. 69.- La atención integral y el control de enfermedades no transmisibles, crónico - degenerativas, congénitas, hereditarias y de los problemas declarados prioritarios para la salud pública, se realizará mediante la acción coordinada de todos los integrantes del Sistema Nacional de Salud y de la

participación de la población en su conjunto.

Comprenderá la investigación de sus causas, magnitud e impacto sobre la salud, vigilancia epidemiológica, promoción de hábitos y estilos de vida saludables, prevención, recuperación, rehabilitación, reinserción social de las personas afectadas y cuidados paliativos.

Los integrantes del Sistema Nacional de Salud garantizarán la disponibilidad y acceso a programas y medicamentos para estas enfermedades, con énfasis en medicamentos genéricos, priorizando a los grupos vulnerables.

CAPITULO III-A
DE LAS ENFERMEDADES CATASTROFICAS Y RARAS O HUERFANAS

Nota: Capítulo agregado por Ley No. 0, publicada en Registro Oficial 625 de 24 de Enero del 2012 .

Art. ...(1).- El Estado ecuatoriano reconocerá de interés nacional a las enfermedades catastróficas y raras o huérfanas; y, a través de la autoridad sanitaria nacional, implementará las acciones necesarias para la atención en salud de las y los enfermos que las padezcan, con el fin de mejorar su calidad y expectativa de vida, bajo los principios de disponibilidad, accesibilidad, calidad y calidez; y, estándares de calidad, en la promoción, prevención, diagnóstico, tratamiento, rehabilitación, habilitación y curación.

Las personas que sufran estas enfermedades serán consideradas en condiciones de doble vulnerabilidad.

Nota: Artículo agregado por Ley No. 0, publicada en Registro Oficial 625 de 24 de Enero del 2012 .

Art. ...(2).- Son obligaciones de la autoridad sanitaria nacional:

a) Emitir protocolos para la atención de estas enfermedades, con la participación de las sociedades científicas, las mismas que establecerán las directrices, criterios y procedimientos de diagnóstico y tratamiento de las y los pacientes que padezcan enfermedades raras o huérfanas;
b) Promover, coordinar y desarrollar, conjuntamente con organismos especializados nacionales e internacionales públicos y privados, investigaciones para el estudio de las enfermedades raras o huérfanas y catastróficas con la finalidad de favorecer diagnósticos y tratamientos tempranos en pro de una mejor calidad y expectativa de vida;

En aquellos, casos en los que al Sistema Nacional de Salud le resulte imposible emitir el diagnóstico definitivo de una enfermedad, la autoridad sanitaria nacional implementará todas las acciones para que estos casos sean investigados en instituciones internacionales de la salud con la finalidad de obtener el diagnóstico y tratamiento correspondiente.

c) Controlar y regular, en coordinación con los organismos competentes, a las compañías de seguros y prestadoras de servicios de medicina pre pagada en lo referente a la oferta de coberturas para enfermedades consideradas raras o huérfanas.

Las compañías de seguros y las empresas privadas de salud y medicina pre pagada, en el marco de las políticas definidas por la autoridad sanitaria nacional y de la presente Ley, estarán obligadas a cumplir las coberturas comprometidas en los respectivos contratos de seguro sin que puedan negar dicha cobertura a pretexto del aparecimiento posterior de enfermedades consideradas catastróficas y raras o huérfanas.

d) Controlar que los prestadores de servicios de salud mantengan la búsqueda activa de casos relacionados con las enfermedades raras o huérfanas y catastróficas, de conformidad con el Sistema de Vigilancia Epidemiológica que incluya el registro de los pacientes que sufran este tipo de enfermedades.

e) Implementar las medidas necesarias que faciliten y permitan la adquisición de medicamentos e insumos especiales para el cuidado de enfermedades consideradas raras o huérfanas en forma oportuna, permanente y gratuita para la atención de las personas que padecen enfermedades raras o huérfanas.
f) Establecer, en forma conjunta con las organizaciones de pacientes y científicas, acciones para divulgar y promover el conocimiento de las enfermedades raras y huérfanas.

Nota: Artículo agregado por Ley No. 0, publicada en Registro Oficial 625 de 24 de Enero del 2012 .

Art. ...(3).- La autoridad sanitaria nacional creará e implementará un sistema de registro e información de pacientes que padezcan enfermedades raras o huérfanas y requerirá los reportes que en forma obligatoria deberán remitir todas las instituciones prestadoras de servicios de salud de los sectores públicos y privados respecto de los pacientes que sean diagnosticados o aquellos en los cuales no se pudiere emitir el diagnóstico definitivo.

El organismo encargado de la política migratoria y las instituciones diplomáticas coordinarán con la autoridad sanitaria nacional y con el ministerio encargado de la inclusión económica y social, la implementación del registro de personas residentes en el extranjero que padezcan enfermedades raras o huérfanas, a fin de brindar atención oportuna en el país de residencia y de ser el caso en el territorio nacional.

Nota: Artículo agregado por Ley No. 0, publicada en Registro Oficial 625 de 24 de Enero del 2012 .

Art. ...(4).- La autoridad sanitaria nacional promoverá acciones destinadas a la capacitación, a nivel de pregrado, postgrado y la educación permanente, para todo el personal y profesionales de la salud, a fin de divulgar el conocimiento científico de las enfermedades raras o huérfanas.

Nota: Artículo agregado por Ley No. 0, publicada en Registro Oficial 625 de 24 de Enero del 2012 .

Art. ...(5).- La Autoridad Sanitaria nacional regulará la producción e importación de medicamentos e insumos especiales para tratar enfermedades consideradas raras o huérfanas; y, procurará a través de la normativa que expida para el efecto, la provisión suficiente y necesaria de tales medicamentos para los pacientes según sus necesidades.

La Autoridad Sanitaria nacional promoverá los mecanismos que permitan a las y los pacientes que sufran estas enfermedades, el acceso a los medicamentos e insumos especiales para su tratamiento.

Nota: Artículo agregado por Ley No. 0, publicada en Registro Oficial 625 de 24 de Enero del 2012 .

CAPITULO IV
De la sangre, sus componentes y derivados

Art. 70.- Se declara de prioridad nacional la disponibilidad de sangre segura y sus componentes.

El Estado, a través de la autoridad sanitaria nacional, tomará las medidas necesarias para garantizar la disponibilidad y el acceso a sangre y componentes seguros en cantidades suficientes para quien la necesite, siendo obligatoria su provisión en las instituciones públicas, privadas y autónomas, en caso de riesgo inminente para la vida, independientemente de la capacidad de pago.

La autoridad sanitaria nacional está obligada a promover la donación voluntaria y altruista de sangre.

Art. 71.- La autoridad sanitaria nacional dictará las normas relativas a los procesos de donación, transfusión, uso y vigilancia de la calidad de la sangre humana con sus componentes y derivados, con el fin de garantizar el acceso equitativo, eficiente, suficiente y seguro, la preservación de la salud de los donantes y la máxima protección de los receptores así como del personal de salud.

Art. 72.- La autoridad sanitaria nacional licenciará, a través de la instancia competente, a los servicios de sangre (hemocentros, bancos, depósitos y servicios de transfusión) y a las plantas industriales de fraccionamiento de plasma, públicos y privados, de acuerdo a la normativa vigente.

Art. 73.- Los hemocentros, bancos, depósitos y servicios de transfusión de sangre humana, deben mantener programas de gestión y control de calidad interna y externa, así como cumplir con las demás normas y disposiciones que para el efecto dicte la autoridad sanitaria nacional.

Art. 74.- Se prohíbe la comercialización, publicidad de la misma y el lucro en el proceso de donación, obtención, procesamiento, distribución y utilización de sangre, sus derivados y componentes, por parte de personas naturales o jurídicas, públicas o privadas.

Las instituciones que realicen los procesos señalados en el inciso precedente pueden recuperar únicamente lo correspondiente a gastos de operación de los procedimientos que se realicen; cualquier cobro en exceso será sancionado.

Art. 75.- Los establecimientos autorizados para colectar unidades de sangre, previamente a su utilización en transfusiones, están obligados a realizar las pruebas para determinar el grupo y factor sanguíneo y la presencia de anticuerpos irregulares, así como las serológicas para los marcadores de infección, determinados en la reglamentación correspondiente de acuerdo con el perfil epidemiológico local, regional y nacional y los avances tecnológicos.

La separación de componentes se realizará cumpliendo las normas técnicas aplicables con el fin de asegurar la función terapéutica de los mismos.

Art. 76.- La transfusión de sangre y sus componentes, debe ser prescrita por un médico, legalmente habilitado para ejercer la profesión, practicada bajo su responsabilidad y supervisión, en condiciones que garanticen la seguridad del procedimiento y de conformidad con lo establecido en las normas técnicas.

Art. 77.- La aceptación o negativa para transfusión de sangre y sus componentes, debe realizarse por escrito de parte del potencial receptor o a través de la persona legalmente capaz para ejercer su representación, exceptuándose los casos de emergencia o urgencia.

Concordancias:

CODIGO CIVIL (TITULO PRELIMINAR), Arts. 28

CODIGO CIVIL (LIBRO IV), Arts. 1463

Art. 78.- La donación voluntaria de sangre requiere de la expresa autorización libre, voluntaria y por escrito del donante.

Concordancias:

CODIGO CIVIL (LIBRO IV), Arts. 1461

Art. 79.- La exportación de plasma para procesamiento industrial sólo podrá realizarse hacia plantas procesadoras acreditadas y siempre que los derivados obtenidos sean recuperados para consumo nacional.

Art. 80.- Prohíbese la exportación de sangre y sus componentes, salvo casos expresos de donación originados por razones de emergencia y humanitarias según lo señalado en el artículo anterior.

TITULO III
De los trasplantes de órganos, tejidos y disposición de cadáveres

CAPITULO I
De los trasplantes de órganos y tejidos

Art. 81.- Prohíbese la comercialización de componentes anatómicos de personas vivas o fallecidas. Ninguna persona podrá ofrecer o recibir directa o indirectamente beneficios económicos o de otra índole, por la entrega u obtención de órganos y otros componentes anatómicos de personas vivas o fallecidas.

Concordancias:

LEY ORGANICA DE DONACION Y TRASPLANTE DE ORGANOS, TEJIDOS Y CELULAS, Arts. 73

Art. 82.-Nota: Artículo derogado por Ley No. 00, publicada en Registro Oficial 398 de 4 de Marzo del 2011 .

Art. 83.-Nota: Artículo derogado por Ley No. 00, publicada en Registro Oficial 398 de 4 de Marzo del 2011 .

Art. 84.- La autoridad sanitaria nacional, normará, licenciará y controlará el funcionamiento de los servicios de salud especializados, públicos y privados, para el ejercicio de actividades relacionadas con el trasplante de órganos u otros componentes anatómicos. Igualmente controlará el ejercicio profesional de quienes realicen dichas actividades.

Art. 85.- La autoridad sanitaria nacional normará la organización de los bancos de tejidos y de células, bajo los parámetros técnicos y estándares que se establezcan para el efecto.

La asignación de órganos u otros componentes anatómicos debe realizarse bajo los parámetros nacionales e internacionales, establecidos por un sistema nacional creado para este efecto.

Concordancias:

LEY ORGANICA DE DONACION Y TRASPLANTE DE ORGANOS, TEJIDOS Y CELULAS, Arts. 3, 10, 48

Art. 86.- Los xenotrasplantes podrán realizarse únicamente cuando se garanticen condiciones científicas y tecnológicas que aseguren la calidad del procedimiento con sujeción a principios bioéticos, y estarán sujetos a la autorización de la autoridad sanitaria nacional a través del organismo competente.

CAPITULO II
De la disposición y manejo de cadáveres

Art. 87.- La instalación, construcción y mantenimiento de cementerios, criptas, crematorios, morgues o sitios de conservación de cadáveres, lo podrán hacer entidades públicas y privadas, para lo cual se dará cumplimiento a las normas establecidas en esta Ley.

Previamente se verificará la ubicación y la infraestructura a emplearse y que no constituyan riesgo para la salud. Deberán contar con el estudio de impacto ambiental y la correspondiente licencia ambiental.

Los cementerios y criptas son los únicos sitios autorizados para la inhumación de cadáveres y deben cumplir las normas establecidas por la autoridad sanitaria nacional y la correspondiente municipalidad.

Art. 88.- Practicada la necropsia, el cadáver debe ser obligatoriamente tratado, inhumado o cremado.

Ningún cadáver podrá mantenerse insepulto o sin someterse a cremación por más de setenta y dos horas, excepto cuando medie orden judicial o no sean reconocidos o reclamados por sus familiares o derechohabientes, en cuyo caso debe garantizarse su mantenimiento en los sitios autorizados y en condiciones de conservación adecuadas que no comprometan la integridad del cadáver ni alteren las posibles evidencias.

Art. 89.- Los cadáveres no identificados o que no fueren reclamados en el plazo de treinta días posteriores a su fallecimiento, se entregarán a título de donación a las facultades de Ciencias Médicas o de la Salud legalmente establecidas dando preferencia a las estatales, o se inhumarán de conformidad con las disposiciones pertinentes.

De los cadáveres no identificados, previa a su donación o inhumación se extraerán muestras que permita la obtención del perfil genético de la persona. Esta información será registrada en un banco de datos de cadáveres no identificados.

Art. 90.- No se podrá proceder a la inhumación o cremación de un cadáver sin que se cuente con el certificado médico que confirme la defunción y establezca sus posibles causas, de acuerdo a su diagnóstico. Esta responsabilidad corresponde a los cementerios o crematorios según el caso.

Art. 91.- La exhumación para efectos legales podrá practicarse en cualquier tiempo por orden de autoridad competente.

Art. 92.- El traslado de cadáveres, dentro del país, en los casos y condiciones establecidos en el reglamento de esta Ley, así como su ingreso al territorio nacional requiere autorización de la autoridad sanitaria nacional, quien establecerá las normas de conservación y seguridad.

Art. 93.- Las necropsias deben ser realizadas bajo responsabilidad de médicos patólogos o forenses, excepto en las localidades donde estos profesionales no existan, en cuyo caso se realizarán de acuerdo con lo establecido en el Código de Procedimiento Penal, sin costo para los familiares o deudos en las instituciones públicas.

Art. 94.- Es obligatoria la necropsia cuando:

a) No se conozca la causa del fallecimiento;
b) Por muerte repentina;
c) El Ministerio Público lo disponga;
d) En casos de emergencia sanitaria;
e) Por razones de salud pública; y,
f) Por petición y consentimiento del representante legal o pariente más cercano hasta el cuarto grado de consanguinidad y segundo de afinidad.

LIBRO II
Salud y seguridad ambiental

Disposición común

Art. 95.- La autoridad sanitaria nacional en coordinación con el Ministerio de Ambiente, establecerá las normas básicas para la preservación del ambiente en materias relacionadas con la salud humana, las mismas que serán de cumplimiento obligatorio para todas las personas naturales, entidades públicas, privadas y comunitarias.

El Estado a través de los organismos competentes y el sector privado está obligado a proporcionar a la población, información adecuada y veraz respecto del impacto ambiental y sus consecuencias para la salud individual y colectiva.

TITULO UNICO

CAPITULO I
Del agua para consumo humano

Art. 96.- Declárase de prioridad nacional y de utilidad pública, el agua para consumo humano.

Es obligación del Estado, por medio de las municipalidades, proveer a la población de agua potable de calidad, apta para el consumo humano.

Toda persona natural o jurídica tiene la obligación de proteger los acuíferos, las fuentes y cuencas hidrográficas que sirvan para el abastecimiento de agua para consumo humano. Se prohíbe realizar actividades de cualquier tipo, que pongan en riesgo de contaminación las fuentes de captación de agua. La autoridad sanitaria nacional, en coordinación con otros organismos competentes, tomarán medidas para prevenir, controlar, mitigar, remediar y sancionar la contaminación de las fuentes de agua para consumo humano.

A fin de garantizar la calidad e inocuidad, todo abastecimiento de agua para consumo humano, queda sujeto a la vigilancia de la autoridad sanitaria nacional, a quien corresponde establecer las normas y reglamentos que permitan asegurar la protección de la salud humana.

CAPITULO II
De los desechos comunes, infecciosos, especiales y de las radiaciones ionizantes y no ionizantes

Art. 97.- La autoridad sanitaria nacional dictará las normas para el manejo de todo tipo de desechos y residuos que afecten la salud humana; normas que serán de cumplimiento obligatorio para las personas naturales y jurídicas.

Art. 98.- La autoridad sanitaria nacional, en coordinación con las entidades públicas o privadas, promoverá programas y campañas de información y educación para el manejo de desechos y residuos.

Art. 99.- La autoridad sanitaria nacional, en coordinación con los municipios del país, emitirá los reglamentos, normas y procedimientos técnicos de cumplimiento obligatorio para el manejo adecuado de los desechos infecciosos que generen los establecimientos de servicios de salud, públicos o privados, ambulatorio o de internación, veterinaria y estética.

Art. 100.- La recolección, transporte, tratamiento y disposición final de desechos es responsabilidad de los municipios que la realizarán de acuerdo con las leyes, reglamentos y ordenanzas que se dicten para el efecto, con observancia de las normas de bioseguridad y control determinadas por la autoridad sanitaria nacional. El Estado entregará los recursos necesarios para el cumplimiento de lo dispuesto en este artículo.

Art. 101.- Las viviendas, establecimientos educativos, de salud y edificaciones en general, deben contar con sistemas sanitarios adecuados de disposición de excretas y evacuación de aguas servidas.

Los establecimientos educativos, públicos y privados, tendrán el número de baterías sanitarias que se disponga en la respectiva norma reglamentaria.

El Estado entregará a los establecimientos públicos los recursos necesarios para el cumplimiento de lo dispuesto en este artículo.

Art. 102.- Es responsabilidad del Estado, a través de los municipios del país y en coordinación con las respectivas instituciones públicas, dotar a la población de sistemas de alcantarillado sanitario, pluvial y otros de disposición de excretas y aguas servidas que no afecten a la salud individual,

colectiva y al ambiente; así como de sistemas de tratamiento de aguas servidas.

Art. 103.- Se prohíbe a toda persona, natural o jurídica, descargar o depositar aguas servidas y residuales, sin el tratamiento apropiado, conforme lo disponga en el reglamento correspondiente, en ríos, mares, canales, quebradas, lagunas, lagos y otros sitios similares. Se prohíbe también su uso en la cría de animales o actividades agropecuarias.

Los desechos infecciosos, especiales, tóxicos y peligrosos para la salud, deben ser tratados técnicamente previo a su eliminación y el depósito final se realizará en los sitios especiales establecidos para el efecto por los municipios del país.

Para la eliminación de desechos domésticos se cumplirán las disposiciones establecidas para el efecto.

Las autoridades de salud, en coordinación con los municipios, serán responsables de hacer cumplir estas disposiciones.

Art. 104.- Todo establecimiento industrial, comercial o de servicios, tiene la obligación de instalar sistemas de tratamiento de aguas contaminadas y de residuos tóxicos que se produzcan por efecto de sus actividades.

Las autoridades de salud, en coordinación con los municipios, serán responsables de hacer cumplir esta disposición.

Art. 105.- Las personas naturales o jurídicas propietarias de instalaciones o edificaciones, públicas o privadas, ubicadas en las zonas costeras e insulares, utilizarán las redes de alcantarillado para eliminar las aguas servidas y residuales producto de las actividades que desarrollen; y, en los casos que inevitablemente requieran eliminarlos en el mar, deberán tratarlos previamente, debiendo contar para el efecto con estudios de impacto ambiental; así como utilizar emisarios submarinos que cumplan con las normas sanitarias y ambientales correspondientes.

Art. 106.- Los terrenos por donde pasen o deban pasar redes de alcantarillado, acueductos o tuberías, se constituirán obligatoriamente en predios sirvientes, de acuerdo a lo establecido por la ley.

Las autoridades de salud, en coordinación con los municipios, serán responsables de hacer cumplir esta disposición.

Concordancias:

CODIGO CIVIL (LIBRO II), Arts. 868, 903

Art. 107.- La autoridad sanitaria nacional en coordinación con otros organismos competentes, dictará las normas para el manejo, transporte, tratamiento y disposición final de los desechos especiales. Los desechos radioactivos serán tratados de acuerdo con las normas dictadas por el organismo competente en la materia o aceptadas mediante convenios internacionales.

Art. 108.- Corresponde a la autoridad sanitaria nacional, en coordinación con la Comisión Ecuatoriana de Energía Atómica y más organismos competentes, vigilar el cumplimiento de las normas establecidas en materia de radiaciones ionizantes y no ionizantes.

Art. 109.- Ninguna persona será sometida o expuesta a radiaciones ionizantes y no ionizantes más allá de las dosis o límites permisibles, conforme a las normas pertinentes.

Los equipos diagnósticos y terapéuticos que utilicen radiaciones ionizantes y no ionizantes se instalarán en edificaciones técnicamente apropiadas y que cumplan con requisitos sanitarios y de

seguridad, establecidos por la autoridad sanitaria nacional y la Comisión Ecuatoriana de Energía Atómica; estarán sujetos a mantenimientos rigurosos y periódicos, debiendo contar con los certificados de control de calidad.

Art. 110.- Los importadores de artículos y dispositivos electrónicos que emiten radiaciones no ionizantes, deberán asegurarse que los mismos cumplan con las normas sanitarias vigentes, no estén prohibidos en su país de origen o en otros países; y, lleven la rotulación de precauciones e indicaciones claras sobre su uso.

CAPITULO III
Calidad del aire y de la contaminación acústica

Art. 111.- La autoridad sanitaria nacional, en coordinación con la autoridad ambiental nacional y otros organismos competentes, dictará las normas técnicas para prevenir y controlar todo tipo de emanaciones que afecten a los sistemas respiratorio, auditivo y visual.

Todas las personas naturales y jurídicas deberán cumplir en forma obligatoria dichas normas.

Art. 112.- Los municipios desarrollarán programas y actividades de monitoreo de la calidad del aire, para prevenir su contaminación por emisiones provenientes de fuentes fijas, móviles y de fenómenos naturales. Los resultados del monitoreo serán reportados periódicamente a las autoridades competentes a fin de implementar sistemas de información y prevención dirigidos a la comunidad.

Art. 113.- Toda actividad laboral, productiva, industrial, comercial, recreativa y de diversión; así como las viviendas y otras instalaciones y medios de transporte, deben cumplir con lo dispuesto en las respectivas normas y reglamentos sobre prevención y control, a fin de evitar la contaminación por ruido, que afecte a la salud humana.

CAPITULO IV
Plaguicidas y otras sustancias químicas

Art. 114.- La autoridad sanitaria nacional, en coordinación con el Ministerio de Agricultura y Ganadería y más organismos competentes, dictará e implementará las normas de regulación para la utilización y control de plaguicidas, fungicidas y otras sustancias químicas de uso doméstico, agrícola e industrial, que afecten a la salud humana.

Art. 115.- Se deben cumplir las normas y regulaciones nacionales e internacionales para la producción, importación, exportación, comercialización, uso y manipulación de plaguicidas, fungicidas y otro tipo de sustancias químicas cuya inhalación, ingestión o contacto pueda causar daño a la salud de las personas.

Art. 116.- Se prohíbe la producción, importación, comercialización y uso de plaguicidas, fungicidas y otras sustancias químicas, vetadas por las normas sanitarias nacionales e internacionales, así como su aceptación y uso en calidad de donaciones.

CAPITULO V
Salud y seguridad en el trabajo

Art. 117.- La autoridad sanitaria nacional, en coordinación con el Ministerio de Trabajo y Empleo y el Instituto Ecuatoriano de Seguridad Social, establecerá las normas de salud y seguridad en el trabajo para proteger la salud de los trabajadores.

Concordancias:

CODIGO DEL TRABAJO, Arts. 410

Art. 118.- Los empleadores protegerán la salud de sus trabajadores, dotándoles de información suficiente, equipos de protección, vestimenta apropiada, ambientes seguros de trabajo, a fin de prevenir, disminuir o eliminar los riesgos, accidentes y aparición de enfermedades laborales.

Concordancias:

CODIGO DEL TRABAJO, Arts. 42

Art. 119.- Los empleadores tienen la obligación de notificar a las autoridades competentes, los accidentes de trabajo y enfermedades laborales, sin perjuicio de las acciones que adopten tanto el Ministerio del Trabajo y Empleo como el Instituto Ecuatoriano de Seguridad Social.

Concordancias:

CODIGO DEL TRABAJO, Arts. 348, 386

Art. 120.- La autoridad sanitaria nacional, en coordinación con el Ministerio del Trabajo y Empleo y el Instituto Ecuatoriano de Seguridad Social, vigilará y controlará las condiciones de trabajo, de manera que no resulten nocivas o insalubres durante los períodos de embarazo y lactancia de las mujeres trabajadoras.

Los empleadores tienen la obligación de cumplir las normas y adecuar las actividades laborales de las mujeres embarazadas y en período de lactancia.

Concordancias:

CODIGO DE LA NIÑEZ Y ADOLESCENCIA, Arts. 30

CODIGO DEL TRABAJO, Arts. 138, 155

Art. 121.- Las instituciones públicas o privadas cuyo personal esté expuesto a radiación ionizante y emisiones no ionizantes, están obligadas a proveer de dispositivos de cuidado y control de radiación y de condiciones de seguridad en el trabajo que prevengan riesgos para la salud.

El incumplimiento de esta disposición por parte de los empleadores, que ocasione daño a la salud del trabajador, dará lugar a la aplicación de la sanción determinada por la ley.

Concordancias:

CODIGO DEL TRABAJO, Arts. 42, 410

CAPITULO VI
Del control de la fauna nociva y las zooantroposis

Art. 122.- La autoridad sanitaria nacional organizará campañas para erradicar la proliferación de vectores y otros animales que representen riesgo para la salud individual y colectiva.

Las personas naturales y jurídicas colaborarán con estas campañas.

Art. 123.- Es obligación de los propietarios de animales domésticos vacunarlos contra la rabia y otras enfermedades que la autoridad sanitaria nacional declare susceptibles de causar epidemias, así como mantenerlos en condiciones que no constituyan riesgo para la salud humana y la higiene del entorno.

El control y manejo de los animales callejeros es responsabilidad de los municipios, en coordinación con las autoridades de salud.

Art. 124.- Se prohíbe dentro del perímetro urbano instalar establos o granjas para criar o albergar ganado vacuno, equino, bovino, caprino, porcino, así como aves de corral y otras especies.

Art. 125.- Se prohíbe el faenamiento, transporte, industrialización y comercialización de animales muertos o sacrificados que hubieren padecido enfermedades nocivas para la salud humana.

Art. 126.- El ingreso de animales al país está sujeto al cumplimiento de las disposiciones legales y normativas emitidas por las autoridades correspondientes, los convenios internacionales y otras leyes que regulen el tráfico de animales.

Se prohíbe la entrada al país de animales afectados por enfermedades transmisibles a la población o sospechosos de estarlo, o que sean portadores de agentes patógenos cuya diseminación pueda constituir peligro para la salud de las personas.

Art. 127.- Toda persona procederá al exterminio de artrópodos, roedores y otras especies nocivas para la salud que existan en su vivienda, otros inmuebles y anexos de su propiedad o de su uso.

Será, además, obligación de la autoridad sanitaria nacional, impulsar campañas masivas para hacer efectivo el cumplimiento de este propósito.

Art. 128.- Las empresas que se dediquen al exterminio o control de plagas y vectores transmisores de enfermedades como dengue, rabia y paludismo, deberán obtener el respectivo permiso emitido por la autoridad sanitaria nacional para operar. Todos los químicos usados por dichas empresas deberán ser aprobados por dicha autoridad.

LIBRO III
Vigilancia y control sanitario

Disposiciones comunes

Art. 129.- El cumplimiento de las normas de vigilancia y control sanitario es obligatorio para todas las instituciones, organismos y establecimientos públicos y privados que realicen actividades de producción, importación, exportación, almacenamiento, transporte, distribución, comercialización y expendio de productos de uso y consumo humano.

La observancia de las normas de vigilancia y control sanitario se aplican también a los servicios de salud públicos y privados, con y sin fines de lucro, autónomos, comunitarios y de las empresas privadas de salud y medicina prepagada.

Art. 130.- Los establecimientos sujetos a control sanitario para su funcionamiento deberán contar con el permiso otorgado por la autoridad sanitaria nacional. El permiso de funcionamiento tendrá vigencia de un año calendario.

Art. 131.- El cumplimiento de las normas de buenas prácticas de manufactura, almacenamiento, distribución, dispensación y farmacia, será controlado y certificado por la autoridad sanitaria nacional.

Art. 132.- Las actividades de vigilancia y control sanitario incluyen las de control de calidad, inocuidad y seguridad de los productos procesados de uso y consumo humano, así como la verificación del cumplimiento de los requisitos técnicos y sanitarios en los establecimientos dedicados a la producción, almacenamiento, distribución, comercialización, importación y exportación de los productos señalados.

Art. 133.- La autoridad sanitaria nacional podrá delegar a los municipios, dentro de sus funciones, el ejercicio de las acciones necesarias para el control sanitario, quienes las realizarán de acuerdo con las disposiciones y normas emitidas por dicha autoridad.

Art. 134.- La instalación, transformación, ampliación y traslado de plantas industriales, procesadoras de alimentos, establecimientos farmacéuticos, de producción de biológicos, de elaboración de productos naturales procesados de uso medicinal, de producción de homeopáticos, plaguicidas, productos dentales, empresas de cosméticos y productos higiénicos, están sujetos a la obtención, previa a su uso, del permiso otorgado por la autoridad sanitaria nacional.

Art. 135.- Compete al organismo correspondiente de la autoridad sanitaria nacional autorizar la importación de todo producto inscrito en el registro sanitario, incluyendo muestras médicas y aquellos destinados a consumo interno procedentes de zonas francas.

No se autorizará la importación de productos, ni aún con fines promocionales, si previamente no tienen el registro sanitario nacional, salvo las excepciones determinadas en esta Ley.

Exceptúense de esta disposición, los productos sujetos al procedimiento de homologación, de acuerdo a la norma que expida la autoridad competente.

Nota: Artículo reformado por Disposición reformatoria Séptima, numeral 1 de Ley No. 0, publicada en Registro Oficial Suplemento 652 de 18 de diciembre del 2015 .

Art. 136.- Las materias primas para elaboración de productos sujetos a registro sanitario, no requieren para su importación cumplir con este registro, siempre que justifiquen su utilización en dichos productos.

TITULO UNICO

CAPITULO I

De las Autorizaciones

Nota: Capítulo con sus respectivos artículos sustituido por Disposición reformatoria Séptima, numeral 2 de Ley No. 0, publicada en Registro Oficial Suplemento 652 de 18 de diciembre del 2015 .

Art. 137.- Están sujetos a la obtención de notificación sanitaria previamente a su comercialización, los alimentos procesados, aditivos alimentarios, cosméticos, productos higiénicos, productos nutracéuticos, productos homeopáticos, plaguicidas para uso doméstico e industrial, y otros productos de uso y consumo humano definidos por la Autoridad Sanitaria Nacional, fabricados en el territorio nacional o en el exterior, para su importación, comercialización y expendio.

Están sujetos a la obtención de registro sanitario los medicamentos en general en la forma prevista en esta Ley, productos biológicos, productos naturales procesados de uso medicinal, productos dentales, dispositivos médicos y reactivos bioquímicos de diagnóstico, fabricados en el territorio nacional o en el exterior, para su importación, comercialización, dispensación y expendio.

Las donaciones de productos señalados en los incisos anteriores, se someterán a los requisitos establecidos en el reglamento que para el efecto dicte la autoridad competente.

Art. 138.- La Autoridad Sanitaria Nacional, a través de su entidad competente otorgará, suspenderá, cancelará o reinscribirá, la notificación sanitaria o el registro sanitario correspondiente, previo el cumplimiento de los trámites requisitos y plazos señalados en esta Ley y sus reglamentos, de acuerdo a las directrices y normas emitidas por la entidad competente de la autoridad sanitaria nacional, la cual fijará el pago de un importe para la inscripción y reinscripción de dicha notificación o registro sanitario.

Cuando se hubiere otorgado certificado de buenas prácticas o uno rigurosamente superior, no será exigible, notificación o registro sanitario, según corresponda, ni permiso de funcionamiento, excepto cuando se trate de aquellos productos señalados en el inciso segundo del artículo anterior.

La Autoridad Sanitaria Nacional ejercerá control administrativo, técnico y financiero de la entidad competente, referida en el primer inciso de este artículo, y monitoreará anualmente los resultados de la gestión para los fines pertinentes.

El informe técnico para el otorgamiento del registro o notificación sanitaria, según corresponda, deberá ser elaborado por la entidad competente de la autoridad sanitaria nacional.

Los análisis de calidad del control posterior, deberán ser elaborados por la autoridad competente de la autoridad sanitaria nacional, y por laboratorios, universidades y escuelas politécnicas, previamente acreditados por el organismo competente, de conformidad con la normativa aplicable, procedimientos que están sujetos al pago del importe establecido por la entidad competente de la autoridad sanitaria nacional.

Nota: Artículo sustituido por Disposición reformatoria Séptima, numeral 2 de Ley No. 0, publicada en Registro Oficial Suplemento 652 de 18 de diciembre del 2015 .

Art. 139.- Las notificaciones y registros sanitarios tendrán una vigencia mínima de cinco años, contados a partir de la fecha de su concesión, de acuerdo a lo previsto en la norma que dicte la autoridad sanitaria nacional. Todo cambio de la condición del producto que fue aprobado en la notificación o registro sanitario debe ser reportado obligatoriamente a la entidad competente de la autoridad sanitaria nacional.

Para el trámite de notificación o registro sanitario no se considerará como requisito la patente de los productos.

El registro sanitario de medicamentos no da derecho de exclusividad en el uso de la fórmula.

Nota: Artículo sustituido por Disposición reformatoria Séptima, numeral 2 de Ley No. 0, publicada en Registro Oficial Suplemento 652 de 18 de diciembre del 2015 .

Art. 140.- Queda prohibida la importación, comercialización y expendio de productos procesados para el uso y consumo humano que no cumplan con la obtención previa de la notificación o registro sanitario, según corresponda, salvo las excepciones previstas en esta Ley.

Nota: Artículo sustituido por Disposición reformatoria Séptima, numeral 2 de Ley No. 0, publicada en Registro Oficial Suplemento 652 de 18 de diciembre del 2015 .

Art. 141.- La notificación o registro sanitario correspondientes y el certificado de buenas prácticas o el rigurosamente superior, serán suspendidos o cancelados por la autoridad sanitaria nacional a través de la entidad competente, en cualquier tiempo si se comprobase que el producto o su fabricante no cumplen con los requisitos y condiciones establecidos en esta Ley y sus reglamentos, o cuando el producto pudiere provocar perjuicio a la salud, y se aplicarán las demás sanciones señaladas en esta Ley. Cuando se trate de certificados de buenas prácticas o rigurosamente superiores, además, se dispondrá la inmovilización de los bienes y productos.

En todos los casos, el titular de la notificación, registro sanitario, certificado de buenas prácticas o las personas naturales o jurídicas responsables, deberá resarcir plenamente cualquier daño que se produjere a terceros, sin perjuicio de otras acciones legales a las que hubiere lugar.

Nota: Artículo sustituido por Disposición reformatoria Séptima, numeral 2 de Ley No. 0, publicada en Registro Oficial Suplemento 652 de 18 de diciembre del 2015 .

Art. 142.- La entidad competente de la autoridad sanitaria nacional realizará periódicamente inspecciones a los establecimientos y controles posregistro de todos los productos sujetos a notificación o registro sanitario, a fin de verificar que se mantengan las condiciones que permitieron

su otorgamiento, mediante toma de muestras para análisis de control de calidad e inocuidad, sea en los lugares de fabricación, almacenamiento, transporte, distribución o expendio.

Si se detectare que algún establecimiento usa un número de notificación o registro no asignado para el producto, o distinto al que corresponda, la entidad competente de la autoridad sanitaria nacional suspenderá la comercialización de los productos, sin perjuicio de las sanciones de ley.

Nota: Artículo sustituido por Disposición reformatoria Séptima, numeral 2 de Ley No. 0, publicada en Registro Oficial Suplemento 652 de 18 de diciembre del 2015 .

Art. 143.- La publicidad y promoción de los productos sujetos a control y vigilancia sanitaria deberán ajustarse a su verdadera naturaleza, composición, calidad u origen, de modo tal que se evite toda concepción errónea de sus cualidades o beneficios, lo cual será controlado por la autoridad sanitaria nacional.

Se prohibe la publicidad por cualquier medio de medicamentos sujetos a venta bajo prescripción.

Nota: Artículo sustituido por Disposición reformatoria Séptima, numeral 2 de Ley No. 0, publicada en Registro Oficial Suplemento 652 de 18 de diciembre del 2015 .

Art. 144.- La autoridad sanitaria nacional, a través de la entidad competente podrá autorizar la importación de medicamentos, productos biológicos, dispositivos médicos, reactivos bioquímicos y de diagnóstico que no hayan obtenido el correspondiente registro sanitario, en casos de emergencia sanitaria, para personas que requieren tratamientos especializados no disponibles en el país, para personas que sufran enfermedades catastróficas, raras o huérfanas, para fines de investigación clínica humana, para el abastecimiento del sector público a través de organismos internacionales, tratándose de donaciones aceptadas por la autoridad sanitaria nacional, o para otros casos definidos por la autoridad sanitaria nacional, y en otros casos previstos en esta Ley, previo el cumplimiento de los requisitos establecidos para el efecto. Los medicamentos, productos biológicos, dispositivos médicos, reactivos bioquímicos y de diagnóstico cuya importación se permita, serán los específicos para cada situación.

Nota: Artículo reformado por Ley No. 0, publicada en Registro Oficial 625 de 24 de Enero del 2012 .
Nota: Artículo sustituido por Disposición reformatoria Séptima, numeral 2 de Ley No. 0, publicada en Registro Oficial Suplemento 652 de 18 de diciembre del 2015 .

CAPITULO II
De los alimentos

Art. 145.- Es responsabilidad de los productores, expendedores y demás agentes que intervienen durante el ciclo producción consumo, cumplir con las normas establecidas en esta Ley y demás disposiciones vigentes para asegurar la calidad e inocuidad de los alimentos para consumo humano.

Art. 146.- En materia de alimentos se prohíbe:

a) El uso de aditivos para disimular, atenuar o corregir las deficiencias tecnológicas de producción, manipulación o conservación y para resaltar fraudulentamente sus características;
b) La utilización, importación y comercialización de materias primas no aptas para consumo humano;
c) La inclusión de substancias nocivas que los vuelvan peligrosos o potencialmente perjudiciales para la salud de los consumidores;
d) El uso de materias primas y productos tratados con radiaciones ionizantes o que hayan sido genéticamente modificados en la elaboración de fórmulas para lactantes y alimentos infantiles;
e) El procesamiento y manipulación en condiciones no higiénicas;
f) La utilización de envases que no cumplan con las especificaciones técnicas aprobadas para el efecto;
g) La oferta de un alimento procesado con nombres, marcas, gráficos o etiquetas que hagan

aseveraciones falsas o que omitan datos de manera que se confunda o lleve a error al consumidor;
h) El almacenamiento de materias primas o alimentos procesados en locales en los que se encuentren substancias nocivas o peligrosas;
i) Cualquier forma de falsificación, contaminación, alteración o adulteración, o cualquier procedimiento que produzca el efecto de volverlos nocivos o peligrosos para la salud humana; y,
j) La exhibición y venta de productos cuyo período de vida útil haya expirado.

Art. 147.- La autoridad sanitaria nacional, en coordinación con los municipios, establecerá programas de educación sanitaria para productores, manipuladores y consumidores de alimentos, fomentando la higiene, la salud individual y colectiva y la protección del medio ambiente.

Concordancias:

CODIGO DE LA NIÑEZ Y ADOLESCENCIA, Arts. 28

Art. 148.- El control del expendio de alimentos y bebidas en la vía pública lo realizarán los municipios, en coordinación con la autoridad sanitaria nacional y de conformidad con lo establecido en la Ley Orgánica de Régimen Municipal.

Art. 149.- El desarrollo, tratamiento, elaboración, producción, aplicación, manipulación, uso, almacenamiento, transporte, distribución, importación, comercialización y expendio de alimentos para consumo humano que sean o contengan productos genéticamente modificados, se realizará cuando se demuestre ante la autoridad competente, mediante estudios técnicos y científicamente avanzados, su inocuidad y seguridad para los consumidores y el medio ambiente.

Para cumplir con este propósito, la autoridad sanitaria nacional deberá coordinar con los organismos técnicos públicos y privados correspondientes.

Art. 150.- La donación de alimentos que contengan productos genéticamente modificados, así como su utilización, uso y manejo en planes y programas y planes de ayuda alimentaria, serán aceptados si es que mediante procedimientos técnicos y científicamente avanzados, demuestren su inocuidad y seguridad ante la autoridad sanitaria nacional.

Para cumplir con este propósito, la autoridad sanitaria nacional actuará de conformidad con los principios universales en materia de salud pública y lo establecido en el inciso segundo del artículo precedente.

Art. 151.- Los envases de los productos que contengan alimentos genéticamente modificados, sean nacionales o importados, deben incluir obligatoriamente, en forma visible y comprensible en sus etiquetas, el señalamiento de esta condición, además de los otros requisitos que establezca la autoridad sanitaria nacional, de conformidad con la ley y las normas reglamentarias que se dicten para el efecto.

Art. 152.- La autoridad sanitaria nacional, en coordinación con los organismos competentes, establecerá e implementará un sistema nacional integrado para garantizar la inocuidad de los alimentos.

CAPITULO III
De los medicamentos

Art. 153.- Todo medicamento debe ser comercializado en establecimientos legalmente autorizados.

Para la venta al público se requiere de receta emitida por profesionales facultados para hacerlo, a excepción de los medicamentos de venta libre, clasificados como tales con estricto apego a normas farmacológicas actualizadas, a fin de garantizar la seguridad de su uso y consumo.

Art. 154.- El Estado garantizará el acceso y disponibilidad de medicamentos de calidad y su uso racional, priorizando los intereses de la salud pública sobre los económicos y comerciales.

Promoverá la producción, importación, comercialización, dispensación y expendio de medicamentos genéricos con énfasis en los esenciales, de conformidad con la normativa vigente en la materia. Su uso, prescripción, dispensación y expendio es obligatorio en las instituciones de salud pública.

Art. 155.- Los medicamentos en general, incluyendo los productos que contengan nuevas entidades químicas que obtengan registro sanitario nacional y no sean comercializados por el lapso de un año, serán objeto de cancelación de dicho registro sanitario.

Art. 156.- La autoridad sanitaria nacional autorizará la importación de medicamentos en general, en las cantidades necesarias para la obtención del requisito sanitario, de conformidad con lo previsto en el reglamento correspondiente.

Art. 157.- La autoridad sanitaria nacional garantizará la calidad de los medicamentos en general y desarrollará programas de fármaco vigilancia y estudios de utilización de medicamentos, entre otros, para precautelar la seguridad de su uso y consumo.

Además realizará periódicamente controles posregistro y estudios de utilización de medicamentos para evaluar y controlar los estándares de calidad, seguridad y eficacia y sancionar a quienes comercialicen productos que no cumplan dichos estándares, falsifiquen o adulteren los productos farmacéuticos.

Art. 158.- El desarrollo, la producción, manipulación, uso, almacenamiento, transporte, distribución, importación, comercialización y expendio de productos nutraceúticos, será permitido cuando se demuestre técnica y científicamente ante la autoridad sanitaria nacional, su seguridad para el consumidor y el ambiente. El registro y control sanitarios de estos productos se sujetará a las regulaciones vigentes para medicamentos.

Art. 159.- Corresponde a la autoridad sanitaria nacional la fijación, revisión y control de precios de los medicamentos de uso y consumo humano a través del Consejo Nacional de Fijación y Revisión de Precios de Medicamentos de Uso Humano, de conformidad con la ley.

Se prohíbe la comercialización de los productos arriba señalados sin fijación o revisión de precios.

Art. 160.- En ningún caso los gastos de promoción y publicidad se podrán considerar como parte de la estructura de costos para el análisis de fijación de precios.

Nota: Artículo sustituido por Ley No. 00, publicada en Registro Oficial Suplemento 555 de 13 de Octubre del 2011 .

Art. 161.- Para la fijación y revisión de precios de medicamentos importados, se considerará el precio en el puerto de embarque (FOB) del país de origen del producto, el mismo que no podrá ser superior a los precios de venta al distribuidor o mayorista del país de origen.

Art. 162.- Los precios de venta al público deben estar impresos en los envases de manera que no puedan ser removidos. Se prohíbe alterar los precios o colocar etiquetas que los modifiquen.

Art. 163.- Los laboratorios farmacéuticos, distribuidoras farmacéuticas, casas de representación de medicamentos, dispositivos médicos, productos dentales, reactivos bioquímicos y de diagnóstico, en las ventas que realicen a las instituciones públicas descontarán un porcentaje no inferior al 15% del precio de venta a farmacia.

CAPITULO IV
De los productos naturales procesados de uso medicinal

Art. 164.- Los productos naturales procesados de uso medicinal, se producirán, almacenarán, comercializarán e importarán siempre que cuenten con registro sanitario nacional, de conformidad con la ley y el reglamento correspondiente y bajo las normas de calidad emitidas por la autoridad sanitaria nacional a través de la entidad competente.

Nota: Artículo reformado por Disposición reformatoria Séptima, numeral 2 de Ley No. 0, publicada en Registro Oficial Suplemento 652 de 18 de diciembre del 2015 .

CAPITULO V
De los establecimientos farmacéuticos

Art. 165.- Para fines legales y reglamentarios, son establecimientos farmacéuticos los laboratorios farmacéuticos, casas de representación de medicamentos, distribuidoras farmacéuticas, farmacias y botiquines, que se encuentran en todo el territorio nacional.

Art. 166.- Las farmacias deben atender al público mínimo doce horas diarias, ininterrumpidas y cumplir obligatoriamente los turnos establecidos por la autoridad sanitaria nacional. Requieren obligatoriamente para su funcionamiento la dirección técnica y responsabilidad de un profesional químico farmacéutico o bioquímico farmacéutico, quien brindará atención farmacéutica especializada.

Los botiquines estarán a cargo de personas calificadas y certificadas para el manejo de medicamentos. La autorización para su funcionamiento es transitoria y revocable.

La autoridad sanitaria nacional implementará farmacias y botiquines institucionales, debidamente equipados, en todas sus unidades operativas de acuerdo al nivel de complejidad.

Art. 167.- La receta emitida por los profesionales de la salud facultados por ley para hacerlo, debe contener obligatoriamente y en primer lugar el nombre genérico del medicamento prescrito.

Quien venda informará obligatoriamente al comprador sobre la existencia del medicamento genérico y su precio.

No se aceptarán recetas ilegibles, alteradas o en clave.

Art. 168.- Son profesionales de la salud humana facultados para prescribir medicamentos, los médicos, odontólogos y obstetrices.

Art. 169.- La venta de medicamentos al público al por menor sólo puede realizarse en establecimientos autorizados para el efecto.

Art. 170.- Los medicamentos, para su venta deben cumplir con los siguientes requisitos:

a) Estar debidamente identificados y etiquetados, sin alteraciones ni enmiendas;
b) Contener en sus etiquetas el número de registro sanitario nacional, el precio de venta al público y la fecha de expiración;
c) No estar caducados;
d) No provenir de instituciones de servicio social, de programas sociales estatales, de donaciones o ser muestras médicas;
e) No haber sido introducidos clandestinamente al país;
f) No ser falsificados o adulterados; y,
g) No tener colocados elementos sobre las etiquetas que impidan la visibilidad de la información del producto, incluidas las que contienen los precios.

Art. 171.- Es prohibida la venta de medicamentos que contengan sustancias psicotrópicas y

estupefacientes que no cuenten con receta emitida por profesionales autorizados para prescribirlas. Cuando se requiera la prescripción y venta de medicamentos que contengan estas sustancias, se realizará conforme a las normas emitidas por la autoridad sanitaria nacional y la Ley de Sustancias Estupefacientes y Psicotrópicas.

Art. 172.- En las farmacias y botiquines no se podrá ofrecer o dar consulta médica, obstétrica, odontológica, aplicar tratamientos, realizar toma de muestras ni tener laboratorios clínicos.

Art. 173.- Todo establecimiento farmacéutico debe contar con la responsabilidad técnica de un profesional químico farmacéutico o bioquímico farmacéutico, quien puede tener bajo su responsabilidad técnica uno o más establecimientos farmacéuticos, de conformidad con lo que establezca el reglamento.

El reglamento de aplicación de esta Ley normará lo relacionado a este servicio, en los lugares en donde no existan suficientes profesionales ni establecimientos farmacéuticos.

Art. 174.- Se prohíbe a los expendedores de farmacias recomendar la utilización de medicamentos que requieran receta médica o cambiar la sustancia activa prescrita, sin la autorización escrita del prescriptor.

Art. 175.- Sesenta días antes de la fecha de caducidad de los medicamentos, las farmacias y botiquines notificarán a sus proveedores, quienes tienen la obligación de retirar dichos productos y canjearlos de acuerdo con lo que establezca la reglamentación correspondiente.

Art. 176.- Los medicamentos caducados referidos en el artículo anterior deben ser destruidos y eliminados por los fabricantes o importadores, conforme a los procedimientos establecidos por la autoridad sanitaria nacional y bajo su supervisión.

CAPITULO VI
Otros establecimientos sujetos a control sanitario

Art. 177.- Es responsabilidad de la autoridad sanitaria nacional, expedir normas y controlar las condiciones higiénico sanitarias de establecimientos de servicios de atención al público y otros sujetos a control sanitario, para el otorgamiento o renovación del permiso de funcionamiento.

En el caso de establecimientos educativos públicos y privados, vigilará, controlará y evaluará periódicamente la infraestructura y condiciones higiénico sanitarias requisitos necesarios para su funcionamiento.

Art. 178.- Los establecimientos de producción, almacenamiento, envase o expendio de productos naturales de uso medicinal y de medicamentos homeopáticos, requieren para su instalación y funcionamiento del permiso otorgado por la autoridad sanitaria nacional.

Art. 179.- Las casas de representación y distribuidoras de productos dentales, dispositivos médicos, reactivos bioquímicos y de diagnóstico, para su funcionamiento deberán obtener el permiso de la autoridad sanitaria nacional.

LIBRO IV
De los servicios y profesiones de salud

TITULO UNICO

CAPITULO I
De los servicios de salud

Art. 180.- La autoridad sanitaria nacional regulará, licenciará y controlará el funcionamiento de los

servicios de salud públicos y privados, con y sin fines de lucro, autónomos, comunitarios y de las empresas privadas de salud y medicina prepagada y otorgará su permiso de funcionamiento.

Regulará los procesos de licenciamiento y acreditación.

Regulará y controlará el cumplimiento de la normativa para la construcción, ampliación y funcionamiento de estos establecimientos de acuerdo a la tipología, basada en la capacidad resolutiva, niveles de atención y complejidad.

Art. 181.- La autoridad sanitaria nacional regulará y vigilará que los servicios de salud públicos y privados, con y sin fines de lucro, autónomos y las empresas privadas de salud y medicina prepagada, garanticen atención oportuna, eficiente y de calidad según los enfoques y principios definidos en esta Ley.

Art. 182.- La autoridad sanitaria nacional, regulará y aprobará las tarifas de los servicios de salud y las de los planes y programas de las empresas de servicios de salud y medicina prepagada, de conformidad con el reglamento que se emita para el efecto.

Art. 183.- El contrato de prestación de servicios de medicina prepagada debe ser aprobado por la autoridad sanitaria nacional.

Es obligación de las empresas de medicina prepagada obtener dicha aprobación y hacerla constar en el contrato respectivo.

Art. 184.- Es obligación de los servicios de salud exhibir en sitios visibles para el público, las tarifas que se cobran por sus servicios, las mismas que deben estar aprobadas por la autoridad sanitaria nacional.

Art. 185.- Los servicios de salud funcionarán, de conformidad con su ámbito de competencia, bajo la responsabilidad técnica de un profesional de la salud.

Art. 186.- Es obligación de todos los servicios de salud que tengan salas de emergencia, recibir y atender a los pacientes en estado de emergencia. Se prohíbe exigir al paciente o a las personas relacionadas un pago, compromiso económico o trámite administrativo, como condición previa a que la persona sea recibida, atendida y estabilizada en su salud.

Una vez que el paciente haya superado la emergencia, el establecimiento de salud privado podrá exigir el pago de los servicios que recibió.

Art. 187.- Los valores no recuperados por el servicio de salud por la atención a un paciente en estado de emergencia, cuya imposibilidad de pago esté debidamente comprobada, se deducirán del impuesto a la renta de conformidad con las disposiciones de la Ley de Régimen Tributario Interno.

Concordancias:

LEY ORGANICA DE REGIMEN TRIBUTARIO INTERNO, LORTI, Arts. 16

Art. 188.- La autoridad sanitaria nacional, regulará y vigilará que los servicios de salud públicos y privados apliquen las normas de prevención y control de infecciones nosocomiales.

CAPITULO II
De las medicinas tradicionales y alternativas

Art. 189.- Los integrantes del Sistema Nacional de Salud respetarán y promoverán el desarrollo de las medicinas tradicionales, incorporarán el enfoque intercultural en las políticas, planes, programas, proyectos y modelos de atención de salud, e integrarán los conocimientos de las medicinas

tradicionales y alternativas en los procesos de enseñanza - aprendizaje.

Art. 190.- La autoridad sanitaria nacional promoverá e impulsará el intercambio de conocimientos entre los distintos agentes de las medicinas tradicionales, fomentará procesos de investigación de sus recursos diagnósticos y terapéuticos en el marco de los principios establecidos en esta Ley, protegiendo los derechos colectivos de los pueblos indígenas y negros o afroecuatorianos.

Art. 191.- La autoridad sanitaria nacional implementará procesos de regulación y control, para evitar que las prácticas de las medicinas tradicionales atenten a la salud de las personas.

Art. 192.- Los integrantes del Sistema Nacional de Salud respetarán y promoverán el desarrollo de las medicinas alternativas en el marco de la atención integral de salud.

Las medicinas alternativas deben ser ejercidas por profesionales de la salud con títulos reconocidos y certificados por el CONESUP y registrados ante la autoridad sanitaria nacional.

Las terapias alternativas requieren para su ejercicio, el permiso emitido por la autoridad sanitaria nacional.

CAPITULO III
De las profesiones de salud, afines y su ejercicio

Art. 193.- Son profesiones de la salud aquellas cuya formación universitaria de tercer o cuarto nivel está dirigida específica y fundamentalmente a dotar a los profesionales de conocimientos, técnicas y prácticas, relacionadas con la salud individual y colectiva y al control de sus factores condicionantes.

Art. 194.- Para ejercer como profesional de salud, se requiere haber obtenido título universitario de tercer nivel, conferido por una de las universidades establecidas y reconocidas legalmente en el país, o por una del exterior, revalidado y refrendado. En uno y otro caso debe estar registrado ante el CONESUP y por la autoridad sanitaria nacional.

Art. 195.- Los títulos de nivel técnico superior o tecnológico así como los de auxiliares en distintas ramas de la salud, para su habilitación deben ser registrados en las instancias respectivas e inscritos ante la autoridad sanitaria nacional.

Art. 196.- La autoridad sanitaria nacional analizará los distintos aspectos relacionados con la formación de recursos humanos en salud, teniendo en cuenta las necesidades nacionales y locales, con la finalidad de promover entre las instituciones formadoras de recursos humanos en salud, reformas en los planes y programas de formación y capacitación.

Art. 197.- Para la habilitación del ejercicio profesional y el registro correspondiente, los profesionales de salud deben realizar un año de práctica en las parroquias rurales o urbano marginales, con remuneración, en concordancia con el modelo de atención y de conformidad con el reglamento correspondiente en los lugares destinados por la autoridad sanitaria nacional, al término del cual se le concederá la certificación que acredite el cumplimiento de la obligación que este artículo establece.

La autoridad sanitaria nacional en coordinación con organismos seccionales y organizaciones de base, controlará la asignación y el cumplimiento del año obligatorio por parte de los profesionales que cumplen el año de salud rural.

Se prohíbe el ejercicio de la práctica rural en unidades operativas urbanas de segundo y tercer nivel.

Art. 198.- Los profesionales y técnicos de nivel superior que ejerzan actividades relacionadas con la salud, están obligados a limitar sus acciones al área que el título les asigne.

Art. 199.- Corresponde a la autoridad sanitaria nacional la investigación y sanción de la práctica ilegal, negligencia, impericia, imprudencia e inobservancia en el ejercicio de las profesiones de la salud, sin perjuicio de la acción de la justicia ordinaria.

Art. 200.- El profesional que ampare con su título o con su firma el ejercicio de las profesiones de la salud a personas no autorizadas, sin perjuicio de lo establecido en esta Ley, será sancionado de acuerdo con la legislación aplicable.

Art. 201.- Es responsabilidad de los profesionales de salud, brindar atención de calidad, con calidez y eficacia, en el ámbito de sus competencias, buscando el mayor beneficio para la salud de sus pacientes y de la población, respetando los derechos humanos y los principios bioéticos.

Es su deber exigir condiciones básicas para el cumplimiento de lo señalado en el inciso precedente.

Art. 202.- Constituye infracción en el ejercicio de las profesiones de salud, todo acto individual e intransferible, no justificado, que genere daño en el paciente y sea resultado de:

a) Inobservancia, en el cumplimiento de las normas;
b) Impericia, en la actuación del profesional de la salud con falta total o parcial de conocimientos técnicos o experiencia;
c) Imprudencia, en la actuación del profesional de la salud con omisión del cuidado o diligencia exigible; y,
d) Negligencia, en la actuación del profesional de la salud con omisión o demora injustificada en su obligación profesional.

Nota: El artículo 17 del Código Orgánico Integral Penal dispone: "Se considerarán exclusivamente como infracciones penales las tipificadas en este Código. Las acciones u omisiones punibles, las penas o procedimientos penales previstos en otras normas jurídicas no tendrán validez jurídica alguna, salvo en materia de niñez y adolescencia.

Art. 203.- Los servicios de salud, serán corresponsables civilmente, de las actuaciones de los profesionales de la salud que laboran en ellos.

Art. 204.- El consentimiento o autorización del paciente o de la persona que le representa legalmente, no exime de responsabilidad al profesional o al servicio de salud en aquellos casos determinados en el artículo 202 de esta Ley.

CAPITULO IV
De la capacitación sanitaria

Art. 205.- Créase la carrera sanitaria para los recursos humanos del Sistema Nacional de Salud, basada en el criterio de clasificación por niveles de formación y estructura ocupacional, con el propósito de establecer sus obligaciones y derechos, así como los incentivos que permitan garantizar la equidad, calidad en la atención y el servicio, la asignación adecuada y suficiente de recursos humanos en las distintas zonas del país.

La autoridad sanitaria nacional promoverá y desarrollará, dentro de la carrera sanitaria, un plan nacional de educación permanente con enfoque de género y pluricultural, para mejorar la productividad, calidad del desempeño laboral y promoción de sus recursos humanos.

Art. 206.- La autoridad sanitaria nacional establecerá planes de capacitación y evaluación permanente de los profesionales y recursos humanos en salud e implementará promociones e incentivos.

LIBRO V
TITULO UNICO

Investigación científica en salud, genética y sistema de información en salud

CAPITULO I
De la investigación científica en salud

Art. 207.- La investigación científica en salud así como el uso y desarrollo de la biotecnología, se realizará orientada a las prioridades y necesidades nacionales, con sujeción a principios bioéticos, con enfoques pluricultural, de derechos y de género, incorporando las medicinas tradicionales y alternativas.

Art. 208.- La investigación científica tecnológica en salud será regulada y controlada por la autoridad sanitaria nacional, en coordinación con los organismos competentes, con sujeción a principios bioéticos y de derechos, previo consentimiento informado y por escrito, respetando la confidencialidad.

CAPITULO II
De la genética humana

Art. 209.- La autoridad sanitaria nacional normará, licenciará y controlará el funcionamiento de los servicios de salud especializados, públicos y privados, para el ejercicio de actividades relacionadas con la investigación y desarrollo de la genética humana. Igualmente controlará el ejercicio profesional de quienes realicen dichas actividades, que deberán necesariamente tener especialidad en el área de genética o afines.

Art. 210.- Sólo podrán hacerse pruebas de identificación humana, filiación y compatibilidad de antígenos para:

a) Trasplantes;
b) Estudios mutacionales;
c) Ligamiento genético;
d) Pruebas predictivas de enfermedades genéticas.
e) Pruebas para detectar la predisposición genética a una enfermedad;
f) Fines terapéuticos; y,
g) Otras que se desarrollen con fines de salud genética.

Estas pruebas deberán contar con asesoramiento y supervisión genético especializado, siguiendo procedimientos científicamente probados, con sujeción y respeto a los principios bioéticos.

Art. 211.- Se prohíbe toda forma de discriminación de una persona a causa de su patrimonio genético.

Es obligatorio guardar confidencialidad respecto al genoma individual de la persona.

Art. 212.- Se prohíbe la intervención genética sobre células de la línea germinal y células madre, con fines de experimentación y lucro.

Podrán efectuarse intervenciones sobre el genoma humano, células de la línea germinal y células madre únicamente por razones predictivas, preventivas, diagnósticas o terapéuticas, siempre que se disponga de asesoramiento genético especializado, procedimientos científicamente probados y seguros, previo consentimiento informado, expreso y escrito de la persona y que sea de beneficio social y eugenésico.

Art. 213.- No se podrán patentar genes ni derivados celulares humanos naturales.

Art. 214.- Se prohíben las prácticas de clonación de seres humanos, así como la obtención de

embriones humanos con fines de experimentación.

La autoridad sanitaria nacional procurará y fomentará la integración y trabajo cooperativo de los centros de investigación y desarrollo de la genética.

CAPITULO III
Del sistema común de información

Art. 215.- La autoridad sanitaria nacional con la participación de los integrantes del Sistema Nacional de Salud, implementará el sistema común de información con el fin de conocer la situación de salud, identificar los riesgos para las personas y el ambiente, dimensionar los recursos disponibles y la producción de los servicios, para orientar las decisiones políticas y gerenciales y articular la participación ciudadana en todos los niveles, entre otras.

Este sistema incorporará los enfoques pluricultural, multiétnico, de género, las particularidades regionales y poblacionales, así como la división político - administrativa del país.

LIBRO SEXTO
Jurisdicción, competencia, procedimiento, sanciones y definiciones

CAPITULO I
De la jurisdicción y competencia

Art. 216.- La jurisdicción y competencia administrativa, en materia de salud nacen de esta Ley.

Art. 217.- Tienen jurisdicción para conocer, juzgar e imponer las sanciones previstas en esta Ley y demás normas vigentes, las siguientes autoridades de salud:

a) El Ministro de Salud Pública;
b) El Director General de Salud;
c) Los directores provinciales de salud; y,
d) Los comisarios de salud.

Art. 218.- Los comisarios de salud deben ser doctores en jurisprudencia o abogados con experiencia mínima de tres años de ejercicio profesional.

Art. 219.- El Ministro de Salud Pública y el Director General de Salud, tienen competencia en todo el territorio nacional.

Los directores provinciales de salud y los comisarios de salud, tienen competencia en el ámbito provincial.

Art. 220.- La Fuerza Pública está obligada a colaborar con las autoridades de salud para hacer cumplir las disposiciones de esta Ley, cuando se requiera su intervención.

CAPITULO II
Del procedimiento

Art. 221.- Las autoridades de salud señaladas en el Capítulo anterior, actuarán de oficio, por denuncia o informe para conocer y sancionar las infracciones señaladas en esta Ley. Las denuncias se presentarán en forma verbal o por escrito.

Art. 222.- Las autoridades de salud señaladas en el artículo 217, que no cumplieren adecuadamente su obligación de conocer, juzgar e imponer las sanciones previstas en esta Ley, serán sancionadas de conformidad con el reglamento correspondiente y demás normas atinentes a la materia, sin perjuicio de las acciones administrativas, civiles y penales a que hubiere lugar.

Art. 223.- Se concede acción pública para denunciar cualquier infracción a las disposiciones de la presente Ley.

Art. 224.- Cuando se actúe de oficio o mediante informe o denuncia, la autoridad de salud correspondiente dictará un auto inicial que contendrá:

a) La relación sucinta de los hechos y del modo como llegaron a su conocimiento;
b) La orden de citar al presunto infractor, disponiendo que señale domicilio para entregar las notificaciones, bajo prevención de que será juzgado en rebeldía en caso de no comparecer;
c) La orden de agregar al expediente el informe o denuncia, si existieren, y de que se practiquen las diligencias que sean necesarias para comprobar la infracción;
d) El señalamiento del día y hora para que tenga lugar la audiencia de juzgamiento; y,
e) La designación del secretario que actuará en el proceso.

Art. 225.- Corresponde conocer y resolver las causas en primera instancia:

a) Al comisario de salud, las infracciones sancionadas en los artículos 241, 242, 243, 244 y 245 de esta Ley;
b) Al director provincial de salud, las infracciones sancionadas en los artículos 246, 247, 248 y 256 de esta Ley; y,
c) Al Director General de Salud, las infracciones sancionadas en los artículos 249, 250, 251, 252, 254 y 255 de esta Ley.

De no ser competente la autoridad se inhibirá de conocer la causa y la remitirá de oficio, a quien corresponda.

Art. 226.- En caso de que la infracción tenga indicios de responsabilidad penal, el expediente se remitirá a la autoridad competente.

Art. 227.- La citación con el auto inicial, se hará personalmente al infractor, en su domicilio o lugar de trabajo; si no se le encontrare, se le citará mediante tres boletas dejadas en el domicilio o lugar de trabajo, en diferentes días, sentando la razón de la citación.

Art. 228.- En la audiencia de juzgamiento, se oirá al infractor, que intervendrá por sí o por medio de su abogado; se recibirán las pruebas que presente y se agregarán al proceso, de lo cual se dejará constancia en acta firmada por el compareciente, la autoridad de salud correspondiente y el secretario.

Art. 229.- De solicitarlo cualquiera de las partes o de oficio, en la misma diligencia, se abrirá la causa a prueba por el término de seis días, en la cual se practicarán todas las pruebas que se soliciten.

Art. 230.- De no haberse solicitado que se abra la causa a prueba, la autoridad de salud correspondiente procederá a dictar la resolución en el término de cinco días.

Art. 231.- Vencido el término de prueba y practicadas todas las diligencias oportunamente solicitadas y ordenadas, la autoridad de salud correspondiente dictará su resolución dentro del termino de cinco días.

Art. 232.- De las resoluciones del comisario de salud, podrá apelarse ante el director provincial de salud; de las que dicte el director provincial de salud, ante el Director General de Salud; y, de las de esta autoridad ante el Ministro de Salud Pública, siendo estas decisiones de segunda y definitiva instancia.

Las resoluciones podrán ser apeladas dentro del término de tres días luego de ser notificadas a las partes; la autoridad superior dentro del término de ocho días desde que avoca conocimiento deberá

dictar la correspondiente resolución.

Unicamente podrán apelarse las resoluciones de primera instancia, las de segunda instancia causarán ejecutoria.

Art. 233.- Una vez que la resolución esté ejecutoriada, se emitirá la orden de pago, la misma que de no ser pagada por el sujeto pasivo, será cobrada por la vía coactiva por el Ministerio de Salud Pública de acuerdo a lo establecido en el artículo 941 del Código de Procedimiento Civil, en el cual se le concede al Estado y a sus instituciones, acción y jurisdicción coactiva a nivel nacional.

Art. 234.- Los bienes y productos que fueren comisados y puedan ser utilizados, se entregarán a las instituciones de beneficencia de la jurisdicción provincial en donde se haya cometido la infracción, de conformidad con el reglamento que al efecto emita la autoridad sanitaria nacional.

Art. 235.- Los bienes y productos que no puedan ser utilizados, serán destruidos por la autoridad correspondiente, dejando constancia en acta que suscribirá conjuntamente con el secretario, cuyo original se remitirá a la autoridad inmediata superior para su conocimiento.

Art. 236.- En todo lo no previsto en esta Ley, se actuará de conformidad con lo previsto en los Códigos Penal y Civil; y, de Procedimiento Penal y Civil.

CAPITULO III
De las sanciones

Art. 237.- Las infracciones en materia de salud serán sancionadas de conformidad con las disposiciones contenidas en esta Ley y sus reglamentos, sin perjuicio de las sanciones civiles, administrativas y penales a que hubiera lugar.

Art. 238.- En la concurrencia de más de una infracción a las disposiciones de esta Ley y sus reglamentos, la persona será juzgada por todas las cometidas.

Art. 239.- La reincidencia en el incumplimiento de esta Ley y sus reglamentos, será reprimida con el doble del máximo de la sanción para cada caso, sin perjuicio de las sanciones civiles o penales a que hubiere lugar.

Art. 240.- Las infracciones determinadas en esta ley se sancionarán con:

a) Multa;
b) Suspensión del permiso o licencia;
c) Suspensión del ejercicio profesional;
d) Decomiso; y,
e) Clausura parcial, temporal o definitiva del establecimiento correspondiente.

CAPITULO IV
De las infracciones

Art. 241.- Será sancionado con multa de un salario básico unificado del trabajador en general, el incumplimiento a lo dispuesto en los artículos 32, 53, 61, 64, 74 inciso segundo, 101, 111 inciso segundo, 115, 120 inciso segundo, 122, 123, 167, 195, 198 y 202 literal a), de esta Ley.

Art. 242.- Será sancionado con multa de un salario básico unificado del trabajador en general y clausura temporal o definitiva del establecimiento correspondiente, el incumplimiento a lo dispuesto en los artículos 40, 47, 58 inciso segundo, 97, 103 y 124 de esta Ley.

Art. 243.- Será sancionado con multa de cinco salarios básicos unificados del trabajador en general, el incumplimiento a lo dispuesto en los artículos 50, 57, 60, 74 inciso primero, 90, 118, 163, 175, 184

y 202 literal b), de esta Ley.

Art. 244.- Será sancionado con multa de cinco salarios básicos unificados del trabajador en general y clausura temporal o definitiva del establecimiento correspondiente, el incumplimiento a lo dispuesto en los artículos 105, 109, 166, 169, 172, 174, 178, 194, 200 y 212 de esta Ley.

Art. 245.- Será sancionado con multa de cinco salarios básicos unificados del trabajador en general y decomiso, el incumplimiento de lo dispuesto en el artículo 125 y primer inciso del artículo 153 de esta Ley.

Art. 246.- Será sancionado con multa de diez salarios básicos unificados del trabajador en general, el incumplimiento a lo dispuesto en los artículos 12 inciso segundo, 22, 29, 41, 48, 80, 110, 173 y 202 literal c), de esta Ley.

Art. 247.- Será sancionado con multa de diez salarios básicos unificados del trabajador en general y clausura temporal o definitiva del establecimiento correspondiente, el incumplimiento a lo dispuesto en los artículos 104, 116, 121, 143, 159 inciso segundo, 186 y 192 incisos segundo y tercero, de esta Ley.

Art. 248.- Será sancionado con multa de diez salarios básicos unificados del trabajador en general, decomiso y clausura temporal o definitiva del establecimiento correspondiente, el incumplimiento a lo dispuesto en los artículos 42, 49, 137, 140, 141 inciso primero, 146, 164 y 170 de esta Ley.

Art. 249.- Será sancionado con multa de veinte salarios básicos unificados del trabajador en general, el incumplimiento a lo dispuesto en los artículos 27 inciso segundo, 75, 162 y 202 literal d), de esta Ley.

Art. 250.- Será sancionado con multa de veinte salarios básicos unificados del trabajador en general y clausura temporal o definitiva, el incumplimiento a lo dispuesto en los artículos 149 y 183 inciso segundo, de esta Ley.

Art. 251.- Será sancionado con multa de veinte salarios básicos unificados del trabajador en general, decomiso y clausura temporal o definitiva del establecimiento correspondiente, el incumplimiento a lo dispuesto en los artículos 150 y 151, de esta Ley.

Art. 252.-Nota: Artículo derogado por Ley No. 00, publicada en Registro Oficial 398 de 4 de Marzo del 2011 .

Art. 253.- La infracción a lo dispuesto en los artículos 213 y 214, será sancionada con suspensión del ejercicio profesional, multa de cien salarios básicos unificados del trabajador en general y clausura definitiva del establecimiento, sin perjuicio de las sanciones civiles y penales a que hubiere lugar.

Art. 254.- Será sancionado con multa de cinco salarios básicos unificados del trabajador en general, el incumplimiento a lo dispuesto en los artículos 130 y 134 de esta Ley.

Art. 255.- Será sancionado con la suspensión del ejercicio profesional por cinco años y clausura temporal o definitiva del establecimiento correspondiente, el incumplimiento a lo dispuesto en el artículo 210 de esta Ley.

Art. 256.- Será sancionado con clausura temporal o definitiva del establecimiento correspondiente, el incumplimiento a lo dispuesto en el artículo 185 de esta Ley.

Art. 257.- El producto de las multas que se recauden por infracciones a lo dispuesto por esta Ley y sus reglamentos, será utilizado en la respectiva jurisdicción en donde se las impusiere, debiendo destinarlo para la atención y mejoramiento de los servicios de salud de la respectiva dirección

provincial.

Art. 258.- Para el cumplimiento de las disposiciones establecidas en la presente Ley, las autoridades de salud tendrán libre acceso a los lugares en los cuales deban cumplir sus funciones de inspección y control, pudiendo al efecto requerir la intervención de la fuerza pública, en caso de ser necesario.

CAPITULO V
De las definiciones

Art. 259.- Para efectos de esta Ley, se entiende por:

Acreditación de servicios de salud.- Es el proceso voluntario realizado con regularidad y periodicidad, de carácter reservado, a través del cual un servicio de salud, independientemente de su nivel es evaluado por un organismo técnico calificado, de acuerdo a un conjunto de normas que describe las actividades y estructuras que contribuyen en forma directa a los resultados deseados para los pacientes-usuarios, el cumplimiento de estas normas busca alcanzar un óptimo nivel de calidad de atención teniendo en cuenta los recursos disponibles.

Aditivos alimentarios.- Son sustancias o mezclas de sustancias de origen natural o artificial, que por sí solas no se consumen directamente como alimentos, tengan o no valor nutritivo y se adicionan en límites permitidos durante la producción, manipulación, fabricación, elaboración, tratamiento o conservación de alimentos.

Agentes de las medicinas tradicionales.- Son aquellos sanadores que intervienen en diferentes ámbitos de la salud, cuyas denominaciones son particulares a cada una de las nacionalidades y pueblos, y su reconocimiento proviene de las propias comunidades donde prestan sus servicios. Las condiciones y características formales y temporales de su formación son propias de su tradición y cultura ancestral.

Alimento.- Es todo producto natural o artificial que ingerido aporta al organismo de los seres humanos o de los animales, los materiales y la energía necesarios para el desarrollo de los procesos biológicos.

Comprende también las sustancias y mezclas de las mismas que se ingieren por hábito o costumbre, tengan o no valor nutritivo.

Alimentos genéticamente modificados.- Son aquellos que contienen o están compuestos por organismos genéticamente modificados o han sido producidos a partir de ellos.

Alimento natural.- Es aquel que se utiliza como se presenta en la naturaleza sin haber sufrido transformación en sus caracteres o en su composición, pudiendo ser sometido a procesos prescritos por razones de higiene, o las necesarias para la separación de partes no comestibles.

Alimento procesado.- Es toda materia alimenticia natural o artificial que para el consumo humano ha sido sometida a operaciones tecnológicas necesarias para su transformación, modificación y conservación, que se distribuye y comercializa en envases rotulados bajo una marca de fábrica determinada.

El término alimento procesado, se extiende a bebidas alcohólicas y no alcohólicas, aguas de mesa, condimentos, especias y aditivos alimentarios.

Atención farmacéutica.- Es la asistencia al paciente por parte del químico farmacéutico o bioquímico farmacéutico en el seguimiento del tratamiento fármaco terapéutico, dirigido a contribuir con el médico y otros profesionales de la salud, en la consecución de los resultados previstos y el logro del máximo beneficio terapéutico.

Bancos de sangre.- Son servicios de salud, técnicos, especializados y calificados, encargados de realizar la extracción, preparación, conservación, almacenamiento y suministro de la sangre humana, sus componentes y derivados.

Bancos de tejidos.- Son servicios de salud técnicos, especializados y calificados, que tienen por misión garantizar la calidad de los tejidos desde su obtención hasta su utilización clínica.

Botiquines.- Son establecimientos farmacéuticos autorizados para expender al público, únicamente la lista de medicamentos y otros productos que determine la autoridad sanitaria nacional; funcionarán en zonas rurales en las que no existan farmacias y deben cumplir en todo tiempo con prácticas adecuadas de almacenamiento.

Casas de representación.- Son los establecimientos farmacéuticos autorizados para realizar promoción médica, importación y venta al por mayor a terceros de los productos elaborados por sus representados. Deben cumplir con buenas prácticas de almacenamiento y distribución determinadas por la autoridad sanitaria nacional. Requieren para su funcionamiento de la dirección técnica responsable de un profesional químico farmacéutico o bioquímico farmacéutico.

Ciclo producción - consumo.- Son las etapas o fases involucradas en la producción, manipulación, almacenamiento, transporte, distribución, importación, exportación, comercialización, expendio y consumo de productos.

Componentes anatómicos.- Son los órganos, tejidos, células, sus derivados y en general todas las partes del organismo humano.

Desechos.- Son los residuos o desperdicios en cualquier estado de la materia, producto de actividades industriales, comerciales y de la comunidad; se clasifican en comunes, infecciosos y especiales o peligrosos.

Desechos comunes.- Son aquellos que no representan riesgo para la salud humana, animal o el ambiente.

Desechos peligrosos.- Son aquellos resultantes de un proceso de producción, transformación, reciclaje, utilización o consumo y que tengan algún compuesto con características reactivas, inflamables, corrosivas, infecciosas o tóxicas, que presenten un riesgo para la salud humana, los recursos naturales y el ambiente.

Desechos infecciosos.- Son aquellos que contienen gérmenes patógenos y representan riesgo para la salud; se generan en los establecimientos de salud humana, veterinarios, morgues y otros.

Dispositivos médicos.- Son los artículos, instrumentos, aparatos, artefactos o invenciones mecánicas, incluyendo sus componentes, partes o accesorios, fabricado, vendido o recomendado para uso en diagnóstico, tratamiento curativo o paliativo, prevención de una enfermedad, trastorno o estado físico anormal o sus síntomas, para reemplazar o modificar la anatomía o un proceso fisiológico o controlarla. Incluyen las amalgamas, barnices, sellantes y más productos dentales similares.

Distribuidoras farmacéuticas.- Son establecimientos farmacéuticos autorizados para realizar importación, exportación y venta al por mayor de medicamentos en general de uso humano, especialidades farmacéuticas, productos para la industria farmacéutica, auxiliares médico-quirúrgico, dispositivos médicos, insumos médicos, cosméticos y productos higiénicos. Deben cumplir con las buenas prácticas de almacenamiento y distribución determinadas por la autoridad sanitaria nacional. Funcionarán bajo la representación y responsabilidad técnica de un químico farmacéutico o bioquímico farmacéutico.

Donante.- Es la persona de la cual, durante su vida o después de su muerte, se extraen

componentes anatómicos en buen estado funcional, para trasplantarlos en otra persona o utilizarlos con fines terapéuticos o de investigación.

Enfermedad Catastrófica.- Es aquella que cumple con las siguientes características:

a) Que implique un alto riesgo para la vida de la persona;
b) Que sea una enfermedad crónica y por lo tanto que su atención no sea emergente; y,
c) Que su tratamiento pueda ser programado o que el valor promedio de su tratamiento mensual sea mayor al determinado en el Acuerdo Ministerial de la Autoridad Sanitaria.

Enfermedades Raras y Huérfanas: Las enfermedades raras o huérfanas, incluidas las de origen genético, son aquellas enfermedades potencialmente mortales, o debilitantes a largo plazo, de baja prevalencia y de alta complejidad.

Nota: Definiciones de Enfermedades Catastrófica, Raras y Huérfanas agregadas por Ley No. 0, publicada en Registro Oficial 625 de 24 de Enero del 2012 .

Emergencia sanitaria.- Es toda situación de riesgo de afección de la salud originada por desastres naturales o por acción de las personas, fenómenos climáticos, ausencia o precariedad de condiciones de saneamiento básico que favorecen el incremento de enfermedades transmisibles. Requiere la intervención especial del Estado con movilización de recursos humanos, financieros u otros, destinados a reducir el riesgo o mitigar el impacto en la salud de las poblaciones más vulnerables.

La emergencia sanitaria deberá ser declarada por el Presidente de la República conforme lo manda la Constitución Política.

Farmacias.- Son establecimientos farmacéuticos autorizados para la dispensación y expendio de medicamentos de uso y consumo humano, especialidades farmacéuticas, productos naturales procesados de uso medicinal, productos biológicos, insumos y dispositivos médicos, cosméticos, productos dentales, así como para la preparación y venta de fórmulas oficinales y magistrales. Deben cumplir con buenas prácticas de farmacia. Requieren para su funcionamiento la dirección técnica y responsabilidad de un profesional químico farmacéutico o bioquímico farmacéutico.

Genética.- Es la ciencia que trata de la reproducción, herencia, variación tanto en estado normal como anormal o de enfermedad, y del conjunto de fenómenos y problemas relativos a la descendencia.

Laboratorios farmacéuticos.- Son establecimientos farmacéuticos autorizados para producir o elaborar medicamentos en general, especialidades farmacéuticas, biológicos de uso humano o veterinario; deben cumplir las normas de buenas prácticas de manufactura determinadas por la autoridad sanitaria nacional; y, estarán bajo la dirección técnica de químicos farmacéuticos o bioquímicos farmacéuticos.

Licenciamiento de servicios de salud.- Es el procedimiento de carácter obligatorio por medio del cual la autoridad sanitaria nacional otorga el permiso de funcionamiento a las instituciones prestadoras de servicios de salud, públicas o privadas, según su capacidad resolutiva, niveles de atención y complejidad, previa verificación del cumplimiento de los requisitos o estándares mínimos indispensables.

Materia prima alimentaria.- Es la sustancia o mezcla de sustancias, natural o artificial permitida por la autoridad sanitaria nacional, que se utiliza para la elaboración de alimentos y bebidas.

Medicamento.- Es toda preparación o forma farmacéutica, cuya fórmula de composición expresada en unidades del sistema internacional, está constituida por una sustancia o mezcla de sustancias, con peso, volumen y porcentajes constantes, elaborada en laboratorios farmacéuticos legalmente

establecidos, envasada o etiquetada para ser distribuida y comercializada como eficaz para diagnóstico, tratamiento, mitigación y profilaxis de una enfermedad, anomalía física o síntoma, o el restablecimiento, corrección o modificación del equilibrio de las funciones orgánicas de los seres humanos y de los animales.

Por extensión esta definición se aplica a la asociación de sustancias de valor dietético, con indicaciones terapéuticas o alimentos especialmente preparados, que reemplacen regímenes alimenticios especiales.

Medicamento de venta libre.- Es el medicamento oral o tópico que por su composición y por la acción farmacológica de sus principios activos, es autorizado para ser expendido o dispensado sin prescripción facultativa.

Medicamento genérico.- Es aquel que se registra y comercializa con la Denominación Común Internacional (DCI) del principio activo, propuesta por la Organización Mundial de la Salud; o en su ausencia, con una denominación genérica convencional reconocida internacionalmente. Estos medicamentos deben mantener los niveles de calidad, seguridad y eficacia requeridos para los de marca.

Medicamento homeopático.- Es el preparado farmacéutico obtenido por técnicas homeopáticas, conforme a las reglas descritas en las farmacopeas oficiales aceptadas en el país, con el objeto de prevenir la enfermedad, aliviar, curar, tratar y rehabilitar a un paciente. Los envases, rótulos, etiquetas y empaques hacen parte integral del medicamento, por cuanto éstos garantizan su calidad, estabilidad y uso adecuado. Deben ser prescritos por profesionales autorizados para el efecto y dispensados o expendidos en lugares autorizados para el efecto.

Medicinas alternativas.- Son el conjunto de medicinas científicamente comprobadas, ejercidas por profesionales médicos, con título de cuarto nivel en la materia y reconocidas por la autoridad sanitaria nacional.

Medicinas tradicionales.- Son el conjunto de conocimientos y prácticas ancestrales de las nacionalidades, pueblos, comunidades indígenas, mestizas y afro descendientes que a lo largo del tiempo han constituido un saber específico, mantenido y difundido en un contexto cultural, de interrelación de elementos naturales, éticos, espirituales, mentales, psicológicos y afectivos y que se explica y funciona en ese mismo universo cultural. Sus prácticas se corresponden con saberes, técnicas y procedimientos propios de su cosmovisión y son ejercidas por sanadores de las medicinas tradicionales, reconocidos por sus comunidades y registrados por la autoridad sanitaria nacional.

Necropsia o autopsia.- Es el procedimiento técnico mediante el cual se observa y analiza un cadáver, externa e internamente para establecer las causas del fallecimiento de la persona.

Notificación sanitaria.- Es la comunicación mediante la cual el interesado informa a entidad competente de la Autoridad Sanitaria Nacional, bajo declaración jurada, que comercializará en el país un producto de uso o consumo humano, fabricado en el territorio nacional o en el exterior cumpliendo con condiciones de calidad, seguridad e inocuidad.

Nueva entidad química.- Es el medicamento, ingrediente o principio activo de uso o consumo humano que nunca ha sido empleado para ninguna indicación terapéutica en el ámbito mundial. No se considerará nueva entidad química entre otros, los nuevos usos o segundos usos, ni las novedades o cambio de los siguientes aspectos: formas farmacéuticas, indicaciones o segundas indicaciones, nuevas combinaciones de entidades químicas conocidas, formulaciones, formas de dosificación, vías de administración, modificaciones de cualquier índole que no afecten el mecanismo de acción, condiciones de comercialización y empaque y en general, aquellas que impliquen nuevas presentaciones.

Organismos genéticamente modificados, OGM u organismo vivo modificado OVM.- Cualquier

organismo vivo, con excepción de los seres humanos, que ha adquirido una combinación genética novedosa, generada a través del uso específico de técnicas de la biotecnología moderna.

Permiso de funcionamiento.- Es el documento otorgado por la autoridad sanitaria nacional a los establecimientos sujetos a control y vigilancia sanitaria que cumplen con todos los requisitos para su funcionamiento, establecidos en los reglamentos correspondientes.

Plantas procesadoras de alimentos.- Son establecimientos en los que se realizan operaciones de selección, purificación y transformación de materias primas para la producción, envasado y etiquetado de alimentos.

Producto natural procesado de uso medicinal.- Es el producto medicinal terminado y etiquetado, cuyos ingredientes activos están formados por cualquier parte de los recursos naturales de uso medicinal o sus combinaciones, como droga cruda, extracto o en una forma farmacéutica reconocida, que se utiliza con fines terapéuticos

No se considera un producto natural procesado de uso medicinal, si el recurso natural de uso medicinal se combina con sustancias activas definidas desde el punto de vista químico, inclusive constituyentes de recursos naturales, aislados y químicamente definidos.

Producto nutracéutico.- También llamado funcional o compuesto bioactivo, es cualquier producto semejante en apariencia a un alimento convencional que tiene uno o más beneficios intencionales, más allá de proporcionar una nutrición adecuada, como un mejor estado de salud o una reducción del riesgo de una enfermedad determinada.

Productos del tabaco.- Abarca los productos preparados totalmente o en parte utilizando como materia prima hojas de tabaco y destinados a ser fumados, chupados, inhalados, mascados o utilizados como rapé.

Reactivos bioquímicos.- Son todas las sustancias o productos que se utilizan con máquinas especiales o no, para reaccionar con líquidos o materias orgánicas y ayudar en el diagnóstico, monitoreo, control y tratamiento de las enfermedades de los seres humanos.

Receptor.- Es la persona en cuyo cuerpo se implantan componentes anatómicos provenientes de sí mismo, de otra persona o de otra especie.

Registro sanitario.- Es la certificación otorgada por la autoridad sanitaria nacional, para la importación, exportación y comercialización de los productos de uso y consumo humano señalados en el artículo 137 de esta Ley. Dicha certificación es otorgada cuando se cumpla con los requisitos de calidad, seguridad, eficacia y aptitud para consumir y usar dichos productos cumpliendo los trámites establecidos en la presente Ley y sus reglamentos.

Salud ambiental.- Son los conocimientos que se ocupan de las formas de vida, sustancias, fuerzas y condiciones del entorno del ser humano que pueden ejercer efectos nocivos sobre su salud y bienestar, así como las acciones para impedirlos o reducirlos, en el marco de la promoción y desarrollo de ambientes saludables.

Salud reproductiva.- Es el estado general de bienestar físico, mental y social y no de mera ausencia de enfermedades o dolencias, en todos los aspectos relacionados con el sistema reproductivo, sus funciones y procesos e implica el derecho de las personas a tomar decisiones respecto a ella.

Salud sexual.- Es el estado general de bienestar físico, mental y social y no de mera ausencia de enfermedades o dolencias, que permita a la persona en forma libre y responsable disfrutar de una vida sexual plena, placentera, libre de abuso sexual, coerción o acoso y de enfermedades sexualmente transmisibles.

Saneamiento ambiental.- Es el conjunto de actividades dedicadas a acondicionar, controlar y proteger el ambiente en que vive el ser humano, a fin de proteger su salud.

Servicios de salud.- Son aquellos que están destinados a brindar prestaciones de salud, de promoción, de prevención, de recuperación y rehabilitación en forma ambulatoria, domiciliaria o internamiento, son clasificados de acuerdo a la capacidad resolutiva, niveles de atención y complejidad.

Terapias alternativas.- Conjunto de métodos, técnicas y sistemas utilizados para prevención o tratamiento de enfermedades y se orientan a equilibrar el organismo en sus aspectos físico, mental o espiritual, y a establecer un balance entre el individuo y el entorno.

Trasplante de órganos.- Es la sustitución con fines terapéuticos de componentes anatómicos en una persona, por otros iguales y funcionales provenientes del mismo receptor o de un donante vivo o muerto.

Violencia.- Es toda acción, omisión o uso intencional de la fuerza física o el poder, real o por amenaza, de una persona, grupo o institución con el fin de dañar a otra en contra de su voluntad, caracterizada por la agresión contra la integridad física, sexual, psicológica, simbólica o cultural.

Nota: Definición Notificación Sanitaria agregada por Disposición reformatoria Séptima de Ley No. 0, publicada en Registro Oficial Suplemento 652 de 18 de diciembre del 2015 .

DISPOSICIONES GENERALES

PRIMERA.- Los servicios de control, inspecciones, autorizaciones, permisos, licencias, registros y otros de similar naturaleza que preste la autoridad sanitaria nacional, satisfarán el pago de derechos de conformidad con los reglamentos respectivos.

PRIMERA-A.- El ministerio encargado del ramo de la inclusión económica y social ejecutará los programas de atención y protección social a las familias que tengan entre sus miembros a pacientes que sufran enfermedades consideradas raras o huérfanas y catastróficas mediante la aplicación de políticas de inclusión y cohesión social, igualdad y protección integral en coordinación con la Autoridad Sanitaria Nacional.

Nota: Disposición agregada por Ley No. 0, publicada en Registro Oficial 625 de 24 de Enero del 2012 .

SEGUNDA.- El Presidente de la República dictará el reglamento general para la aplicación de la presente Ley, en un plazo máximo de noventa días.

TERCERA.- Derogatorias.- Deróganse todas las normas, disposiciones generales o especiales que se opongan a la presente Ley, en materia de salud.

Deróganse expresamente:

El Código de la Salud, expedido mediante Decreto Ejecutivo No. 188, publicado en el Registro Oficial No. 158 del 8 de febrero de 1971 y todas sus reformas.

Los artículos 8, 9, 10, 11 y el Capítulo VIII de la Ley de Producción, Importación, Comercialización y Expendio de Medicamentos Genéricos de Uso Humano, publicada en el Registro Oficial No. 59 de 17 de abril del 2000 .

El artículo 99 de la Ley de Promoción de la Inversión y Participación Ciudadana, publicada en el Registro Oficial Suplemento No. 144, de 18 de agosto del 2000 .

El artículo 11 de la Ley de Trasplantes de Organos y Tejidos, publicada en Registro Oficial 492 de 27 de Julio de 1994 .

CUARTA.- Efectúense las siguientes reformas:

a) En el artículo 2 de la Ley de Producción, Importación, Comercialización y Expendio de Medicamentos Genéricos de Uso Humano, publicada en el Registro Oficial No. 59 de 17 de abril del 2000 , sustitúyase la palabra: "...emplean", por: "comercializan...";
b) En toda la Ley de Derechos y Amparo al Paciente, sustitúyase: "centros de salud", por: "servicios de salud";
c) En el artículo 98 de la Ley de Registro Civil, Identificación y Cedulación, a continuación del numeral 13, agréguese el siguiente:

"14. Autorización expresa del ciudadano para ser donante de órganos u otros componentes anatómicos, de conformidad con lo establecido en el artículo 83 de la Ley Orgánica de Salud.";

d) Sustitúyanse los artículos 1 y 2 de la Ley de Aprovisionamiento y Utilización de Sangre y sus Derivados, publicada en Registro Oficial 559 de 7 de Noviembre de 1986 , por los siguientes.

"**Art. 1**.- La vigilancia y control del aprovisionamiento y utilización de sangre y sus derivados en el Ecuador, será responsabilidad de la autoridad sanitaria nacional.

Organizará en coordinación con la Cruz Roja Ecuatoriana, el sistema nacional de bancos y depósitos de sangre, en las ciudades y servicios de salud que los requieran, siempre que cuenten con las condiciones técnicas para ello.

La Cruz Roja Ecuatoriana, el Ministerio de Salud Pública, el Instituto Ecuatoriano de Seguridad Social, las Fuerzas Armadas y la Junta de Beneficencia de Guayaquil continuarán administrando los bancos y depósitos de sangre adscritos a sus servicios de salud.

Art. 2.- Prohíbese la exportación de sangre y sus derivados, salvo lo señalado en el artículo 79 de la Ley Orgánica de Salud."; y,

e) A continuación del inciso quinto del artículo 46 de la Ley Orgánica de Aduanas, inclúyase otro inciso con el siguiente texto:

"Se exceptúan también de la verificación en origen: las vacunas, biológicos, medicamentos e insumos importados por el Ministerio de Salud Pública.

QUINTA.- Todas las capitales de provincia, sin excepción, contarán al menos con un hospital público de tercer nivel de atención, con la infraestructura, equipamiento, bienes, insumos, presupuestos y recursos humanos idóneos, suficientes y permanentes, facultados para dar solución a las necesidades de la población, de conformidad con la realidad epidemiológica local.

SEXTA.- Conforme lo previsto en el numeral 10 del artículo 35 de la Constitución Política de la República, se prohíbe la paralización, a cualquier título o por algún motivo, del servicio público de salud, bajo prevención de aplicar a los responsables las sanciones previstas en las leyes que regulan la relación laboral y el Código Penal.

DISPOSICIONES TRANSITORIAS

Nota: Denominación de título reformado por Ley No. 0, publicada en Registro Oficial 625 de 24 de Enero del 2012 .

Los reglamentos que sobre asuntos de salud están vigentes, seguirán aplicándose en todo lo que no se oponga a la presente Ley, hasta cuando se dicten otros.

Dada, en la ciudad de San Francisco de Quito, Distrito Metropolitano, en la sala de sesiones del Congreso Nacional del Ecuador, a los catorce días del mes de diciembre del año dos mil seis.

PRIMERA.- Una vez publicada la Ley Orgánica Reformatoria a la Ley Orgánica de Salud para incluir el Tratamiento de las Enfermedades Raras o Huérfanas y Catastróficas, el Ministerio de Salud Pública emitirá y actualizará la lista de enfermedades consideradas raras o huérfanas, al menos cada dos años tomando en cuenta las enfermedades consideradas raras o ultra raras por la Organización Mundial de la Salud/Organización Panamericana de la Salud.

En el plazo de ciento ochenta días, el Ministerio de Salud Pública, dictará los acuerdos, resoluciones y demás normas técnicas para la efectiva aplicación de la Ley Orgánica Reformatoria a la Ley Orgánica de Salud para Incluir el Tratamiento de las Enfermedades Raras o Huérfanas y Catastróficas.

Nota: Disposición Primera agregada por Ley No. 0, publicada en Registro Oficial 625 de 24 de Enero del 2012 .

SEGUNDA.- Una vez publicada la Ley Orgánica Reformatoria a la Ley Orgánica de Salud para incluir el Tratamiento de las Enfermedades Raras o Huérfanas y Catastróficas, todos los programas de atención para enfermedades catastróficas que se estén ejecutando en cualquier dependencia pública, pasarán a depender del Ministerio de Salud Pública, quien se encargará de continuar con su ejecución.

Nota: Disposición agregada por Ley No. 0, publicada en Registro Oficial 625 de 24 de Enero del 2012 .

TERCERA.- Una vez publicada la Ley Orgánica Reformatoria a la Ley Orgánica de Salud para incluir el Tratamiento de las Enfermedades Raras o Huérfanas y Catastróficas, el Ministerio de Finanzas procederá a realizar la correspondiente reclasificación presupuestaria, dentro del Presupuesto General del Estado, para que el Ministerio de Salud Pública cuente con los fondos necesarios y pueda cumplir las obligaciones determinadas en esta Ley.

Nota: Disposición agregada por Ley No. 0, publicada en Registro Oficial 625 de 24 de Enero del 2012 .

LEY ORGANICA DE DEFENSA DEL CONSUMIDOR

Ley 21
Registro Oficial Suplemento 116 de 10-jul-2000
Ultima modificación: 13-oct-2011
Estado: Vigente

EL CONGRESO NACIONAL

Considerando:

Que, la generalidad de ciudadanos ecuatorianos son víctimas permanentes de todo tipo de abusos por parte de empresas públicas y privadas de las que son usuarios y consumidores;

Que, de conformidad con lo dispuesto por el numeral 7 del artículo 23 de la Constitución Política de la República, es deber del Estado garantizar el derecho a disponer de bienes y servicios públicos y privados, de óptima calidad; a elegirlos con libertad, así como a recibir información adecuada y veraz sobre su contenido y características;

Que, el Art. 92 de la Constitución Política de la República dispone que la ley establecerá los mecanismos de control de calidad, los procedimientos de defensa del consumidor, la reparación e indemnización por deficiencias, daños y mala calidad de bienes y servicios, y por la interrupción de los servicios públicos no ocasionados por catástrofes, caso fortuito o fuerza mayor, y las sanciones por la violación de estos derechos;

Que, el artículo 244, numeral 8 de la Carta Fundamental señala que al Estado le corresponderá proteger los derechos de los consumidores, sancionar la información fraudulenta, la publicidad engañosa, la adulteración de los productos, la alteración de pesos y medidas, y el incumplimiento de las normas de calidad;

Que, la Ley de Defensa del Consumidor publicada en el Registro Oficial No. 520 de septiembre 12 de 1990 , a consecuencia de todas sus reformas se ha tornado inoperante e impracticable; más aún si se considera que dicha ley atribuía competencia para su ejecución a diversos organismos; sin que ninguno de ellos haya asumido en la práctica tales funciones;

Que, la Constitución Política de la República en su artículo 96 faculta al Defensor del Pueblo para defender y excitar la observancia de los derechos fundamentales consagrados en ella, así como para observar la calidad de los servicios públicos;

Que, en la actualidad la Defensoría del Pueblo, pese a sus limitaciones, ha asumido de manera eficiente la defensa de los intereses del consumidor y el usuario, a través de la Defensoría Adjunta del Consumidor y Usuario; y,

En ejercicio de sus facultades constitucionales y legales expide la siguiente.

LEY ORGANICA DE DEFENSA DEL CONSUMIDOR

CAPITULO I
PRINCIPIOS GENERALES

Art. 1.- Ambito y Objeto.- Las disposiciones de la presente Ley son de orden público de interés social, sus normas por tratarse de una Ley de carácter orgánico, prevalecerán sobre las disposiciones contenidas en leyes ordinarias. En caso de duda en la interpretación de esta Ley, se la aplicará en el sentido más favorable al consumidor.

El objeto de esta Ley es normar las relaciones entre proveedores y consumidores, promoviendo el conocimiento y protegiendo los derechos de los consumidores y procurando la equidad y la seguridad jurídica en dichas relaciones entre las partes.

Art. 2.- Definiciones.- Para efectos de la presente ley, se entenderá por:

Anunciante.- Aquel proveedor de bienes o de servicios que ha encargado la difusión pública de un mensaje publicitario o de cualquier tipo de información referida a sus productos o servicios.

Consumidor.- Toda persona natural o jurídica que como destinatario final adquiera utilice o disfrute bienes o servicios, o bien reciba oferta para ello. Cuando la presente ley mencione al Consumidor, dicha denominación incluirá al Usuario.

Contrato de Adhesión.- Es aquel cuyas cláusulas han sido establecidas unilateralmente por el proveedor a través de contratos impresos o en formularios sin que el consumidor, para celebrarlo, haya discutido su contenido.

Derecho de Devolución.- Facultad del consumidor para devolver o cambiar un bien o servicio, en los plazos previstos en esta Ley, cuando no se encuentra satisfecho o no cumple sus expectativas, siempre que la venta del bien o servicio no haya sido hecha directamente, sino por correo, catálogo, teléfono, internet, u otros medios similares.

Especulación.- Práctica comercial ilícita que consiste en el aprovechamiento de una necesidad del mercado para elevar artificiosamente los precios, sea mediante el ocultamiento de bienes o servicios, o acuerdos de restricción de ventas entre proveedores, o la renuencia de los proveedores a atender los pedidos de los consumidores pese a haber existencias que permitan hacerlo, o la elevación de los precios de los productos por sobre los índices oficiales de inflación, de precios al productor o de precios al consumidor.

Información Básica Comercial.- Consiste en los datos, instructivos, antecedentes, indicaciones o contraindicaciones que el proveedor debe suministrar obligatoriamente al consumidor, al momento de efectuar la oferta del bien o prestación del servicio.

Oferta.- Práctica comercial consistente en el ofrecimiento de bienes o servicios que efectúa el proveedor al consumidor.

Proveedor.- Toda persona natural o jurídica de carácter público o privado que desarrolle actividades de producción, fabricación, importación, construcción, distribución, alquiler o comercialización de bienes, así como prestación de servicios a consumidores, por lo que se cobre precio o tarifa. Esta definición incluye a quienes adquieran bienes o servicios para integrarlos a procesos de producción o transformación, así como a quienes presten servicios públicos por delegación o concesión.

Publicidad.- La comunicación comercial o propaganda que el proveedor dirige al consumidor por cualquier medio idóneo, para informarlo y motivarlo a adquirir o contratar un bien o servicio. Para el efecto la información deberá respetar los valores de identidad nacional y los principios fundamentales sobre seguridad personal y colectiva.

Publicidad Abusiva.- Toda modalidad de información o comunicación comercial, capaz de incitar a la violencia, explotar el miedo, aprovechar la falta de madurez de los niños y adolescentes, alterar la paz y el orden público o inducir al consumidor a comportarse en forma perjudicial o peligrosa para la salud y seguridad personal y colectiva.

Se considerará también publicidad abusiva toda modalidad de información o comunicación comercial que incluya mensajes subliminales.

Publicidad Engañosa.- Toda modalidad de información o comunicación de carácter comercial, cuyo contenido sea total o parcialmente contrario a las condiciones reales o de adquisición de los bienes y servicios ofrecidos o que utilice textos, diálogos, sonidos, imágenes o descripciones que directa o indirectamente, e incluso por omisión de datos esenciales del producto, induzca a engaño, error o confusión al consumidor.

Servicios Públicos Domiciliarios.- Se entienden por servicios públicos domiciliarios los prestados directamente en los domicilios de los consumidores, ya sea por proveedores públicos o privados tales como servicio de energía eléctrica, telefonía convencional, agua potable u otros similares.

Distribuidores o Comerciantes.- Las personas naturales o jurídicas que de manera habitual venden o proveen al por mayor o al detal, bienes destinados finalmente a los consumidores, aún cuando ello no se desarrolle en establecimientos abiertos al público.

Productores o Fabricantes.- Las personas naturales o jurídicas que extraen, industrializan o transforman bienes intermedios o finales para su provisión a los consumidores.

Importadores.- Las personas naturales o jurídicas que de manera habitual importan bienes para su venta o provisión en otra forma al interior del territorio nacional.

Prestadores.- Las personas naturales o jurídicas que en forma habitual prestan servicios a los consumidores.

Art. 3.- Derechos y Obligaciones Complementarias.- Los derechos y obligaciones establecidas en la presente ley no excluyen ni se oponen a aquellos contenidos en la legislación destinada a regular la protección del medio ambiente y el desarrollo sustentable, u otras leyes relacionadas.

Concordancias:

CONSTITUCION DE LA REPUBLICA DEL ECUADOR, Arts. 14, 275, 395

CAPITULO II
DERECHOS Y OBLIGACIONES
DE LOS CONSUMIDORES

Art. 4.- Derechos del Consumidor.- Son derechos fundamentales del consumidor, a más de los establecidos en la Constitución Política de la República, tratados o convenios internacionales, legislación interna, principios generales del derecho y costumbre mercantil, los siguientes:

1. Derecho a la protección de la vida, salud y seguridad en el consumo de bienes y servicios, así como a la satisfacción de las necesidades fundamentales y el acceso a los servicios básicos;
2. Derecho a que proveedores públicos y privados oferten bienes y servicios competitivos, de óptima calidad, y a elegirlos con libertad; 3. Derecho a recibir servicios básicos de óptima calidad;
4. Derecho a la información adecuada, veraz, clara, oportuna y completa sobre los bienes y servicios ofrecidos en el mercado, así como sus precios, características, calidad, condiciones de contratación y demás aspectos relevantes de los mismos, incluyendo los riesgos que pudieren presentar;
5. Derecho a un trato transparente, equitativo y no discriminatorio o abusivo por parte de los proveedores de bienes o servicios, especialmente en lo referido a las condiciones óptimas de calidad, cantidad, precio, peso y medida;
6. Derecho a la protección contra la publicidad engañosa o abusiva, los métodos comerciales coercitivos o desleales;
7. Derecho a la educación del consumidor, orientada al fomento del consumo responsable y a la difusión adecuada de sus derechos;
8. Derecho a la reparación e indemnización por daños y perjuicios, por deficiencias y mala calidad de bienes y servicios;
9. Derecho a recibir el auspicio del Estado para la constitución de asociaciones de consumidores y

usuarios, cuyo criterio será consultado al momento de elaborar o reformar una norma jurídica o disposición que afecte al consumidor; y,
10. Derecho a acceder a mecanismos efectivos para la tutela administrativa y judicial de sus derechos e intereses legítimos, que conduzcan a la adecuada prevención sanción y oportuna reparación de su lesión;
11. Derecho a seguir las acciones administrativas y/o judiciales que correspondan; y,
12. Derecho a que en las empresas o establecimientos se mantenga un libro de reclamos que estará a disposición del consumidor, en el que se podrá notar el reclamo correspondiente, lo cual será debidamente reglamentado.

Concordancias:

CONSTITUCION DE LA REPUBLICA DEL ECUADOR, Arts. 11, 52, 54, 55, 66

LEY ORGANICA DE EMPRESAS PUBLICAS, LOEP, Arts. 18

Art. 5.- Obligaciones del Consumidor.- Son obligaciones de los consumidores:

1. Propiciar y ejercer el consumo racional y responsable de bienes y servicios;
2. Preocuparse de no afectar el ambiente mediante el consumo de bienes o servicios que puedan resultar peligrosos en ese sentido;
3. Evitar cualquier riesgo que pueda afectar su salud y vida, así como la de los demás, por el consumo de bienes o servicios lícitos; y,
4. Informarse responsablemente de las condiciones de uso de los bienes y servicios a consumirse.

Concordancias:

CONSTITUCION DE LA REPUBLICA DEL ECUADOR, Arts. 15

CAPITULO III
REGULACION DE LA PUBLICIDAD
Y SU CONTENIDO

Art. 6.- Publicidad Prohibida.- Quedan prohibidas todas las formas de publicidad engañosa o abusiva, o que induzcan a error en la elección del bien o servicio que puedan afectar los intereses y derechos del consumidor.

Concordancias:

CONSTITUCION DE LA REPUBLICA DEL ECUADOR, Arts. 52

Art. 7.- Infracciones Publicitarias.- Comete infracción a esta Ley el proveedor que a través de cualquier tipo de mensaje induce al error o engaño en especial cuando se refiere a:

1. País de origen, comercial o de otra índole del bien ofrecido o sobre el lugar de prestación del servicio pactado o la tecnología empleada;
2. Los beneficios y consecuencias del uso del bien o de la contratación del servicio, así como el precio, tarifa, forma de pago, financiamiento y costos del crédito;
3. Las características básicas del bien o servicio ofrecidos, tales como componentes, ingredientes, dimensión, cantidad, calidad, utilidad, durabilidad, garantías, contraindicaciones, eficiencia, idoneidad del bien o servicio para los fines que se pretende satisfacer y otras;
4. Los reconocimientos, aprobaciones o distinciones oficiales o privadas, nacionales o extranjeras tales como medallas, premios, trofeos o diplomas.

Nota: El artículo 17 del Código Orgánico Integral Penal dispone: "Se considerarán exclusivamente como infracciones penales las tipificadas en este Código. Las acciones u omisiones punibles, las penas o procedimientos penales previstos en otras normas jurídicas no tendrán validez jurídica

alguna, salvo en materia de niñez y adolescencia.

Art. 8.- Controversias Derivadas de la Publicidad.- En las controversias que pudieren surgir como consecuencia del incumplimiento de lo dispuesto en los artículos precedentes, el anunciante deberá justificar adecuadamente la causa de dicho incumplimiento.

El proveedor, en la publicidad de sus productos o servicios, mantendrá en su poder, para información de los legítimos interesados, los datos técnicos, fácticos y científicos que dieron sustento al mensaje.

CAPITULO IV
INFORMACION BASICA COMERCIAL

Art. 9.- Información Pública.- Todos los bienes a ser comercializados deberán exhibir sus respectivos precios, peso y medidas, de acuerdo a la naturaleza del producto.

Toda información relacionada al valor de los bienes y servicios deberá incluir, además del precio total, los montos adicionales correspondientes a impuestos y otros recargos, de tal manera que el consumidor pueda conocer el valor final.

Además del precio total del bien, deberá incluirse en los casos en que la naturaleza del producto lo permita, el precio unitario expresado en medidas de peso y/o volumen.

Art. 10.- Idioma y Moneda.- Los datos y la información general expuesta en etiquetas, envases, empaques u otros recipientes de los bienes ofrecidos, así como la publicidad, información o anuncios relativos a la prestación de servicios, se expresarán en idioma castellano, en moneda de curso legal y en las unidades de medida de aplicación general en el país; sin perjuicio de que el proveedor pueda incluir, adicionalmente, esos mismos datos en otro idioma, unidad monetaria o de medida.

La información expuesta será susceptible de comprobación.

Art. 11.- Garantía.- Los productos de naturaleza durable tales como vehículos, artefactos eléctricos, mecánicos, electrodomésticos, y electrónicos, deberán ser obligatoriamente garantizados por el proveedor para cubrir deficiencias de la fabricación y de funcionamiento. Las leyendas "garantizado", "garantía" o cualquier otra equivalente sólo podrán emplearse cuando indiquen claramente en que consiste tal garantía; así como las condiciones, forma plazo y lugar en que el consumidor pueda hacerla efectiva.

Toda garantía deberá individualizar a la persona natural o jurídica que la otorga, así como los establecimientos y condiciones en que operará.

Art. 12.- Productos Deficientes o Usados.- Cuando se oferten o expendan al consumidor productos con alguna deficiencia, usados o reconstruidos, tales circunstancias deberán indicarse de manera visible, clara y precisa, en los anuncios, facturas o comprobantes.

Art. 13.- Producción y Transgénica.- Si los productos de consumo humano o pecuario a comercializarse han sido obtenidos o mejorados mediante transplante de genes o, en general, manipulación genética, se advertirá de tal hecho en la etiqueta del producto, en letras debidamente resaltadas.

Concordancias:

CONSTITUCION DE LA REPUBLICA DEL ECUADOR, Arts. 401

Art. 14.- Rotulado Mínimo de Alimentos.- Sin perjuicio de lo que dispongan las normas técnicas al respecto, los proveedores de productos alimenticios de consumo humano deberán exhibir en el rotulado de los productos, obligatoriamente, la siguiente información:

a) Nombre del producto;
b) Marca comercial;
c) Identificación del lote;
d) Razón social de la empresa;
e) Contenido neto;
f) Número de registro sanitario;
g) Valor nutricional;
h) Fecha de expiración o tiempo máximo de consumo;
i) Lista de ingredientes, con sus respectivas especificaciones;
j) Precio de venta al público;
k) País de origen; y,
l) Indicación si se trata de alimento artificial, irradiado o genéticamente modificado.

Concordancias:

LEY ORGANICA DE SALUD, Arts. 137

Art. 15.- Rotulado Mínimo de Medicamentos.- Sin perjuicio de lo establecido en las normas especiales, los medicamentos en general y los productos naturales procesados, deberán contener información sobre:

a) Nombre del producto, genérico o de marca;
b) Marca comercial;
c) Identificación del lote;
d) Razón social de la empresa;
e) Contenido neto;
f) Número de registro sanitario;
g) Fecha de expiración o tiempo máximo de consumo;
h) Lista de componentes, con sus respectivas especificaciones;
i) Precio de venta al público;
j) País de origen;
k) Contraindicaciones;
l) En cuanto a productos naturales, debe identificarse la procedencia, y si hay elementos culturales o étnicos en el origen.

Art. 16.- Información de Bienes de Naturaleza Durable.- A más de la información que el proveedor debe hacer constar para dar cumplimiento a lo dispuesto en los artículos anteriores, cuando se trate de bienes de naturaleza durable, se deberá informar sobre la seguridad de uso, instrucciones sobre un adecuado manejo y advertencias.

CAPITULO V
RESPONSABILIDADES Y OBLIGACIONES
DEL PROVEEDOR

Art. 17.- Obligaciones del Proveedor.- Es obligación de todo proveedor, entregar al consumidor información veraz, suficiente, clara, completa y oportuna de los bienes o servicios ofrecidos, de tal modo que éste pueda realizar una elección adecuada y razonable.

Art. 18.- Entrega del Bien o Prestación del Servicio.- Todo proveedor está en la obligación de entregar o prestar, oportuna y eficientemente el bien o servicio, de conformidad a las condiciones establecidas de mutuo acuerdo con el consumidor. Ninguna variación en cuanto a precio, costo de reposición u otras ajenas a lo expresamente acordado entre las partes, será motivo de diferimiento.

Concordancias:

CODIGO CIVIL (LIBRO IV), Arts. 1740

Art. 19.- Indicación del Precio.- Los proveedores deberán dar conocimiento al público de los valores finales de los bienes que expendan o de los servicios que ofrezcan, con excepción de los que por sus características deban regularse convencionalmente.

El valor final deberá indicarse de un modo claramente visible que permita al consumidor, de manera efectiva, el ejercicio de su derecho a elección, antes de formalizar o perfeccionar el acto de consumo.

El valor final se establecerá y su monto se difundirá en moneda de curso legal.

Las farmacias, boticas, droguerías y similares deberán exhibir de manera visible, además del valor final impreso en cada uno de los medicamentos o bienes de expendio, la lista de precios oficiales de los medicamentos básicos, aprobados por la autoridad competente.

Concordancias:

CODIGO CIVIL (LIBRO IV), Arts. 1747

Art. 20.- Defectos y Vicios Ocultos.- El consumidor podrá optar por la rescisión del contrato, la reposición del bien o la reducción del precio, sin perjuicio de la indemnización por daños y perjuicios, cuando la cosa objeto del contrato tenga defectos o vicios ocultos que la hagan inadecuada o disminuyan de tal modo su calidad o la posibilidad del uso al que habitualmente se le destine, que, de haberlos conocido el consumidor, no la habría adquirido o hubiera dado un menor precio por ella.

Concordancias:

CODIGO CIVIL (LIBRO IV), Arts. 1505, 1698, 1777, 1797, 1800

Art. 21.- FACTURAS.- El proveedor está obligado a entregar al consumidor, factura que documente el negocio realizado, de conformidad con las disposiciones que en esta materia establece el ordenamiento jurídico tributario.

En caso de que al momento de efectuarse la transacción, no se entregue el bien o se preste el servicio, deberá extenderse un comprobante adicional firmado por las partes, en el que constará el lugar y la fecha en la que se lo hará y las consecuencias del incumplimiento o retardo.

En concordancia con lo previsto en los incisos anteriores, en el caso de prestación de servicios, el comprobante adicional deberá detallar además, los componentes y materiales que se empleen con motivo de la prestación del servicio, el precio por unidad de los mismos y de la mano de obra; así como los términos en que el proveedor se obliga, en los casos en que el uso práctico lo permita.

Concordancias:

LEY ORGANICA DE REGIMEN TRIBUTARIO INTERNO, LORTI, Arts. 103

CODIGO CIVIL (LIBRO IV), Arts. 1764, 1766

Art. 22.- Reparación Defectuosa.- Cuando un bien objeto de reparación presente defectos relacionados con el servicio realizado e imputables al prestador del mismo, el consumidor tendrá derecho, dentro de los noventa días contados a partir de la recepción del bien, a que se le repare sin costo adicional o se reponga el bien en un plazo no superior a treinta días, sin perjuicio a la indemnización que corresponda.

Si se hubiere otorgado garantía por un plazo mayor, se estará a este último.

Art. 23.- Deterioro de los Bienes.- Cuando el bien objeto del servicio de acondicionamiento, reparación, limpieza u otro similar sufriere tal menoscabo o deterioro que disminuya su valor o lo torne parcial o totalmente inapropiado para el uso normal al que está destinado, el prestador del servicio deberá restituir el valor del bien, declarado en la nota de ingreso, e indemnizar al consumidor por la pérdida ocasionada.

Art. 24.- Repuestos.- En los contratos de prestación de servicios cuyo objeto sea la reparación de cualquier tipo de bien, se entenderá implícita la obligación de cargo del prestador del servicio, de emplear en tal reparación, componentes o repuestos nuevos y adecuados al bien de que se trate, a excepción de que las partes convengan expresamente lo contrario.

El incumplimiento de esta obligación dará lugar, además de las sanciones e indemnizaciones que correspondan, a que se obligue al prestador del servicio a sustituir, sin cargo adicional alguno, los componentes o repuestos de que se trate.

Art. 25.- Servicio Técnico.- Los productores, fabricantes, importadores, distribuidores y comerciantes de bienes deberán asegurar el suministro permanente de componentes, repuestos y servicio técnico, durante el lapso en que sean producidos, fabricados, ensamblados, importados o distribuidos y posteriormente, durante un período razonable de tiempo en función a la vida útil de los bienes en cuestión, lo cual será determinado de conformidad con las normas técnicas del Instituto Ecuatoriano de Normalización - INEN.

Art. 26.- Reposición.- Se considerará un solo bien, aquel que se ha vendido como un todo, aunque esté formado por unidades, partes, piezas o módulos, no obstante que estas puedan o no prestar una utilidad en forma independiente unas de otras. Sin perjuicio de ello, tratándose de su reposición, esta se podrá efectuar respecto de una unidad, parte, pieza o módulo, siempre que sea por otra igual a la que se restituya y se garantice su funcionalidad.

Art. 27.- Servicios Profesionales.- Es deber del proveedor de servicios profesionales, atender a sus clientes con calidad y sometimiento estricto a la ética profesional, la ley de su profesión y otras conexas.

En lo relativo al cobro de honorarios, el proveedor deberá informar a su cliente, desde el inicio de su gestión, el monto o parámetros en los que se regirá para fijarlos dentro del marco legal vigente en la materia y guardando la equidad con el servicio prestado.

Concordancias:

CODIGO DE PROCEDIMIENTO CIVIL, Arts. 847

CODIGO CIVIL (LIBRO IV), Arts. 2021

Art. 28.- Responsabilidad Solidaria y Derecho de Repetición.- Serán solidariamente responsables por las indemnizaciones civiles derivadas de los daños ocasionados por vicio o defecto de los bienes o servicios prestados, los productores, fabricantes, importadores, distribuidores, comerciantes, quien haya puesto su marca en la cosa o servicio y, en general, todos aquellos cuya participación haya influido en dicho daño.

La responsabilidad es solidaria, sin perjuicio de las acciones de repetición que correspondan. Tratándose de la devolución del valor pagado, la acción no podrá intentarse sino respeto del vendedor final.

El transportista solo responderá por los daños ocasionados al bien con motivo o en ocasión del servicio por él prestado.

Concordancias:

CONSTITUCION DE LA REPUBLICA DEL ECUADOR, Arts. 11

CODIGO CIVIL (LIBRO IV), Arts. 1530, 1538, 1628, 1950, 2274

CODIGO DE COMERCIO, Arts. 221, 235, 244

Art. 29.- Derecho de Repetición del Estado.- Cuando el Estado ecuatoriano sea condenado al pago de cualquier suma de dinero por la violación o inobservancia de los derechos consagrados en la presente ley por parte de un funcionario público, el Estado tendrá derecho de repetir contra dicho funcionario lo efectivamente pagado.

Concordancias:

CONSTITUCION DE LA REPUBLICA DEL ECUADOR, Arts. 11, 54

Art. 30.- Resolución.- La mora en el cumplimiento de las obligaciones a cargo del proveedor de bienes o servicios, permitirá al consumidor pedir la resolución del contrato, sin perjuicio de las indemnizaciones que pudieren corresponder.

Concordancias:

CODIGO CIVIL (LIBRO IV), Arts. 1505, 1561, 1567

Art. 31.- Prescripción de las Acciones.- Las acciones civiles que contempla esta Ley prescribirán en el plazo de doce meses contados a partir de la fecha en que se ha recibido el bien o terminado de prestar el servicio.

Si se hubiese otorgado garantía por un plazo mayor, se estará a éste, para efectos de prescripción.

Concordancias:

CODIGO CIVIL (LIBRO IV), Arts. 2393

CAPITULO VI
SERVICIOS PUBLICOS DOMICILIARIOS

Art. 32.- Obligaciones.- Las empresas encargadas de la provisión de servicios públicos domiciliarios, sea directamente o en virtud de contratos de concesión, están obligadas a prestar servicios eficientes, de calidad, oportunos, continuos y permanentes a precios justos.

Concordancias:

CONSTITUCION DE LA REPUBLICA DEL ECUADOR, Arts. 85

CODIGO ORGANICO DE ORGANIZACION TERRITORIAL, COOTAD, Arts. 55, 137

Art. 33.- Información al Consumidor.- Las condiciones, obligaciones, modificaciones y derechos de las partes en la contratación del servicio público domiciliario, deberán ser cabalmente conocidas por ellas en virtud de la celebración de un instrumento escrito. Sin perjuicio de dicho instrumento, los proveedores de servicios públicos domiciliarios mantendrán dicha información a disposición permanente de los consumidores en las oficinas de atención al público.

El consumidor tiene el derecho de ser oportuna y verazmente informado sobre la existencia o no de seguros accesorios al contrato de prestación del servicio, cobertura y demás condiciones. En caso de seguros de vida, su monto nunca podrá ser menor al establecido en el Código del Trabajo.

Sin perjuicio de la cobertura que los seguros accesorios den para el caso de muerte o perjuicio a la salud del consumidor, la empresa proveedora de servicios públicos domiciliarios, será directamente

responsable de indemnizar por los daños causados a los consumidores por negligencia o mala calidad en la prestación de dichos servicios.

Concordancias:

CONSTITUCION DE LA REPUBLICA DEL ECUADOR, Arts. 11

CODIGO CIVIL (LIBRO IV), Arts. 1576, 1719

CODIGO DE PROCEDIMIENTO CIVIL, Arts. 191, 195

CODIGO DEL TRABAJO, Arts. 369

LEY ORGANICA DE EMPRESAS PUBLICAS, LOEP, Arts. 2

Art. 34.- Reciprocidad.- Las empresas proveedoras de servicios públicos domiciliarios están en la obligación de otorgar un trato recíproco a los consumidores, aplicando en lo referente a reintegros y devoluciones, los mismos criterios que se utilicen para los recargos por mora en el pago del servicio.

Concordancias:

CODIGO CIVIL (LIBRO IV), Arts. 1568

Art. 35.- Registro de Reclamos.- Las empresas proveedoras de servicios públicos domiciliarios deben contar con una oficina y un registro de reclamaciones en donde constarán las presentadas por los consumidores. Dichos reclamos deberán ser subsanados en el plazo perentorio que contendrá el Reglamento a la presente Ley.

Art. 36.- Seguridad de las Instalaciones.- Los consumidores de servicios públicos que se prestan a domicilio y requieren instalaciones específicas, deben ser convenientemente informados sobre las condiciones de seguridad de las instalaciones y de los artefactos.

Art. 37.- Instrumentos y Unidades de Medición.- La autoridad competente queda facultada para intervenir de oficio, o a petición de parte interesada, en la verificación del buen funcionamiento de los instrumentos de medición de energía, combustible, comunicaciones, agua potable, o cualquier otro similar, cuando existan dudas sobre las lecturas efectuadas por las empresas proveedoras del servicio.

Tanto los instrumentos como las unidades de medición deberán ser legalmente reconocidos y autorizados. Las empresas proveedoras del servicio garantizarán al consumidor el control individual de los consumos. Las facturas deberán ser entregadas al usuario con no menos de diez días de anticipación a su vencimiento.

Art. 38.- Interrupción de la Prestación del Servicio.- Cuando la prestación del servicio público domiciliario se interrumpa o sufra alteraciones, por causas imputables al proveedor, éste deberá reintegrar los valores cobrados por servicios no prestados, dentro del plazo de 30 días, contados desde la fecha en que se realice el reclamo. Sin perjuicio de lo señalado, el proveedor reconocerá los daños y perjuicios ocasionados al consumidor por la alteración o interrupción culposa del servicio.

Concordancias:

CONSTITUCION DE LA REPUBLICA DEL ECUADOR, Arts. 54

CODIGO CIVIL (TITULO PRELIMINAR), Arts. 29

CODIGO CIVIL (LIBRO IV), Arts. 1571, 1572

Art. 39.- Facturación de Consumo Excesivo.- Cuando el consumidor considere que existe facturación excesiva en la planilla de un período, podrá cancelar únicamente un valor equivalente al promedio del consumo mensual de los 6 meses inmediatamente anteriores.

Para poder ejercer este derecho, el consumidor debe presentar hasta dentro de los 10 días posteriores al vencimiento de la factura o planilla, las correspondientes al período de 6 meses inmediatos anteriores a la objetada. De no contar con los documentos anotados, el consumidor podrá solicitar las respectivas copias a la empresa prestadora del servicio, en cuyo caso, el plazo anotado comenzará a correr desde la fecha en que se entreguen las copias.

La empresa proveedora del servicio dispondrá de un plazo de treinta días a partir del reclamo del usuario para acreditar que el consumo facturado fue efectivamente realizado, en cuyo caso tendrá derecho a reclamar el pago de la diferencia más los intereses legales correspondientes.

Si el pago efectuado por el consumidor en ejercicio del derecho contemplado en el inciso primero del presente artículo excede del valor real de consumo, la empresa otorgará un crédito idéntico a dicho exceso a favor del consumidor, el mismo que deberá hacerse efectivo en la planilla inmediata posterior.

Mientras se desarrolle el trámite previsto en los incisos precedentes, la empresa proveedora estará obligada a seguir prestando el servicio sin interrupción alguna.

Concordancias:

CODIGO CIVIL (LIBRO IV), Arts. 1611, 2110, 2112

CODIGO DE PROCEDIMIENTO CIVIL, Arts. 173, 175

Art. 40.- Valores de las Planillas.- En las planillas emitidas por las empresas proveedoras de los servicios públicos domiciliarios, deberá constar exclusivamente el valor del consumo respectivo, más los recargos legales pertinentes y cobros adicionales establecidos expresamente por leyes y ordenanzas. Queda prohibido incluir en dichas planillas rubros adicionales a los señalados.

Es un derecho del consumidor el conocer el valor exacto que debe cancelar por concepto de consumo y recargos legales adicionales, por tanto, queda prohibido el planillaje en base de sistemas diferentes a la medición directa, tales como valores presuntivos o estimativos, con excepción del sector rural que no disponga de instrumentos de medición. Por excepción, en caso de pérdida, daño o imposibilidad física de acceder al sistema de medición, la planilla correspondiente al período inmediatamente posterior al momento del daño, podrá ser emitida por un valor equivalente al promedio mensual de los 6 períodos inmediatamente anteriores. En tal caso, es obligación de quien presta el servicio público domiciliario, reparar o reponer el sistema de medición respectivo o notificar al consumidor sobre la imposibilidad física de acceder al medidor para que éste solucione dicha situación, con la finalidad de que la factura o planilla del siguiente período sea emitida en función de datos reales. Si quien presta el servicio no cumple con la obligación de reparar o reponer el sistema de medición, en ningún caso los montos de las planillas de los períodos posteriores podrán ser aumentados presuntiva o estimativamente, siendo obligación del consumidor en los siguientes períodos, pagar exclusivamente un valor igual al del promedio mensual de las planillas de los 6 meses inmediatamente anteriores.

Los proveedores de servicios públicos domiciliarios que sufrieren pérdidas por deficiencias técnicas, u otras causas debidamente comprobadas, imputables a la empresa, deberán asumirlas en su totalidad, quedando prohibido el traslado de dichas pérdidas a las planillas de los consumidores.

Concordancias:

CONSTITUCION DE LA REPUBLICA DEL ECUADOR, Arts. 52

CODIGO CIVIL (TITULO PRELIMINAR), Arts. 9

CAPITULO VII
PROTECCION CONTRACTUAL

Art. 41.- El Contrato de Adhesión.- El contrato de adhesión deberá estar redactado con caracteres legibles, no menores a un tamaño de fuente de diez puntos, de acuerdo a las normas informáticas internacionales, en términos claros y comprensibles y no podrá contener remisiones a textos o documentos que, no siendo de conocimiento público, no se faciliten al consumidor previamente a la celebración del contrato.

Cuando en un contrato de adhesión escrito con determinado tamaño de caracteres existiese además, textos escritos con letras o números significativamente más pequeños, éstos se entenderán como no escritos. Las partes tienen derecho de que se les entregue copias debidamente suscritas y sumilladas de los contratos y todos sus anexos. Si no fuere posible hacerlo en el acto por carecer de alguna firma, el proveedor entregará de inmediato una copia con la constancia de ser fiel al original suscrito por éste; la copia así entregada se tendrá por el texto fidedigno de lo pactado para todos los efectos legales.

Concordancias:

CODIGO DE PROCEDIMIENTO CIVIL, Arts. 173

Art. 42.- Idioma Oficial.- Los contratos de adhesión relativos a las actividades regidas por la presente Ley, deberán estar escritos en idioma castellano, salvo aquellas palabras de otro idioma que el uso haya incorporado al léxico. Las cláusulas que no cumplan con dichos requisitos, no producirán efecto alguno respecto del consumidor.

Sin perjuicio de lo dispuesto en el inciso anterior, en los contratos impresos o formularios prevalecerán las cláusulas que se agreguen, por sobre las del formulario, siempre que el consumidor lo apruebe por escrito. Las condiciones de la oferta se entienden siempre incorporadas al contrato.

Concordancias:

CODIGO CIVIL (LIBRO IV), Arts. 1499, 1505, 1561

Art. 43.- Cláusulas Prohibidas.- Son nulas de pleno derecho y no producirán efecto alguno las cláusulas o estipulaciones contractuales que:

1. Eximan, atenúen o limiten la responsabilidad de los proveedores por vicios de cualquier naturaleza de los bienes o servicios prestados;
2. Impliquen renuncia a los derechos que esta ley reconoce a los consumidores o de alguna manera limiten su ejercicio;
3. Inviertan la carga de la prueba en perjuicio del consumidor;
4. Impongan la utilización obligatoria de un arbitraje o mediación, salvo que el consumidor manifieste de manera expresa su consentimiento;
5. Permitan al proveedor la variación unilateral del precio o de cualquier condición del contrato;
6. Autoricen exclusivamente al proveedor a resolver unilateralmente el contrato, suspender su ejecución o revocar cualquier derecho del consumidor nacido del contrato, excepto cuando tal resolución o modificación esté condicionada al incumplimiento imputable al consumidor;
7. Incluyan espacios en blanco, que no hayan sido llenados o utilizados antes de que se suscriba el contrato, o sean ilegibles;
8. Impliquen renuncia por parte del consumidor, de los derechos procesales consagrados en esta Ley, sin perjuicio de los casos especiales previstos en el Código de Procedimiento Civil, Código de Comercio, Ley de Arbitraje y Mediación y demás leyes conexas; y,
9. Cualesquiera otras cláusula o estipulación que cause indefensión al consumidor o sean contrarias al orden público y a las buenas costumbres.

Lo determinado en el presente artículo incluye a los servicios que prestan las Instituciones del Sistema Financiero.

Concordancias:

CONSTITUCION DE LA REPUBLICA DEL ECUADOR, Arts. 11

CODIGO DE PROCEDIMIENTO CIVIL, Arts. 113, 171

CODIGO CIVIL (LIBRO IV), Arts. 1561, 1576, 1698

Art. 44.- Terminación Anticipada.- En los contratos de adhesión referentes a la prestación de servicios tales como, telefonía celular, medicina prepagada, televisión satelital o por cable u otros similares, el consumidor podrá dar por terminado unilateralmente el contrato en cualquier tiempo, previa notificación por escrito con al menos quince días de anticipación a la finalización del período en curso. En estos casos, en el contrato de adhesión no se podrá incluir cláusulas ni disposición alguna que impongan al consumidor multas, sanciones o recargos de ninguna naturaleza, atribuida a la terminación anticipada de dicho contrato y de incluirlas no tendrán ningún efecto jurídico.

Sin perjuicio de lo dispuesto en el presente artículo, el consumidor mantendrá la obligación de cancelar los saldos pendientes únicamente por servicios efectivamente prestados hasta la fecha de terminación unilateral del contrato, así como los valores adeudados por la adquisición de los bienes necesarios para la prestación del servicio, de ser el caso.

Concordancias:

CODIGO CIVIL (LIBRO IV), Arts. 1505, 1576, 1800

Art. 45.- Derecho de Devolución.- El consumidor que adquiera bienes o servicios por teléfono, catálogo, televisión, internet o a domicilio, gozará del derecho de devolución, el mismo que deberá ser ejercido dentro de los tres días posteriores a la recepción del bien o servicio, siempre y cuando lo permita su naturaleza y el estado del bien sea el mismo en el que lo recibió. En el caso de servicios, el derecho de devolución se ejercerá mediante la cesación inmediata del contrato de provisión del servicio.

Concordancias:

CODIGO CIVIL (LIBRO IV), Arts. 1763

CODIGO DE COMERCIO, Arts. 171, 172

Art. 46.- Promociones y Ofertas.- Toda promoción u oferta especial deberá señalar, además del tiempo de duración de la misma, el precio anterior del bien o servicio y el nuevo precio o, en su defecto, el beneficio que obtendría el consumidor, en caso de aceptarla.

Cuando se trate de promociones en que el incentivo consista en la participación en concursos o sorteos, el anunciante deberá informar al público sobre el monto o número de premios de aquellos, el plazo y el lugar donde se podrán reclamar. El anunciante estará obligado a difundir adecuadamente el resultado de los concursos o sorteos.

Concordancias:

CODIGO DE COMERCIO, Arts. 148

Art. 47.- Sistemas de Crédito.- Cuando el consumidor adquiera determinados bienes o servicios mediante sistemas de crédito, el proveedor estará obligado a informarle en forma previa, clara y precisa:

1. El precio al contado del bien o servicio materia de la transacción;
2. El monto total correspondiente a intereses, la tasa a la que serán calculados; así como la tasa de interés moratoria y todos los demás recargos adicionales;

3. El número, monto y periodicidad de los pagos a efectuar; y,
4. La suma total a pagar por el referido bien o servicio.

Se prohíbe el establecimiento y cobro de intereses sobre intereses. El cálculo de los intereses en las compras a crédito debe hacerse exclusivamente sobre el saldo de capital impago. Es decir, cada vez que se cancele una cuota, el interés debe ser recalculado para evitar que se cobre sobre el total del capital. Lo dispuesto en este artículo y en especial en este inciso, incluye a las instituciones del Sistema Financiero.

El proveedor está en la obligación de conferir recibos por cada pago parcial. El pago de la cuota correspondiente a un período de tiempo determinado hace presumir el de los anteriores.

Concordancias:

CONSTITUCION DE LA REPUBLICA DEL ECUADOR, Arts. 308

CODIGO CIVIL (LIBRO IV), Arts. 1592, 1609, 1611, 2113, 1586

Art. 48.- Pago Anticipado.- En toda venta o prestación de servicios a crédito, el consumidor siempre tendrá derecho a pagar anticipadamente la totalidad de lo adeudado, o a realizar pre-pagos parciales en cantidades mayores a una cuota. En estos casos, los intereses se pagarán únicamente sobre el saldo pendiente.

Lo prescrito en el presente artículo incluye al sistema financiero.

Concordancias:

CODIGO CIVIL (LIBRO IV), Arts. 1511, 1513

Art. 49.- Cobranza de Créditos.- En la cobranza de créditos, el consumidor no deberá ser expuesto al ridículo o a la difamación, ni a cualquier tipo de coacción ilícita ni amenaza de cualquier naturaleza, dirigida a su persona, por el proveedor o quien actúe en su nombre.

La obligación impuesta al proveedor, será exigible, sin perjuicio de las acciones penales a las que hubiere lugar.

Concordancias:

CODIGO CIVIL (LIBRO II), Arts. 973

CODIGO CIVIL (LIBRO IV), Arts. 1467, 1473

CODIGO PENAL, Arts. 596

Art. 50.- Pagos con Tarjeta de Crédito.- El precio para el pago con tarjeta de crédito, será el mismo precio que al contado.

Toda oferta, promoción, rebaja o descuento exigible respecto a la modalidad de pago al contado, será también exigible por el consumidor que efectúa pagos mediante el uso de tarjetas de crédito, salvo que se ponga en su conocimiento oportuna y adecuadamente, en la publicidad o información respectiva y de manera expresa, lo contrario.

CAPITULO VIII
CONTROL DE LA ESPECULACION

Art. 51.- Sin perjuicio de lo que al respecto establecen las normas penales queda absolutamente prohibida la especulación. Igualmente queda prohibida cualquier otra práctica desleal que tienda o sea causa del alza indiscriminada de precios de bienes y/o servicios.

Así mismo, se adoptarán las medidas necesarias para evitar la fuga de alimentos fuera del territorio nacional, que pudieran provocar desabastecimiento de los mercados internos.

Concordancias:

CONSTITUCION DE LA REPUBLICA DEL ECUADOR, Arts. 335, 336

LEY ORGANICA DE EMPRESAS PUBLICAS, LOEP, Arts. 2

Art. 52.- El INEC o el Organismo que haga sus veces elaborará mensualmente, en base de criterios netamente técnicos, el Indice Oficial de Inflación, el Indice de Precios al Productor y el Indice de Precios al Consumidor.

Art. 53.- Cuando se detecte indicios de procesos especulativos los Intendentes de Policía, Subintendentes de Policía, Comisarios Nacionales y demás autoridades competentes, a petición de cualquier interesado o aún de oficio podrán realizar los controles necesarios a fin de establecer la existencia de tales procesos especulativos.

Art. 54.-Nota: Artículo derogado por Ley No. 0, publicada en Registro Oficial Suplemento 555 de 13 de Octubre del 2011 .

CAPITULO IX
PRACTICAS PROHIBIDAS

Art. 55.- Constituyen prácticas abusivas de mercado, y están absolutamente prohibidas al proveedor, entre otras, las siguientes:

1. Condicionar la venta de un bien a la compra de otro o a la contratación de un servicio, salvo que por disposición legal el consumidor deba cumplir con algún requisito;
2. Rehusar atender a los consumidores cuando su stock lo permita;
3. Enviar al consumidor cualquier servicio o producto sin que éste lo haya solicitado. En tal hipótesis, se entenderán como muestras gratis los bienes y/o servicios enviados;
4. Aprovecharse dolosamente de la edad, salud, instrucción o capacidad del consumidor para venderle determinado bien o servicio;
5. Colocar en el mercado productos u ofertar la prestación de servicios que no cumplan con las normas técnicas y de calidad expedidas por los órganos competentes;
6. Aplicar fórmulas de reajuste diversas a las legales o contractuales;
7. Dejar de fijar plazo para el cumplimiento de sus obligaciones, o dejarlo a su único criterio; y,
8. El redondeo de tiempos para efectivizar el cobro de intereses, multas u otras sanciones económicas en tarjetas de crédito, préstamos bancarios y otros similares.

CAPITULO X
PROTECCION A LA SALUD Y SEGURIDAD

Art. 56.- Supletoriedad.- Las disposiciones del presente Capítulo sólo se aplicarán en lo no previsto por las normas especiales que regulan la provisión de determinados bienes o servicios que por sus características deban sujetarse a un tratamiento especial.

Art. 57.- Advertencias Permanentes.- Tratándose de productos cuyo uso resulte potencialmente peligroso para la salud o integridad física de los consumidores, para la seguridad de sus bienes o del ambiente el proveedor deberá incorporar en los mismos, o en instructivos anexos, las advertencias o indicaciones necesarias para que su empleo se efectúe con la mayor seguridad posible.

En cuanto al expendio de bebidas alcohólicas, cigarrillos y otros derivados del tabaco y productos nocivos para la salud, deberá expresarse clara, visible y notablemente la indicación de que su consumo es peligroso para la salud, de acuerdo a lo que al respecto regule el Reglamento a la presente Ley. Dicha advertencia deberá constar, además, en toda la publicidad del bien considerado

como nocivo.

En lo que se refiere a la presentación de servicios riesgosos, deberán adoptarse por el proveedor las medidas que resulten necesarias para que aquella se realice en adecuadas condiciones de seguridad, informando al usuario y a quienes pudieren verse afectados por tales riesgos, de las medidas preventivas que deban usarse.

Concordancias:

CONSTITUCION DE LA REPUBLICA DEL ECUADOR, Arts. 32

CODIGO PENAL, Arts. 428, 429

LEY ORGANICA DE SALUD, Arts. 42, 47

Art. 58.- Productos Riesgosos.- En caso de constatarse que un bien de consumo adolece de un defecto o constituye un peligro o riesgo de importancia para la integridad física, la seguridad de las personas o del medio ambiente, aún cuando se utilice en forma adecuada, el proveedor del mismo deberá, sin perjuicio de las responsabilidades a las que hubiere lugar, informar de tal hecho a los consumidores del bien, retirarlo del mercado y, cuando sea procedente, sustituirlo o reemplazarlo a su costo.

Concordancias:

CONSTITUCION DE LA REPUBLICA DEL ECUADOR, Arts. 32

CODIGO PENAL, Arts. 437

Art. 59.- Prohibición de Comercialización.- Comprobada por cualquier medio idóneo, la peligrosidad o toxicidad de un producto destinado al consumo humano, en niveles considerados como nocivos o peligrosos para la salud del consumidor, la autoridad competente dispondrá el retiro inmediato de dicho bien o producto del mercado y la prohibición de circulación del mismo.

Los daños y perjuicios producidos por la acción de dichos bienes o productos serán de cargo del proveedor, sin perjuicio de la responsabilidad penal a que hubiere lugar.

Concordancias:

CODIGO PENAL, Arts. 437

LEY ORGANICA DE SALUD, Arts. 6

CONSUMO DEL TABACO Y SUS DERIVADOS
Nota: Título aclaratorio no especificado en Registro Oficial.

Art. ....- Se prohíbe el consumo de cigarrillo y otros productos derivados del tabaco en el interior de sitios públicos que, por sus características, propicien el consumo pasivo, esto es: restaurantes, aeropuertos, cines, ascensores, teatros, auditorios, coliseos, estadios, instalaciones destinadas a prácticas deportivas y recreativas; oficinas públicas y dependencias que prestan servicios públicos como: bancos, supermercados, correos; hospitales, clínicas, centros de salud, consultorios médicos, predios, aulas y edificaciones de establecimientos educativos pre-primarios, primarios, secundarios, en las aulas y edificios de las instituciones de educación superior, sean éstos públicos o privados; centros comerciales, como locales que están destinados a la práctica de cultos religiosos y medios de transporte públicos, cualquiera que fuese su tipo en rutas nacionales.

Nota: Artículo agregado por Ley No. 54, publicada en Registro Oficial 356 de 14 de Septiembre del 2006 .
Nota: Artículo derogado por Ley No. 00, publicada en Registro Oficial 497 de 22 de Julio del 2011 .

Concordancias:

LEY ORGANICA DE SALUD, Arts. 43

Art. ....- Queda prohibida la creación de zonas para fumadores dentro de los lugares descritos en el artículo anterior, excepto en terminales de transporte aéreo, terrestre y marítimo, en donde podrán crearse salas especiales para fumadores.

Nota: Artículo agregado por Ley No. 54, publicada en Registro Oficial 356 de 14 de Septiembre del 2006 .
Nota: Artículo derogado por Ley No. 00, publicada en Registro Oficial 497 de 22 de Julio del 2011 .

Art. ....- Excepcionalmente se tolerará el consumo de cigarrillo en las instalaciones de bares, discotecas, casinos y centros de diversión nocturna. En hoteles, además de los sitios descritos anteriormente, únicamente en habitaciones cerradas y determinadas para fumadores. Todos estos lugares deberán tener sistemas de ventilación o aislamientos adecuados que permitan garantizar la calidad de aire para los no fumadores.

Las instalaciones a que hace referencia el inciso precedente, para su funcionamiento deberán contar con el permiso y calificación de la autoridad sanitaria nacional, para garantizar la no contaminación del aire a los no fumadores.

Nota: Artículo agregado por Ley No. 54, publicada en Registro Oficial 356 de 14 de Septiembre del 2006 .
Nota: Artículo derogado por Ley No. 00, publicada en Registro Oficial 497 de 22 de Julio del 2011 .

Art. ....- Las infracciones a los artículos anteriores serán sancionadas con multa de cincuenta dólares de los Estados Unidos de América, sin perjuicio de que el representante legal del establecimiento o institución, asuma la responsabilidad solidaria por omisión en los términos señalados en la presente Ley.

Nota: Artículo agregado por Ley No. 54, publicada en Registro Oficial 356 de 14 de Septiembre del 2006 .
Nota: Artículo derogado por Ley No. 00, publicada en Registro Oficial 497 de 22 de Julio del 2011 .

Concordancias:

CODIGO CIVIL (LIBRO IV), Arts. 1538, 2064

Art. ....- Las cajetillas y el material de embalaje o envolturas de cigarrillos y de otros productos derivados del tabaco que se utilicen para el expendio al público, deberán llevar la siguiente advertencia general: "VENTA PROHIBIDA A NIÑOS, NIÑAS Y ADOLESCENTES. MINISTERIO DE SALUD PUBLICA DEL ECUADOR", escrita en letra helvética mayúscula de 10.8 puntos, impresa en uno de los laterales de la cajetilla y del material de embalaje, en forma legible, clara, y usando colores de alto contraste contra un fondo blanco.

Las cajetillas y el material de embalaje de cigarrillos y de otros productos derivados del tabaco deberán llevar además esta advertencia: "Fumar Causa Cáncer", en letra impresa en el 40% del área del panel frontal de la cajetilla. La advertencia: "Fumar Mata" se colocará en el 40% del área del panel posterior de la cajetilla. Las áreas para impresión de las advertencias, correspondientes al 40%, tanto del panel frontal como del panel posterior, estarán definidas por el ancho del panel y por una altura igual al 40% del alto total del panel correspondiente, medido a partir de la base de la cajetilla. Los textos de ambas advertencias deben quedar paralelos a la base de la cajetilla. Se imprimirán en letra helvética bold de 28 puntos para cajetillas de 20 cigarrillos y helvética bold de 26 puntos para las cajetillas de 10 cigarrillos, en forma legible, clara y usando color contrastante contra

un fondo blanco.

El Ministerio de Educación y Cultura, en coordinación con el Ministerio de Salud Pública, en el ámbito de la educación para la salud, elaborará programas planes y proyectos de prevención del tabaquismo, e iniciará una campaña educativa a través de los medios de comunicación social.

Nota: Artículo agregado por Ley No. 54, publicada en Registro Oficial 356 de 14 de Septiembre del 2006 .
Nota: Artículo derogado por Ley No. 00, publicada en Registro Oficial 497 de 22 de Julio del 2011 .

Art. ....- La infracción al artículo anterior por parte de las personas jurídicas o naturales que manufacturen o importen productos del tabaco, serán sancionadas con una multa del equivalente a veinte remuneraciones mínimas básicas unificadas. En caso de reincidencia se quintuplicará la multa y se procederá al decomiso y destrucción del producto.

Estas mismas sanciones serán aplicadas al fabricante, importador, distribuidor o vendedor que comercialice cajetillas, paquetes o envolturas de cigarrillos en tamaños distintos a los descritos en el artículo anterior.

Nota: Artículo agregado por Ley No. 54, publicada en Registro Oficial 356 de 14 de Septiembre del 2006 .
Nota: Artículo derogado por Ley No. 00, publicada en Registro Oficial 497 de 22 de Julio del 2011 .

Art. ....- Las sanciones previstas en los artículos innumerados que anteceden, se aplicarán cumpliendo lo dispuesto en el artículo 84 de esta Ley.

Los valores provenientes de las multas que se impongan a los infractores, serán entregados a la Sociedad de Lucha Contra el Cáncer-SOLCA, y distribuidos de conformidad con su Ley, para inversiones en estudios y equipamiento de la institución.

Las cajetillas y el material de embalaje o envolturas de cigarrillos y de otros productos derivados del tabaco que no cumplan la exigencia de impresión y advertencia por la salud previstas en esta Ley, serán decomisados y destruidos por las autoridades competentes.

Concédase derecho de acción pública para denunciar las infracciones antes señaladas.

Nota: Artículo agregado por Ley No. 54, publicada en Registro Oficial 356 de 14 de Septiembre del 2006 .
Nota: Artículo derogado por Ley No. 00, publicada en Registro Oficial 497 de 22 de Julio del 2011 .

Concordancias:

LEY ORGANICA DE REGIMEN TRIBUTARIO INTERNO, LORTI, Arts. 71, 73

Art. ....- Los propietarios de negocios que vendan o distribuyan cigarrillos a menores de edad, serán sancionados con cinco remuneraciones mínimas básicas unificadas; su reincidencia será sancionada con la suspensión de actividades de su negocio, por el lapso de quince días.

Nota: Artículo agregado por Ley No. 54, publicada en Registro Oficial 356 de 14 de Septiembre del 2006 .
Nota: Artículo derogado por Ley No. 00, publicada en Registro Oficial 497 de 22 de Julio del 2011 .

Concordancias:

CODIGO DE LA NIÑEZ Y ADOLESCENCIA, Arts. 248, 253

Art. 60.- Licencias.- Las patentes, autorizaciones, licencias u otros documentos o permisos otorgados por el Estado a ciertos proveedores para la investigación, desarrollo o comercialización de bienes o prestación de servicios que puedan resultar peligrosos o nocivos para la salud del consumidor, en ningún caso eximirán de la responsabilidad por los daños y perjuicios efectivamente ocasionados a dichos consumidores, daños que de conformidad a lo dispuesto por esta u otras leyes, serán de cargo de los proveedores y de todos quienes hayan participado en la cadena de producción, distribución y comercialización de los mencionados bienes.

Concordancias:

LEY DE PROPIEDAD INTELECTUAL, Arts. 126, 248

CAPITULO XI
ASOCIACIONES DE CONSUMIDORES

Art. 61.- Asociación de Consumidores.- Se entenderá por Asociación de Consumidores, toda organización constituida por personas naturales o jurídicas, independientes de todo interés económico, comercial, religioso o político, cuyo objeto sea garantizar y procurar la protección y la defensa de los derechos e intereses de los consumidores; así como promover la información, educación, representación y el respeto de los mismos.

Concordancias:

CONSTITUCION DE LA REPUBLICA DEL ECUADOR, Arts. 55, 66

Art. 62.- Requisitos.- Para poder actuar válida y legítimamente en la promoción y defensa de los derechos que esta Ley consagra, las Asociaciones de Consumidores deberán cumplir, además de los requisitos exigidos por la legislación general, con los siguientes:

1. Obtener su personería jurídica en el Ministerio de Bienestar Social;
2. Conformarse con un número no menor a cincuenta miembros;
3. No incluir como asociados a personas jurídicas que se dediquen a actividades comerciales;
4. Mantenerse al margen de actividades comerciales, religiosas o políticas;
5. No perseguir fines de lucro.
6. No aceptar anuncios de carácter comercial en sus publicaciones; y,
7. No realizar una explotación comercial selectiva en la información y consejos que ofrezcan al consumidor.

Art. 63.- Objetivos.- Entre otros, son objetivos de las Asociaciones de Consumidores:

1. Difundir el conocimiento de las disposiciones de esta Ley y sus disposiciones conexas;
2. Promover y proteger los derechos de los consumidores;
3. Representar los intereses individuales o colectivos de los consumidores ante las autoridades judiciales o administrativas; así como, ante los proveedores, mediante el ejercicio de acciones, recursos, trámites o gestiones a que esta Ley se refiere, cuando esto sea solicitado expresamente por los consumidores;
4. Realizar programas de capacitación, orientación y educación del consumidor;
5. Promover la organización de los consumidores con sentido solidario para proteger sus derechos;
6. Promover el conocimiento sobre el precio, la cantidad, la calidad, peso, medida, rotulado e información de los bienes y servicios;
7. Denunciar la práctica o manejo que atente contra los derechos del consumidor consagrados en la presente Ley;
8. Desarrollar una conciencia ambiental, individual o de grupo sobre las consecuencias del consumo en el ambiente y la necesidad de preservar los recursos naturales; y,
9. Prestar la debida colaboración a las autoridades que requieran de su contingente para la investigación de las infracciones establecidas en la presente Ley.

CAPITULO XII
CONTROL DE CALIDAD

Art. 64.- Bienes y Servicios Controlados.- El Instituto Ecuatoriano de Normalización INEN, determinará la lista de bienes y servicios, provenientes tanto del sector privado como del sector público, que deban someterse al control de calidad y al cumplimiento de normas técnicas, códigos de práctica, regulaciones, acuerdos, instructivos o resoluciones. Además, en base a las informaciones de los diferentes ministerios y de otras instituciones del sector público, el INEN elaborará una lista de productos que se consideren peligrosos para el uso industrial y agrícola y para el consumo. Para la importación y/o expendio de dichos bienes, el ministerio correspondiente, bajo su responsabilidad, extenderá la debida autorización.

Concordancias:

CODIGO PENAL, Arts. 437

Art. 65.- Autorizaciones Especiales.- El Registro Sanitario y los Certificados de venta libre de Alimentos, serán otorgados según lo dispone el Código de la Salud, de conformidad con las normas técnicas, regulaciones, resoluciones y códigos de práctica, oficializados por el Instituto Ecuatoriano de Normalización - INEN - y demás autoridades competentes, y serán controlados periódicamente para verificar que se cumplan los requisitos exigidos para su otorgamiento. Para la introducción de bienes importados al mercado nacional, será requisito indispensable contar con la homologación del Registro Sanitario y de los Permisos de Comercialización otorgados por autoridad competente de su país de origen, según lo dispone el Reglamento a la presente Ley y las demás leyes conexas, salvo los casos de aplicación de acuerdos de reconocimiento mutuo vigentes y los que pudieren entrar en vigencia a futuro entre la República del Ecuador y otros países, en el marco de los procesos de integración.

Concordancias:

LEY ORGANICA DE SALUD, Arts. 138

Art. 66.- Normas Técnicas.- El control de cantidad y calidad se realizará de conformidad con las normas técnicas establecidas por el Instituto Ecuatoriano de Normalización - INEN -, entidad que también se encargará de su control sin perjuicio de la participación de los demás organismos gubernamentales competentes. De comprobarse técnicamente una defectuosa calidad de dichos bienes y servicios, el INEN no permitirá su comercialización; para esta comprobación técnica actuará en coordinación con los diferentes organismos especializados públicos o privados, quienes prestarán obligatoriamente sus servicios y colaboración.

Las normas técnicas no podrán establecer requisitos ni características que excedan las establecidas en los estándares internacionales para los respectivos bienes.

Concordancias:

CODIGO PENAL, Arts. 431

Art. 67.- Delegación.- El Instituto Ecuatoriano de Normalización - INEN - y las demás autoridades competentes, podrán, de acuerdo con la ley y los Reglamentos, delegar la facultad de control de calidad mencionada en el Art. anterior, a los Municipios que cuenten con la capacidad para asumir dicha responsabilidad.

Art. 68.- Unidades de Control.- El Instituto Ecuatoriano de Normalización - INEN - promoverá la creación y funcionamiento de los departamentos de control de calidad, dentro de cada empresa pública o privada, proveedora de bienes o prestadora de servicios. Así mismo, reglamentará la

posibilidad de que alternativamente, se contraten laboratorios de las Universidades y Escuelas Politécnicas o laboratorios privados debidamente calificados para cumplir con dicha labor.

Art. 69.- Capacitación.- El Instituto Ecuatoriano de Normalización - INEN - realizará programas permanentes de educación sobre normas de calidad a los proveedores y consumidores, utilizando, entre otros medios, los de comunicación social, en los espacios que corresponden al Estado según la Ley.

CAPITULO XIII
INFRACCIONES Y SANCIONES

Art. 70.- Sanción General.- Las infracciones a lo dispuesto en esta ley, siempre que no tengan una sanción específica, serán sancionadas con multa de cien a mil dólares de los Estados Unidos de América o su equivalente en moneda de curso legal, y si es del caso, el comiso de los bienes, o la suspensión del derecho a ejercer actividades en el campo de la prestación del servicio o publicidad, sin perjuicio de las demás sanciones a las que hubiere lugar. El pago de las sanciones pecuniarias no libera al proveedor de cumplir con las obligaciones que le impone la ley.

Nota: El artículo 17 del Código Orgánico Integral Penal dispone: "Se considerarán exclusivamente como infracciones penales las tipificadas en este Código. Las acciones u omisiones punibles, las penas o procedimientos penales previstos en otras normas jurídicas no tendrán validez jurídica alguna, salvo en materia de niñez y adolescencia.

Art. 71.- Indemnización, Reparación, Reposición y Devolución.- Los consumidores tendrán derecho, además de la indemnización por daños y perjuicios ocasionados, a la reparación gratuita del bien y, cuando no sea posible, a su reposición o a la devolución de la cantidad pagada, en un plazo no superior a treinta días, en los siguientes casos:

1. Cuando en el producto que se hubiere adquirido con determinada garantía y, dentro del plazo de ella, se pusiere de manifiesto la deficiencia o características del bien garantizado, siempre que se hubiere destinado al uso o consumo normal de acuerdo a la naturaleza de dicho bien. Este derecho se ejercerá siempre y cuando el proveedor haya incumplido con la garantía;
2. Cuando cualquier producto, por sus deficiencias de fabricación, elaboración, estructura, calidad o condiciones sanitarias, en su caso, no sea apto para el uso al cual está destinado; y,
3. Cuando considerados los límites de tolerancia permitidos, el contenido neto de un producto resulte inferior al que debiera ser o la cantidad sea menor a la indicada en el envase o empaque.

Sin perjuicio de las acciones civiles, penales o administrativas a que hubiere lugar, el proveedor que incurriere en uno de los casos contemplados en este artículo, e incumpliere su obligación una vez fenecido el plazo establecido, será sancionado con una multa equivalente al valor del bien o servicio, que en ningún caso será inferior a ciento veinte dólares de los Estados Unidos de América o su equivalente en moneda de curso legal, sin que ello se extinga su obligación de reparar o reponer el bien, o en su caso restituir lo pagado.

Concordancias:

CODIGO CIVIL (LIBRO IV), Arts. 1572

Art. 72.- El proveedor cuya publicidad sea considerada engañosa o abusiva, según lo dispuesto en el Art. 7 de esta Ley, será sancionado con una multa de mil a cuatro mil dólares de los Estados Unidos de América o su equivalente en moneda de curso legal. Cuando un mensaje publicitario sea engañoso o abusivo, la autoridad competente dispondrá la suspensión de la difusión publicitaria, y además ordenará la difusión de la rectificación de su contenido, a costa del anunciante, por los mismos medios, espacios y horarios. La difusión de la rectificación no será menor al treinta por ciento (30%) de la difusión del mensaje sancionado.

Art. 73.- El proveedor que incurra en lo establecido en el artículo 23 de la presente Ley, e incumpla las obligaciones allí establecidas, será sancionado con la clausura temporal o definitiva del establecimiento.

Art. 74.- En caso de incumplimiento a lo dispuesto en el artículo 58 de la presente Ley, el infractor será sancionado con multa de mil a cinco mil dólares de los Estados Unidos de América o su equivalente en moneda de curso legal.

Art. 75.- Servicios Defectuosos.- Cuando los servicios prestados sean manifiestamente defectuosos, ineficaces, causen daño o no se ajusten a lo expresamente acordado, los consumidores tendrán derecho, además de la correspondiente indemnización por daños y perjuicios, a que le sea restituido el valor cancelado. Además, el proveedor de tales servicios, será sancionado con una multa de cincuenta a quinientos dólares de los Estados Unidos de América o su equivalente en moneda de curso legal, sin perjuicio de las demás acciones a que hubiere lugar.

Art. 76.- Espectáculos Públicos.- Serán sancionados con multa equivalente al diez por ciento (10%) del valor recaudado en taquilla, las personas naturales o jurídicas organizadores de espectáculos públicos, incluidos los artísticos y deportivos, que pongan en venta una cantidad de localidades que supere la capacidad de los respectivos recintos; sin perjuicio de su obligación de restituir lo pagado, a quienes, a causa de lo señalado, no hayan podido ingresar al espectáculo.

En caso de reincidencia a lo dispuesto en el presente artículo, la multa será equivalente al veinte por ciento (20%) del valor de la taquilla, sin perjuicio de las sanciones civiles o penales que hubiere lugar.

Concordancias:

CODIGO PENAL, Arts. 606, 629

Art. 77.- Suspensión Injustificada del Servicio.- El que suspendiere, paralizare o no prestare, sin justificación o arbitrariamente, un servicio previamente contratado y por el cual se hubiere pagado derecho de conexión, instalación, incorporación, mantenimiento o tarifa de consumo, será sancionado con una multa de mil a cinco mil dólares de los Estados Unidos de América o su equivalente en moneda de curso legal, sin perjuicio de las demás acciones a las que hubiere lugar.

Adicionalmente el Estado y las Entidades Seccionales Autónomas y/o los concesionarios del ejercicio del derecho para la prestación de servicios, responderán civilmente por los daños y perjuicios ocasionados a las habitantes, por su negligencia y descuido en la atención a la prestación de los servicios públicos que estén a su cargo, y por la carencia de servicios que hayan sido pagados.

Art. 78.- Cobro durante la Suspensión del Servicio.- El proveedor de servicios públicos o privados, no podrá efectuar cobro alguno por el mismo, durante el tiempo en que se encuentre interrumpido y, en todo caso, estará obligado a descontar o reembolsar al consumidor el valor del servicio pagado y no devengado.

Art. 79.- Requerimiento de Información.- Sin perjuicio de la facultad de las autoridades de asistirse por la fuerza pública, será sancionado con multa de quinientos a cinco mil dólares de los Estados Unidos de América o su equivalente en moneda de curso legal, el proveedor que se negare a proporcionar la información requerida por autoridad competente o que proporcionare información falsa.

La misma pena será impuesta al proveedor que impida a la autoridad competente, por cualquier medio, la inspección de los lugares de prestación de servicios, producción, expendio o almacenamiento de bienes, productos o que se oponga a la verificación de la información proporcionada.

Art. 80.- Reincidencia.- En caso de reincidencia en las infracciones que establece la presente ley, la multa señalada podrá ser elevada al doble, además de la clausura temporal o definitiva del establecimiento, se considerará reincidente al proveedor que sea sancionado por una misma infracción a esta Ley, dos veces o más dentro del mismo año calendario.

Para la aplicación de las multas, la autoridad competente tendrá en cuenta de manera especial, la gravedad de la infracción, la cuantía de lo disputado y las condiciones económicas del infractor.

CAPITULO XIV
COMPETENCIA Y PROCEDIMIENTO

Art. 81.- Facultad de la Defensoría del Pueblo.- Es facultad de la Defensoría del Pueblo, conocer y pronunciarse motivadamente sobre los reclamos y las quejas, que presente cualquier consumidor, nacional o extranjero, que resida o esté de paso en el país y que considere que ha sido directa o indirectamente afectado por la violación o inobservancia de los derechos fundamentales del consumidor, establecidos en la Constitución Política de la República, los tratados o convenios internacionales de los cuales forme parte nuestro país, la presente ley, así como las demás leyes conexas.

En el procedimiento señalado en el inciso anterior, la Defensoría del Pueblo podrá promover la utilización de mecanismos alternativos para la solución de conflictos, como la mediación, siempre que dicho conflicto no se refiera a una infracción penal.

Sin perjuicio de lo dispuesto en el presente artículo, el consumidor podrá acudir, en cualquier tiempo, a la instancia judicial o administrativa que corresponda.

Concordancias:

CONSTITUCION DE LA REPUBLICA DEL ECUADOR, Arts. 11, 52, 215

Art. 82.- Procedimiento Ante la Defensoría del Pueblo.- En lo relacionado con tal procedimiento, serán aplicables a las disposiciones del Título III de la Ley Orgánica de la Defensoría del Pueblo, así como las disposiciones reglamentarias que para este efecto dicte el Defensor del Pueblo.

Art. 83.- Informe.- Una vez agotado el procedimiento anterior y, en caso de que las partes no hayan llegado a un acuerdo, la Defensoría del Pueblo elaborará un informe en base del cual solicitará a las autoridades competentes la iniciación del respectivo proceso investigativo del que se podrá desprender la imposición de las sanciones establecidas en la presente Ley, así como la exigencia de que se dé cumplimiento a la obligación pendiente.

El informe emitido por la Defensoría del Pueblo será apreciado por el juez de acuerdo a su sana crítica.

El Defensor del Pueblo podrá acudir ante el Juez de Contravenciones de su respectiva jurisdicción a fin de solicitar el inicio del respectivo proceso.

Art. 84.- Juzgamiento de Infracciones.- Son competentes para conocer y resolver sobre las infracciones a las normas contenidas en la presente Ley, en primera instancia, el Juez de Contravenciones de la respectiva jurisdicción, y, en caso de apelación, el Juez de lo Penal de la respectiva jurisdicción.

El juzgamiento de las infracciones previstas en esta Ley se iniciará mediante denuncia, acusación particular o excitativa fiscal.

Propuesta la denuncia y una vez citado el acusado, el Juez señalará día y hora para la audiencia oral

de juzgamiento, la misma que deberá llevarse a cabo dentro del plazo de diez días contados a partir de la fecha de la notificación. Dicha audiencia iniciará con la contestación del acusado. A esta audiencia concurrirán las partes con todas las pruebas de las que se crean asistidos, previniéndoles que se procederá en rebeldía.

Se dispondrá que las partes presenten sus pruebas, luego de lo cual se dictará sentencia en la misma audiencia, de ser posible, caso contrario se lo hará dentro del plazo perentorio de tres días.

Si el consumidor anexa a su denuncia el informe emitido por la Defensoría del Pueblo, se considerará su contenido de conformidad a lo dispuesto en la presente Ley.

Concordancias:

CODIGO DE PROCEDIMIENTO CIVIL, Arts. 69, 117, 121

Art. 85.- Intervención de Peritos.- Si para el establecimiento de los hechos fuere necesaria, a criterio del juez, la intervención de peritos o se requiriere informes técnicos, se suspenderá la audiencia solo para este objeto y se concederá el plazo de hasta quince días para la presentación de los mismos, al vencimiento del cual, previo señalamiento de día y hora, se reanudará la audiencia y se procederá en la forma en que se indica en el artículo anterior.

Si el peritaje o informe técnico, a criterio del juez, tuviere que practicarse en el exterior, el plazo antes señalado podrá extenderse hasta por treinta días.

Concordancias:

CODIGO DE PROCEDIMIENTO CIVIL, Arts. 257, 262

Art. 86.- De la sentencia que dicte el Juez de contravenciones se podrá interponer el recurso de apelación dentro del término de tres días, contados a partir de la notificación con el fallo. Dicho recurso será presentado ante el juez de contravenciones quien lo remitirá al respectivo Juez de lo penal. La sentencia que dicta el juez de lo penal, causará ejecutoria.

Art. 87.- Daños y Perjuicios.- La sentencia condenatoria lleva implícita la obligación del sentenciado de pagar daños y perjuicios al afectado, costas y honorarios. El cobro de daños y perjuicios se lo hará de conformidad con lo que dispone el artículo 391 del Código de Procedimiento Penal, publicado en el Registro Oficial No. 360, de 13 de enero del 2000 .

Concordancias:

CODIGO DE PROCEDIMIENTO PENAL 2000, Arts. 391

CODIGO CIVIL (LIBRO IV), Arts. 1572

CODIGO DE PROCEDIMIENTO CIVIL, Arts. 828

Art. 88.- Acción Popular.- Se concede acción popular para denunciar las infracciones previstas en esta Ley.

CAPITULO XV
DISPOSICIONES GENERALES

Art. 89.- Planes de Estudio.- El Ministerio de Educación y Cultura incluirá, como eje transversal, dentro del pensum de asignaturas ya existentes, un componente relacionado a la educación del consumidor; con tal finalidad ejecutará programas de capacitación docente e incluirá mensajes acerca de los derechos del consumidor en los textos y otros medios pedagógicos.

La educación del consumidor privilegiará las siguientes áreas:

1. El conocimiento de los derechos y obligaciones;
2. Promover la capacidad para elegir con mayor libertad y eficacia entre los bienes y servicios que ofrece el mercado;
3. Planificar y satisfacer mejor sus necesidades; y,
4. Evitar riesgos derivados de un uso inadecuado de bienes y servicios.

Art. 90.- Difusión Pública.- Para la difusión pública permanente de los derechos, principios y normas establecidas en la presente Ley, la Defensoría del Pueblo, a más de estar plenamente facultada para realizar cualquier tipo de convenios o acuerdos de difusión gratuita con los medios de comunicación, hará uso de los espacios que por ley corresponden al Estado.

Art. 91.- Servicio de Telefonía.- Las empresas públicas o privadas que presten servicios de telefonía fija o móvil celular, bajo ningún concepto podrán aplicar mecanismos de redondeo de tarifas; la facturación se hará por el tiempo real de uso, expresado en minutos y segundos, según corresponda.

Art. 92.- Centros de Acopio.- Las autoridades de los Organismos Seccionales, dentro de sus respectivas jurisdicciones, procurarán y promoverán la creación de centros de acopio en los que los productores podrán comercializar directamente sus productos.

Art. 93.- Intereses por Mora.- Cuando fuere procedente el cobro de intereses por mora en el pago atrasado de facturas y planillas de servicios, el consumidor pagará el interés legal por el tiempo efectivo de mora. Caso contrario, el afectado podrá acudir ante la autoridad competente para la tutela de su derecho.

Concordancias:

CODIGO CIVIL (LIBRO IV), Arts. 1567

Art. 94.- Multas.- Los fondos provenientes de las multas impuestas en cumplimiento de las disposiciones de la presente ley, serán depositadas en la Cuenta Unica del Tesoro Nacional. El equivalente al cincuenta por ciento (50%) de estos recursos se administrarán de conformidad con la Ley de Presupuestos, su reglamento y demás normas técnicas vigentes. El restante cincuenta por ciento (50%) se destinará así: veinte y cinco por ciento (25%) al establecimiento de programas de difusión y capacitación en lo relacionado con la educación del consumidor, que se desarrollarán a través de la Defensoría del Pueblo y las asociaciones de consumidores; y el otro veinte y cinco (25%) al Instituto Ecuatoriano de Normalización - INEN - para el ejercicio de las competencias asignadas en esta Ley.

Art. 95.- Supletoriedad.- En todo lo no previsto en esta Ley, en lo relativo al Procedimiento para el juzgamiento de las infracciones aquí determinadas, se estará a lo que dispone el Código de Procedimiento Civil.

DISPOSICIONES FINALES

PRIMERA.- Derógase la Ley de Defensa del Consumidor, publicada en el Registro Oficial No. 520 de 12 de Septiembre de 1990 y todas sus posteriores reformas. De igual forma, derógase el Reglamento de dicha ley, expedido mediante Decreto No. 2201-A, publicado en el Registro Oficial No. 625 de 19 de febrero de 1991 , y todas sus reformas.

SEGUNDA.- Sustitúyase el artículo 148 del Código de Comercio por el siguiente:

"**Art. 148**.- Las ofertas públicas contenidas en circulares, catálogos, avisos publicitarios, proformas, obligan a quien las hace; salvo que en la misma oferta se señale un determinado plazo de validez de la misma o que las condiciones de la oferta original sean modificadas por una oferta posterior.

TERCERA.- Derógase la Disposición Transitoria Décimo Cuarta de la Ley para la Transformación Económica del Ecuador, publicada en el Registro Oficial No. 34 de 13 de marzo del 2000 .

DISPOSICIONES TRANSITORIAS

PRIMERA.-Nota: Disposición derogada por Ley No. 0, publicada en Registro Oficial Suplemento 544 de 9 de Marzo del 2009 .

SEGUNDA.- Los proveedores tendrán el plazo máximo de nueve meses contados a partir de la promulgación de la presente Ley en el Registro Oficial, para dar cumplimiento a lo dispuesto en los artículos 14, 15 y 16 de esta Ley, tiempo en el que deberán incorporar en sus etiquetas, envases, empaques o manuales de uso de los bienes ofrecidos, los cambios establecidos.

TERCERA.- Durante los noventa días inmediatos posteriores a la vigencia de esta Ley, el Presidente de la República o el Ministro al que delegue para el efecto, realizará la difusión pública de los derechos, obligaciones, principios y normas establecidas en la presente Ley, para lo cual hará uso de los espacios que por Ley le corresponden al Estado, en los diferentes medios de comunicación colectiva.

CUARTA.- Una vez concluido el proceso de modernización del Instituto Ecuatoriano de Normalización - INEN -, este Instituto pasará a constituir una dependencia técnica del Consejo Nacional de Calidad, creado mediante Decreto Ejecutivo No. 401, publicado en el Registro Oficial No. 87 de 30 de mayo del 2000 .

CUARTA-A.- Para la implementación de las normas relativas al empaquetado de cigarrillos u otros productos del tabaco, fabricados o importados, se concederá un plazo improrrogable de 120 días contados desde la fecha de publicación de la presente Ley en el Registro Oficial.

Nota: Disposición dada por Ley No. 54, publicada en Registro Oficial 356 de 14 de Septiembre del 2006 .
Nota: Artículo derogado por Ley No. 00, publicada en Registro Oficial 497 de 22 de Julio del 2011 .

QUINTA.- El Presidente de la República, mediante Decreto Ejecutivo expedirá dentro del plazo de noventa días a partir de la vigencia de esta Ley, el Reglamento General para su aplicación.
